# Annexin A1 Is Required for Efficient Tumor Initiation and Cancer Stem Cell Maintenance in a Model of Human Breast Cancer

**DOI:** 10.3390/cancers13051154

**Published:** 2021-03-08

**Authors:** Cameron N. Johnstone, Yan Tu, Shenna Langenbach, David Baloyan, Andrew D. Pattison, Peter Lock, Kara L. Britt, Brian D. Lehmann, Traude H. Beilharz, Matthias Ernst, Robin L. Anderson, Alastair G. Stewart

**Affiliations:** 1 Department of Pharmacology and Therapeutics, University of Melbourne, Parkville 3010, Australia; yan.tu2@rch.org.au (Y.T.); shennal@unimelb.edu.au (S.L.); 2 Peter MacCallum Cancer Centre, Cancer Research Division, Melbourne 3000, Australia; kara.britt@petermac.org; 3 Sir Peter MacCallum Department of Oncology, University of Melbourne, Melbourne 3000, Australia; robin.anderson@onjcri.org.au; 4 Olivia Newton-John Cancer Research Institute, Heidelberg 3084, Australia; david.baloyan@onjcri.org.au (D.B.); matthias.ernst@onjcri.org.au (M.E.); 5 School of Cancer Medicine, La Trobe University, Heidelberg 3084, Australia; 6 Department of Clinical Pathology, University of Melbourne, Melbourne 3000, Australia; 7 Department of Biochemistry and Molecular Biology, Biomedicine Discovery Institute, Monash University, Clayton 3800, Australia; andrew.pattison@unimelb.edu.au (A.D.P.); traude.beilharz@monash.edu (T.H.B.); 8 La Trobe Bioimaging Platform, La Trobe University, Bundoora 3086, Australia; p.lock@latrobe.edu.au; 9 Vanderbilt-Ingram Cancer Centre, Vanderbilt University School of Medicine, Nashville, TN 37232, USA; brian.d.lehmann@vanderbilt.edu; 10 ARC Centre for Personalised Therapeutics Technologies, University of Melbourne, Parkville 3010, Australia

**Keywords:** Annexin A1, breast cancer, mouse model, allograft, xenograft

## Abstract

**Simple Summary:**

Triple-negative breast cancer (TNBC) has a poor outcome compared to the other major breast cancer subtypes and new therapies are needed. We sought to clarify the functions of a ubiquitous protein, Annexin A1, in the development and progression of TNBC. We found that Annexin A1 expression correlated with poor patient prognosis in basal-like breast tumors and also in the basal like-2 subset of TNBCs. Stable knockdown of Annexin A1 attenuated the growth of SUM149 xenografts, which model basal-like 2 tumors. In a polyoma middle T antigen-driven allograft model of breast cancer, Annexin A1 depletion markedly delayed tumor formation, induced epithelial to mesenchymal transition and upregulated basal markers. Finally, loss of Annexin A1 resulted in the loss of a discrete CD24^+^/Sca1^−^ population containing putative tumor-initiating cells. Collectively, our data demonstrate a novel cell-autonomous role for Annexin A1 in the promotion of tumor-forming capacity in certain TNBC tumors.

**Abstract:**

Triple-negative breast cancer (TNBC) has a poor outcome compared to other breast cancer subtypes, and new therapies that target the molecular alterations driving tumor progression are needed. Annexin A1 is an abundant multi-functional Ca^2+^ binding and membrane-associated protein. Reported roles of Annexin A1 in breast cancer progression and metastasis are contradictory. Here, we sought to clarify the functions of Annexin A1 in the development and progression of TNBC. The association of Annexin A1 expression with patient prognosis in subtypes of TNBC was examined. Annexin A1 was stably knocked down in a panel of human and murine TNBC cell lines with high endogenous Annexin A1 expression that were then evaluated for orthotopic growth and spontaneous metastasis in vivo and for alterations in cell morphology in vitro. The impact of Annexin A1 knockdown on the expression of genes involved in mammary epithelial cell differentia tion and epithelial to mesenchymal transition was also determined. Annexin A1 mRNA levels correlated with poor patient prognosis in basal-like breast tumors and also in the basal-like 2 subset of TNBCs. Unexpectedly, loss of Annexin A1 expression had no effect on either primary tumor growth or spontaneous metastasis of MDA-MB-231_HM xenografts, but abrogated the growth rate of SUM149 orthotopic tumors. In an MMTV-PyMT driven allograft model of breast cancer, Annexin A1 depletion markedly delayed tumor formation in both immuno-competent and immuno-deficient mice and induced epithelial to mesenchymal transition and upregulation of basal markers. Finally, loss of Annexin A1 resulted in the loss of a discrete CD24^+^/Sca1^−^ population containing putative tumor initiating cells. Collectively, our data demonstrate a novel cell-autonomous role for Annexin A1 in the promotion of tumor-forming capacity in a model of human breast cancer and suggest that some basal-like TNBCs may require high endogenous tumor cell Annexin A1 expression for continued growth.

## 1. Introduction

Breast cancer is a common disease accounting for 15% of global cancer-related mortality in women in 2018 [1]. Invasive ductal adenocarcinoma, the most prevalent histological type is sub-classified into hormone receptor (estrogen receptor and progesterone receptor) positive, human epidermal growth factor-2 amplified (HER2+) or referred to as triple-negative subtype (TNBC; triple-negative breast cancer) when lacking expression of all three receptors. Patients with TNBC have a propensity for relapse, development of metastases and poor survival relative to other subtypes [2]. New immunotherapeutic approaches and therapies targeting the molecular dependencies present in these neoplasms are much needed [3]. The application of genome-wide expression profiling has enabled the elucidation of four distinct molecular subtypes, namely, basal-like 1 (BL-1), basal-like 2 (BL-2), mesenchymal (M), and luminal androgen receptor positive (LAR+), thus identifying TNBCs as a heterogeneous classification of tumors [4,5,6,7,8]. Gene expression profiling has also demonstrated an incomplete overlap between TNBCs and the basal-like molecular subtype of breast cancer, which is enriched for basal mammary epithelial markers such as cytokeratin 5, cytokeratin 14, EGFR and TP63 [9,10]. Approximately three quarters of TNBCs are basal-like and three quarters of basal-like tumors are TNBCs [11,12,13].

Annexin A1 is the prototypical member of the Annexin protein family [14], originally described as a cellular effector for the anti-inflammatory actions of glucocorticoids via inhibition of cytosolic phospholipase A2 activity [15,16]. Annexin A1 is abundant in the cytoplasm of many cell types where it may reversibly bind to phospholipid membranes in a Ca^2+^-dependent manner [15]. In cancer, Annexin A1 function depends on its subcellular localization: roles for nuclear, cytoplasmic, membrane-associated, free extracellular, and microvesicle encapsulated forms have been described [17,18,19]. Extracellular Annexin A1 released from either viable or necrotic cancer cells can bind to and activate G-protein-coupled formyl peptide receptors (FPRs) on neoplastic cells to promote mitogenesis in an autocrine manner [18,20,21]. Extracellular or membrane-associated, externally facing Annexin A1 synthesized by cancer cells has many possible functions in the tumor microenvironment. In addition to autocrine action, Annexin A1 can signal in a paracrine or juxtacrine mode to FPRs on tumor-infiltrating leukocytes, such as dendritic cells [22], tumor-associated macrophages [23], neutrophils [24], natural killer (NK) cells [25], or T cells [25,26], which could potentially influence immune cell function to favor tumor growth. For example, Annexin A1 released from tumor cells was shown recently to activate Fpr2 on the surface of regulatory T cells to enhance their immunosuppressive function [26].

In breast cancer, studies of the association of primary tumor Annexin A1 expression levels and patient outcome have yielded some conflicting results [17]. In the normal mammary gland, Annexin A1 is expressed in both the myoepithelial and luminal cell layers, with particularly high expression by myoepithelial cells [27,28,29]. Annexin A1 protein expression as measured by immunohistochemistry is consistently decreased in primary breast cancers relative to normal mammary gland or benign lesions, with expression most commonly retained in poorly differentiated TNBC or basal-like tumors [27,30,31,32]. However, one study found lower Annexin A1 protein expression in metastatic primary human breast cancers compared to non-metastatic and an association between Annexin A1 expression and better metastasis-free and overall patient survival [30]. Others have also shown a better overall survival in patients with Annexin A1 positive primary breast cancers [29].

However, it is now generally accepted that Annexin A1 expression levels are higher in TNBCs and in the basal-like molecular subtype relative to other subtypes [27,28,31,33,34,35], and that elevated Annexin A1 levels are associated with shorter duration of patient survival in TNBC [33,35,36]. An association between high Annexin A1 expression and poor prognosis in the HER2+ subtype was also reported [28,31].

The cellular and molecular functions of Annexin A1 that underpin its association with the aggressive TNBC phenotype and poor patient survival are not completely understood, and some data are conflicting. de Graauw and colleagues demonstrated that Annexin A1 promoted epithelial to mesenchymal transition (EMT), invasion, and metastasis of mouse 4T1 TNBC cells and was able to potentiate TGFβ signaling in both 4T1 cells and human MDA-MB-231 TNBC cells [27], which is consistent with data showing Annexin A1 promotes motility in TNBC cells [33,34,37]. Conversely, stable Annexin A1 knockdown in EpRas mouse mammary cancer cells, driven by an activated Ras oncogene, indicated that endogenous Annexin A1 promoted the epithelial phenotype and also abrogated both orthotopic mammary tumor growth and spontaneous metastasis to lung [30].

Here, we sought to clarify the role of Annexin A1 in TNBC growth and metastasis by comprehensive evaluation of clinical and experimental datasets and by manipulating endogenous Annexin A1 expression in xenograft and allograft models of breast cancer. Annexin A1 expression was evaluated across the different subtypes of TNBC and tested for association with patient survival. Annexin A1 function was investigated through stable shRNA-mediated knockdown in multiple human and mouse TNBC cell lines and by characterization of both primary tumor growth and spontaneous metastasis in immuno-compromised and immuno-competent mice. Annexin A1 was not implicated in the regulation of spontaneous metastasis to distant sites in either xenograft or allograft models. However, in a polyoma virus middle T antigen (PyMT)-driven allograft model of estrogen receptor (ER)-negative breast cancer, we found that while Annexin A1 expression was dispensable for cell survival and proliferation in vitro, it was unexpectedly required for the initiation of tumor growth in vivo and for the maintenance of a luminal mammary epithelial gene expression program. Orthotopic implantation of the Annexin-A1-deficient cells produced dormant viable microscopic tumor foci that eventually attained proliferative capacity leading to the formation of tumors with a predominantly sarcomatoid histology. Moreover, the reduced tumorigenicity of Annexin-A1-depleted cells was associated with almost complete loss of a discrete CD24^+^/Sca1^−^ cell compartment containing putative tumor initiating cells (TICs) that display an epithelial phenotype, and concomitant acquisition of a CD24^−^/Sca1^+^ population with a mesenchymal stem cell-like phenotype.

## 2. Results and Discussion

### 2.1. Annexin 1 Expression Is Associated with Worse Prognosis in Basal-Like Breast Cancer

**Figure 1:**
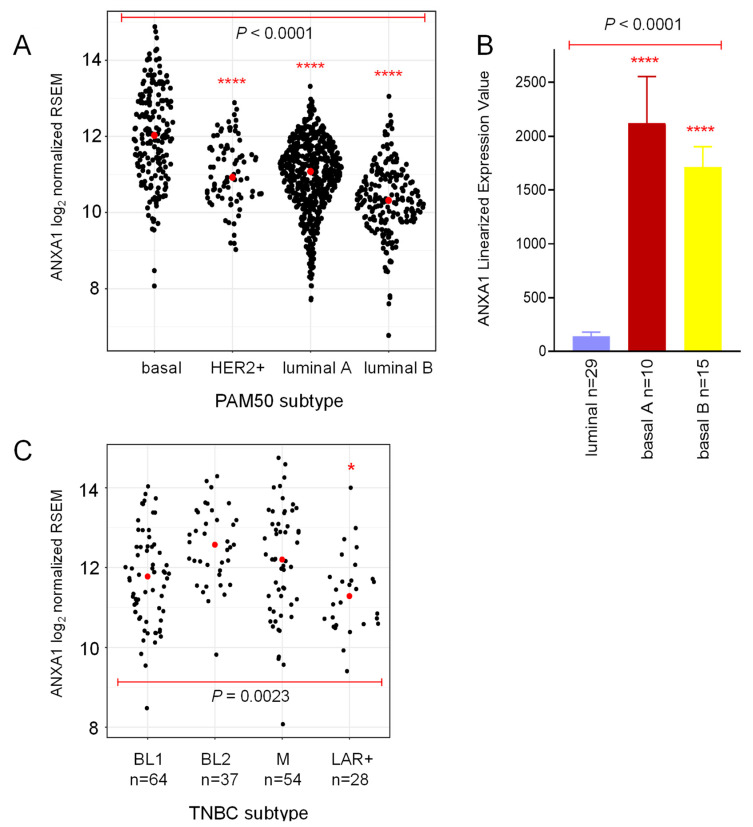
Annexin A1 is highly expressed in basal-like breast cancer. (**A**) Expression of Annexin A1 (log_2_ normalized RSEM) in 1081 primary human breast cancers from the The Cancer Genome Atlas (TCGA) dataset [38]. Tumors were allocated to one of four intrinsic molecular subtypes using the PAM50 gene set [41]. Annexin A1 was differentially expressed across the subtypes (one-way ANOVA *p* < 0.0001) and was significantly higher in basal-like tumors than either HER2+, luminal, or luminal B (each *p* < 0.0001, ****). (**B**) Annexin A1 mRNA levels in human breast cancer cell lines [42]. Mean ± SEM. One-way ANOVA *p* < 0.0001. ER+ luminal v basal A (*p* < 0.0001, ****), luminal v basal B (*p* < 0.0001, ****), basal A v basal B (*p* > 0.05). (**C**) Annexin A1 mRNA expression (log_2_ normalized RSEM) in the four different subtypes of TNBC [5,6]. BL1 (*n* = 64), BL2 (*n* = 37), M (*n* = 54), LAR (*n* = 28). One-way ANOVA *p* = 0.0023. BL2 v LAR (*p* < 0.05, *).

We assessed Annexin A1 mRNA expression levels in 1081 primary breast tumors from the The Cancer Genome Atlas (TCGA) dataset and stratified by intrinsic molecular subtype (Figure 1A) and tested for associations with patient clinical outcome [9,10,38]. First, we confirmed that Annexin A1 expression was significantly higher in basal-like tumors than in either HER2+, luminal A, or luminal B [27,31,33,35]. No differences were found in Annexin A1 expression among HER2+, luminal A, and luminal B tumors (*p* > 0.05). Analysis of a published dataset of breast cancer cell lines showed that Annexin A1 expression was markedly higher in basal A (corresponding to BL-1 and BL-2 whole tumor TNBC subtypes), and basal B (corresponding to the M whole tumor TNBC subtype and claudin-low intrinsic subtype) TNBC lines [12,27,39,40], compared to the estrogen receptor positive (ER+) luminal A and B (luminal) cell lines (Figure 1B, Figure S1), consistent with previous studies [27,33]. In an analysis of 183 primary human TNBCs from TCGA stratified according to subtype, Annexin A1 mRNA was differentially expressed, with the highest expression in the basal-like-2 (BL-2) group, followed by the mesenchymal (M) group (Figure 1C) [5,6]. Annexin A1 expression was lowest in LAR+ tumors, which is not unexpected given that this group of TNBC tumors often display a more luminal gene expression profile driven by activity of the androgen receptor [5].

To determine whether Annexin A1 expression is associated with patient prognosis, relapse-free survival was examined in a combined cohort of 3951 breast cancer cases with 200 months of clinical follow up [43]. While we did not find an association between Annexin A1 expression in primary luminal A/B or HER2+ tumors and disease relapse, we observed strong association with survival in the basal-like intrinsic subtype of tumors (hazard ratio (H.R.) 1.77, *n* = 618, *p* < 0.0001), in agreement with several published reports (Table 1) [33,35,36]. This result was not upheld when all ER− tumors were considered, suggesting factors in addition to the absence of ER expression contribute to the association of Annexin A1 expression with outcome in basal-like tumors. Since about three quarters of basal-like tumors are TNBCs [11,12,13], the relationship between Annexin A1 levels and patient outcome was investigated across the different molecular subtypes of TNBC [5,6]. Higher Annexin A1 expression was associated with worse prognosis in the BL-2 subtype (H.R. 3.12, *n* = 76, *p* = 0.0257) but not in the M or LAR subtypes, and showed a trend (H.R. 1.57, *n* = 171, *p* = 0.0687) towards poorer outcome in the BL-1 subtype (Table 1) [43]. The general features of the BL-2 subtype tumors include higher expression of myoepithelial markers and certain growth factor receptors, such as EGFR and MET [5]. Up to one third of TNBCs display evidence of myoepithelial cell differentiation [44,45]. That Annexin A1 should be highest in this subtype is consistent with strong Annexin A1 expression by both normal myoepithelial cells and those surrounding benign mammary lesions such as carcinoma in situ [27,28]. Despite several reports showing that Annexin A1 potentiates breast cancer cell invasion and/or metastasis [27,33,34], Annexin A1 was not associated with distant metastasis-free survival (DMFS) in basal-like tumors or in any of the four TNBC molecular subtypes, although sample sizes were smaller than for relapse free survival (RFS).

To further explore the relationship between Annexin A1 and metastatic potential, the levels of Annexin A1 and its key cell surface receptors, FPR1 and FPR2, were obtained from gene expression profiling of experimental human and mouse breast tumors with or without spontaneous metastatic capacity in vivo [46,47]. When primary tumor cells isolated from three different metastatic MDA-MB-231-derived xenograft models were compared with parental non-metastatic MDA-MB-231 primary tumor cells [47], no differences in Annexin A1 mRNA expression were found (Figure S2A). FPR1 was expressed at lower levels in the three metastatic daughter lines (Figure S1B), while expression of FPR2 was not detected. In a panel of mouse mammary tumor models, no differences in Annexin A1 mRNA expression were seen between non-metastatic (67NR, EO771) and spontaneously metastatic (4T1.2, EO771.LMB, EMT6.5) whole tumors (Figure S2C) [46]. Again, expression of mouse Fpr1 was lower in the group of metastatic (4T1.2, EO771.LMB, EMT6.5) compared to the non-metastatic (67NR, EO771) tumors, and Fpr1 mRNA levels were significantly lower in metastatic 4T1.2 tumors compared with its isogenic non-metastatic counterpart, 67NR (Figure S2D).

### 2.2. Evaluation of Annexin A1 Function in Xenograft Mouse Models of Breast Cancer

To functionally assess the putative role of Annexin A1 in regulating breast cancer progression and metastasis, human TNBC-derived cell lines with high Annexin A1 expression were allocated to one of the four TNBC molecular subtypes according to their gene expression profile [5]. Since Annexin A1 expression was both highest and associated with relapse-free survival in the BL-2 TNBC subtype (Figure 1C, Table 1), cell lines with high Annexin A1 levels that corresponded to BL-2 tumors were sought. Both the SUM149 TNBC cell line and its corresponding SUM149 orthotopic xenograft were previously assigned to the BL-2 subtype [5,48] and selected for investigation. To study Annexin A1 effects on spontaneous metastasis, a highly metastatic variant of MDA-MB-231 designated MDA-MB-231_HM.LNm5 (MDA-MB-231_HM) was chosen [47,49,50]. This model enables straightforward assessment of both lymphatic and hematogenous metastasis through fluorescence microscopy and genomic PCR [47]. Parental MDA-MB-231 cells were assigned to the mesenchymal (M) molecular subtype [5,6].

Endogenous Annexin A1 levels were suppressed using RNA interference and the phenotype of the stable transfectants was then evaluated in vitro and in vivo. Three independent MDA-MB-231_HM lines were generated with stable knockdown of Annexin A1 mRNA and protein (Figure S3A,B), and effects on orthotopic primary tumor growth and spontaneous metastasis were assessed. Two Annexin A1 depleted lines grew at the same rate as control tumors (shANXA1_2, shANXA1_4), while the third knockdown line (shANXA1_1) grew slightly faster than controls (Figure S3C,E). There was no difference in the mass of the resected primary tumors among the groups, and reduced Annexin A1 mRNA levels were maintained in the excised primary tumors (Figure S3D). To gauge lymphatic dissemination, ipsilateral axillary lymph nodes were assessed for the presence of sentinel lymph node (SLN) metastasis. There was no difference in either the proportion of mice bearing SLN metastases (chi-squared *p* > 0.05), nor in the mass of the SLN metastases present among the four groups (Figure S3F). Finally, metastasis to lung was assessed by genomic qPCR. The Annexin A1 knockdown lines showed an overall increase in lung metastatic burden compared to the control line (ANOVA *p* < 0.05), though no significant difference was found in the extent of lung metastasis between any individual Annexin-A1-depleted line and the non-targeting control line (Figure S3G). Collectively, these results indicated that Annexin A1 expression levels influence neither the primary tumor growth rate nor spontaneous metastasis in MDA-MB-231_HM xenografts. These findings contrast with the report by Maschler et al., which showed that enforced overexpression of Annexin A1 in MDA-MB-231 cells reversed EMT and produced reduced metastasis to lung when injected into the tail vein [30]. However, spontaneous metastasis of MDA-MB-231 tumors was not examined in this study, and ectopic overexpression of proteins with the potential to oligomerize (such as Annexin A1) in a cell line with high endogenous levels may produce artifacts [51].

**Figure 2:**
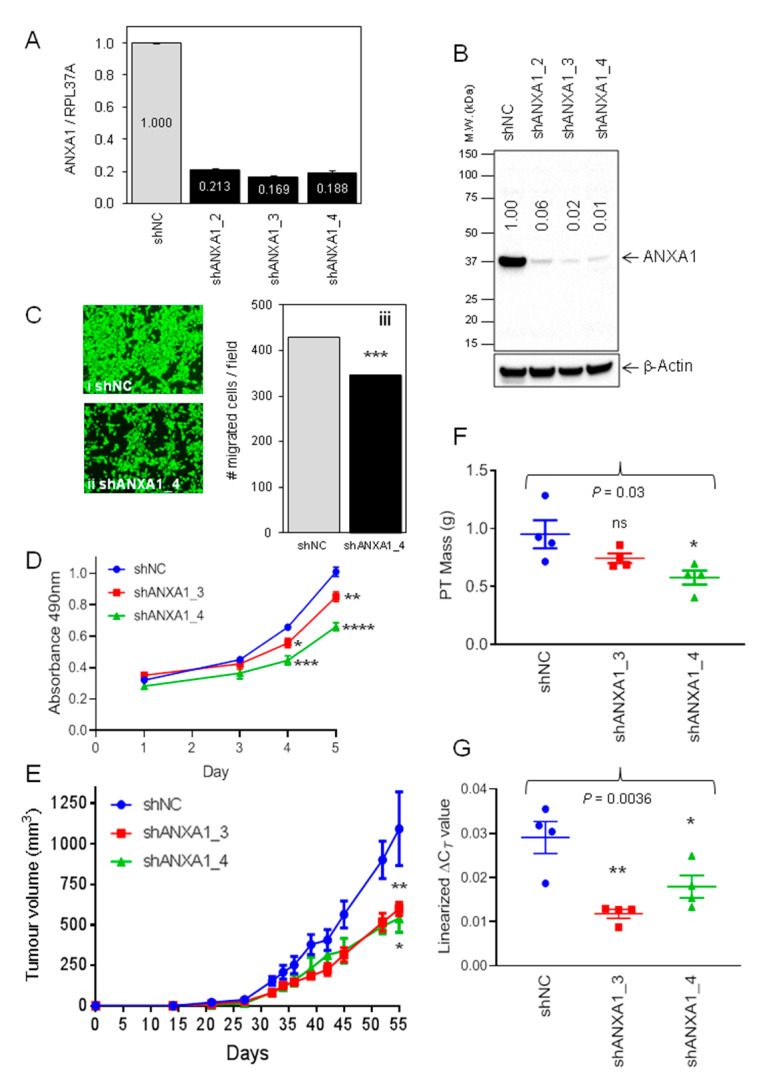
Annexin A1 depletion in SUM149 cells reduces cell motility and primary tumor growth rates in vivo (**A**) TaqMan qRT-PCR analysis of Annexin A1 mRNA levels in SUM149scidLuc2iresCherry (SUM149) cells stably expressing either control (shNC) or Annexin-A1-targeted (shAnxa1_2, shAnxa1_3, shAnxa1_4) shRNAs. Annexin A1 expression was normalized to RPL37A levels and set to 1 in shNC. Mean ± SD (*n* = 3). (**B**) Western blot analysis of Annexin A1 protein levels in the indicated cell lines. Expression was quantified by normalization to β-actin protein levels. (**C**) Transwell migration assays were conducted for 16 h in vitro followed by membrane staining with calcein^AM^. Representative images of SUM149_shNC (**i**) and SUM149_shAnxa1_4 (**ii**), membranes are shown. Magnification (×100). (**iii**) Average number of migrated cells per field. Mean ± SEM (*n* = 3). ***, *p* < 0.001. (**D**) Growth rates in 2D culture in vitro were measured for the indicated SUM149-derived stable transfectants over 5 days. Mean ± SEM (*n* = 5). The absorbance of each of the two SUM149_shANXA1 cell lines was lower than that for control SUM149_shNC cells at days 4 and 5 (Student’s *t*-test). *, *p* <0.05. **, *p* < 0.01. ***, *p* < 0.001. ****, *p* < 0.0001. (**E**) Growth rates of the indicated SUM149-derived orthotopic xenografts. Primary tumor growth rates were assessed over time by calculating tumor volume (mm^3^) from electronic caliper measurements. Mean tumor volume ± SEM (*n* = 4). Growth rates were compared using mixed effects linear regression modelling. SUM149_shNC v SUM149_shANXA1_3 (**, *p* = 0.01). SUM149_shNC v SUM149_shANXA1_4 (*, *p* = 0.03). (**F**) Mass of dissected primary tumors on day 55. Mean mass (g) ± SEM (*n* = 4 per line). One-way ANOVA *p* = 0.03. * *p* < 0.05 v SUM149_shNC control group (Dunnett’s multiple comparisons test) ns, not significant. (**G**) TaqMan qRT-PCR analysis of Annexin A1 mRNA expression in dissected SUM149-derived whole primary tumors. Annexin A1 levels were normalized to RPL37A expression. The linearized ΔC*_T_* value (Annexin A1—RPL37A) was plotted. Mean ± SEM (*n* = 3 tumors per group). One-way ANOVA *p* = 0.0036. SUM149_shNC v SUM149_shANXA1_3, ** *p* < 0.01. SUM149_shNC v SUM149_shANXA1_4, * *p* < 0.05 (Dunnett’s multiple comparisons test).

Using the SUM149 cell line to represent the BL-2 subtype of TNBC [5], three independent lines with stable suppression of Annexin A1 were generated (Figure 2A,B). The two lines with the greatest Annexin A1 knockdown (shANXA1_3 and shANXA1_4) were selected for evaluation. Immunofluorescence analysis of control SUM149 cells showed an even distribution of endogenous Annexin A1 across the cytoplasm and nucleoplasm, and the expected accumulation at the plasma membrane (Figure S4A). Reduced Annexin A1 staining was observed in the two Annexin-A1-suppressed lines (Figure S4B,C). Suppression of Annexin A1 attenuated the vigorous motility of SUM149 cells in vitro (Figure 2C), similar to other previously reported TNBC lines [33,34], and also resulted in a reduced in vitro proliferation rate (Figure 2D). The growth rates of orthotopic SUM149 xenografts were then evaluated over a 55-day period. SUM149_shANXA1_3 and SUM149_shANXA1_4 tumors developed at similar rates to each other, but more slowly than control SUM149_shNC tumors (Figure 2E) and yielded smaller tumors (Figure 2F). Importantly, we confirmed that reduced Annexin A1 mRNA expression was maintained in both Annexin A1 knockdown tumor lines on day 55 (Figure 2G). Annexin A1 protein distribution across primary SUM149_shNC tumors was heterogeneous, and staining intensity was less in both SUM149_shANXA1 tumor lines (Figure S5A). Both the control and Annexin-A1-suppressed-SUM149 tumors displayed mixed epithelial/mesenchymal phenotypes with all tumors featuring simultaneous expression of the prototypic epithelial markers, cytokeratin 8–18 (Figure S5B) and E-cadherin (Figure S5C), as well as of the mesenchymal marker vimentin (Figure S5D). This agrees with a prior report of epithelial-mesenchymal plasticity in this cell line [12]. No differences were observed in micro vessel density among the different SUM149-derived lines as determined by staining for mouse CD34 (Figure S5E).

To assess the effects of Annexin A1 knockdown on tumor behavior in immune competent mice, we screened a panel of mouse mammary tumor lines and NMuMG immortalized mammary epithelial cells (Figure S6A) for Annexin A1 mRNA and protein expression. Consistently high expression was found (Figure S6B,C), regardless of the model type (genetically engineered or spontaneously arising) or mouse strain (Figure S6A). Since BL-2 tumors were enriched for markers of normal mammary myoepithelial cells [5], we surveyed mouse mammary tumor lines for expression of alpha smooth muscle actin (αSma), a key marker of myoepithelial cells in human and mouse [52,53]. The PyMT cell line expressed the highest levels of αSma mRNA (Figure S6D). The PyMT line was isolated from a single mammary tumor that arose in a C57BL/6 MMTV-PyMT (mouse mammary tumor virus-polyoma middle T tumor-antigen) transgenic mouse [54]. PyMT-driven mouse mammary tumors are heterogeneous in their histology and gene expression profiles [55,56], and progress to hormone receptor independence over time [57].

**Figure 3:**
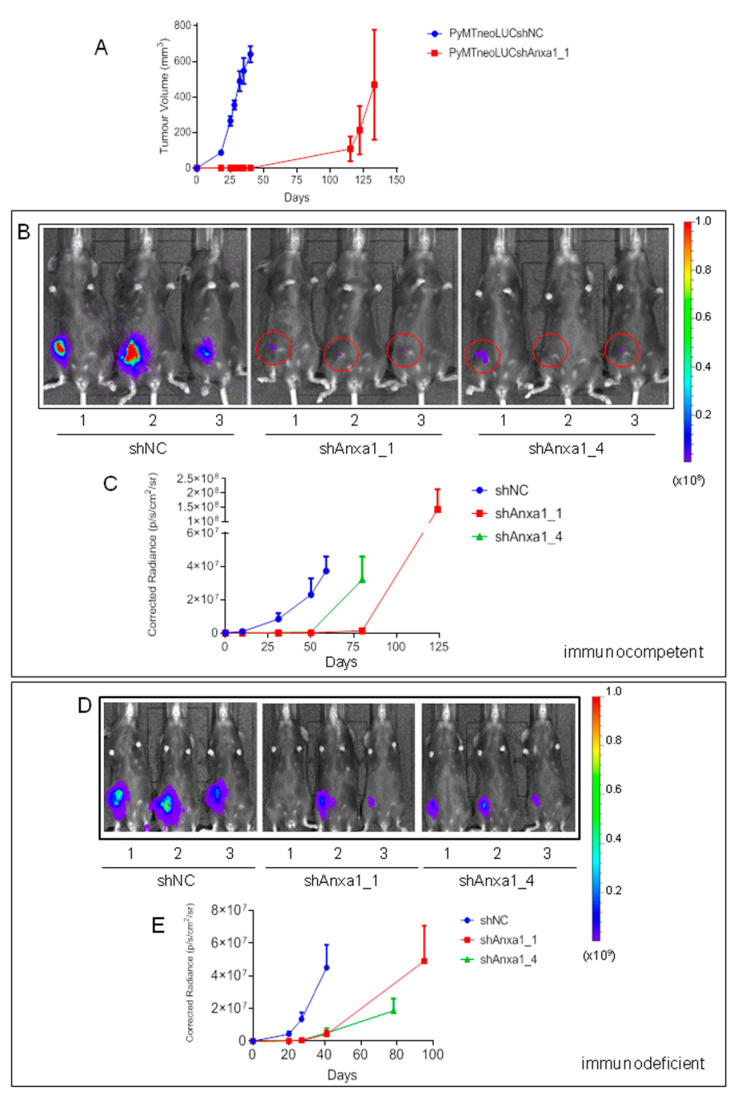
Tumor formation is delayed in both immuno-competent and immuno-deficient C57BL/6 mice with orthotopic transplantation of Annexin-A1-depleted PyMTneoLUC cells. (**A**) Growth rates of orthotopic control PyMTneoLUC_shNC (*n* = 7) and PyMTneoLUC_shAnxa1_1 (*n* = 5) in C57BL/6 mice were determined by measurement with electronic calipers. Control mice were culled on day 40 and PyMTneoLUC_shAnxa1_1 tumor-bearing mice were culled on day 133. Mean ± SEM. (**B**) Bioluminescent images of C57BL/6 mice bearing the indicated PyMTneoLUC stable transfectants (*n* = 3 per group from a total 4 mice per group) on day 31 after inoculation. The color bar on the right indicates radiance (p/sec/cm^2^/sr). Minimum 8 × 10^5^, maximum 1 × 10^8^. (**C**) Quantification of bioluminescent signals in C57BL/6 mice-bearing PyMTneoLUC_shNC (days 10, 31, 50, 59), PyMTneoLUC_shAnxa1_1 (days 10, 31, 50, 80, 124), or PyMTneoLUC_shAnxa1_4 (days 10, 31, 50, 80) tumors. The background corrected radiance is plotted versus time. Mean ± SEM. Three mice per group were assessed at each time point (*n* = 4 total per group). (**D**) Bioluminescent images (day 41) of C57BL/6NTac;B10(Cg)-Rag^2tm1Fwa^Il2rg^tm1Wjl^ immuno-deficient mice bearing representative control PyMTneoLUC_shNC (*n* = 3), PyMTneoLUC_shAnxa1_1 (*n* = 3), or PyMTneoLUC_shAnxa1_4 (*n* = 3) orthotopic tumors (*n* = 5 per group total). The color bar on the right indicates radiance (p/sec/cm^2^/sr). Minimum 2 × 10^6^, maximum 1 × 10^9^. (**E**) Quantification of bioluminescent signals in tumor-bearing C57BL/6NTac; B10(Cg)-Rag^2tm1Fwa^Il2rg^tm1Wjl^ mice (*n* = 5 per group). PyMTneoLUC_shNC (days 20, 27, 41), PyMTneoLUC_shAnxa1_1 (days 20, 27, 41, 95), PyMTneoLUC_shAnxa1_4 (days 20, 27, 41, 78). The radiance (background corrected) is plotted versus time. Mean ± SEM.

ERα- and erb-b2-negative PyMT cells (Figure S7) were transduced with the Firefly luciferase reporter gene to enable in vivo bioluminescence imaging (BLI), thereby yielding PyMTneoLUC (see Materials and Methods). Two stable Annexin A1 depleted lines were then generated in each of the PyMTneoLUC and EO771.LMB C57BL/6 cell lines (the EO771.LMB murine TNBC line is described in detail elsewhere) [46], using lentiviral-mediated delivery of Annexin A1-specific shRNA (Figure S8A,B). Control PyMTneoLUC_shNC cells grew rapidly in the orthotopic site (Figure 3A). Intriguingly, the Annexin-A1-depleted PyMTneoLUC_shAnxa1_1 line failed to form palpable tumors for up to 40 days after inoculation. However, 4 out of 5 mice implanted with PyMTneoLUC_shAnxa1_1 cells developed palpable tumors by day 115, and all five mice bore tumors at day 133 (Figure 3A), though the ultimate tumor sizes were variable (Figure S9A–C). The latent tumor formation in Annexin-A1-depleted cells suggested that a small number of cells were able to survive in a dormant state for approximately 100 days, before beginning to proliferate. To examine the in vivo behavior of these cells more closely, bioluminescence imaging (BLI) was conducted on mice bearing the PyMTneoLUC transfectants (Figure 3B,C). Both of the Annexin-A1-depleted tumor lines (shAnxa1_1 and shAnxa1_4) displayed weak BLI signals at days 10, 31 and 50 (Figure 3B, Figure S10), despite being non-palpable up to at least day 59 after inoculation, whereas control tumors showed robust BLI signals (Figure 3B). This demonstrated that small numbers of dormant, but viable, Annexin-A1-suppressed PyMTneoLUC cells were both present and detectable from early time points by optical imaging. Expansion of PyMTneoLUC_shAnxa1_4 tumors occurred after day 50, while growth of the PyMTneoLUC_shAnxa1_1 tumors was initiated after day 80 (Figure 3C, Figure S10B). The relative latency of tumor formation for the two Annexin-A1-suppressed lines was proportional to levels of residual endogenous Annexin A1 expression (Figure S8A), indicating that endogenous levels of Annexin A1 directly controlled the tumor-forming ability of PyMT cells.

To investigate the function of Annexin A1 on the cancer microenvironment, we evaluated the growth of PyMTneoLUC-derived cell lines in immuno-deficient C57BL/6NTac; B10(Cg)-Rag^2tm1Fwa^Il2rg^tm1Wjl^ mice. This strain lacks the genes encoding the Rag2 DNA recombinase and IL-2 receptor gamma chain and consequently lacks mature T cells, B cells and natural killer (NK) cells [58]. Control PyMTneoLUC_shNC tumors grew at similar rates in immuno-deficient and immuno-competent hosts (Figure 3C,E). Annexin-A1-depleted tumors again arose with much increased latency compared with control tumors (Figure 3D,E, Figure S11A–C), with weakly BLI-positive non-palpable tumors observed at days 20, 27, and 41 after inoculation (Figure S11A). PyMTneoLUC_shNC tumor-bearing mice were culled at day 41 with an average tumor weight of 0.6g (Figure S11B), whereas the Annexin A1 knockdown tumors were only barely palpable at the same stage (Figure 3D,E, Figure S11A). These results suggested that lymphocytes are not involved in regulating the unique dormant phenotype of PyMTneoLUC_shAnxaA1 tumors, which is likely to be a cell autonomous phenomenon.

The regulation of tumor formation by Annexin A1 was also tested using the EO771.LMB transplantable TNBC allograft model (Figure S8B, Figure S12A), described previously [46]. When implanted into Anxa1 null syngeneic mice, murine Lewis lung carcinoma (LLC) cells were shown to have a reduced growth rate and spontaneous metastasis to lung relative to wild-type mice [59]. Therefore, the growth rates of control and Annexin-A1-depleted EO771.LMB orthotopic allografts were assessed in wild-type mice as well as in syngeneic C57BL/6 mice null for either Anxa1, or its receptor on regulatory T cells, Fpr2 [26]. Stable Annexin A1 knockdown had no effect on the initiation or growth rates of EO771.LMB tumors in wild-type (Figure S12B,E), Anxa1 null (Figure S12C), or Fpr2 null (Figure S12D) mice, and had no effect on final tumor mass (Figure S12F). In addition, no differences were recorded when the growth rates of control EO771.LMB_shNC tumors were compared across wild-type, Anxa1^−/−^, and Fpr2^−/−^ mice (Figure S12G). These data suggested that the requirement for high level tumor cell expression of Annexin A1 for efficient tumor initiation in vivo might be specific to the PyMT line.

**Figure 4:**
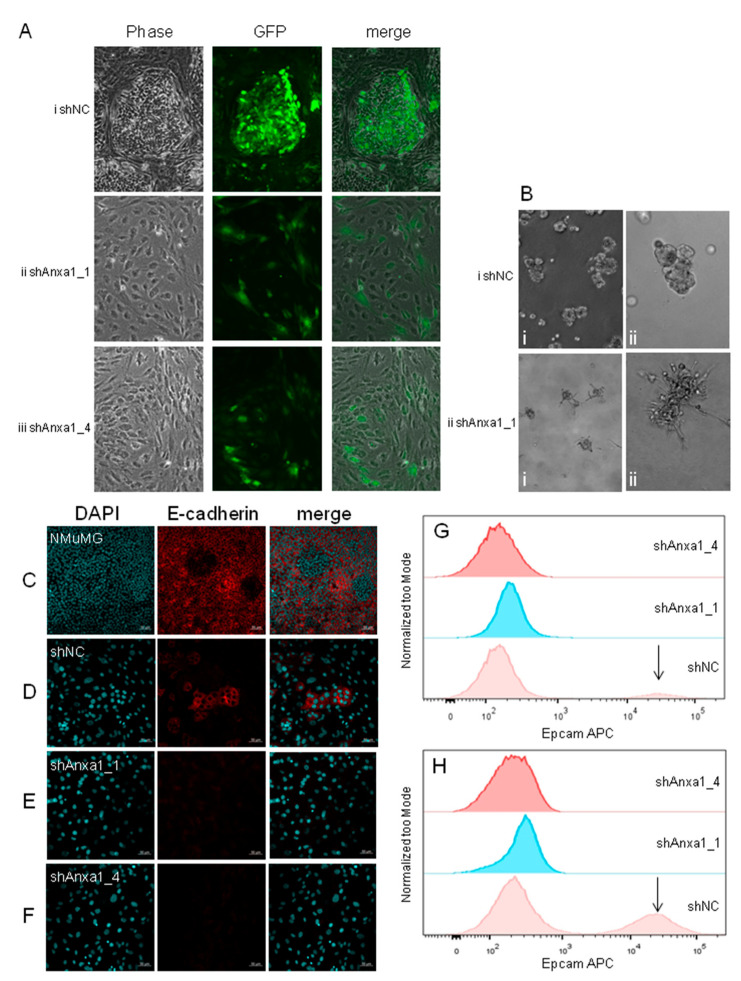
Phenotypes of Annexin-A1-depleted PyMTneoLUC cells in vitro. (**A**) Phase contrast (left panels), fluorescent (eGFP, middle panels), and merged (right panels) images of the indicated PyMTneoLUC stable transfectants cultured for 4 days post confluence. ×100 magnification. (**B**) Morphology of control PyMTneoLUC_shNC and PyMTneoLUCshAnxa1_1 cells in 3D culture. (**i**) ×100 magnification. (**ii**) ×200 magnification. (**C**) NMuMG, (**D**) PyMTneoLUC_shNC, (**E**) PyMTneoLUC_shAnxa1_1, and (**F**) PyMTneoLUC_shAnxa1_4 cells were grown 4 days post confluence and stained for nuclei (DAPI, left panels), E-cadherin (red, middle panels), and the images merged (right panels). Scale bars are 50 μm. (**G**,**H**) The indicated PyMTneoLUC stable transfectants were cultured in vitro and cells collected at confluence (**G**), or 4 days post-confluence (**H**), for analysis of cell surface Epcam expression by flow cytometry. The Epcam^HI^ population in PyMTneoLUC_shNC cells is indicated with an arrow.

To gain insight into the reason(s) for the delayed growth of Annexin A1 knockdown PyMTneoLUC tumors in vivo, a suite of cellular characteristics was examined in vitro, including proliferation, cell morphology, and expression of genes relevant to the biology of breast cancer and cancer stem cells. Annexin-A1-depleted PyMTneoLUC cells had an increased growth rate in culture compared to controls (Figure S13), suggesting that a difference in general proliferative ability is unlikely to explain the delayed growth of Annexin A1 knockdown cells in vivo. However, extensive changes to cell morphology and subcellular structures were also observed. When cultured post-confluence, control PyMT cells form heterogeneous structures consisting of clusters of differentiated epithelial cells, resembling filled epithelial acini, surrounded by mesenchymal- or myoepithelial-appearing cells (Figure 4Ai). The acini expressed cell surface E-cadherin, a critical component of epithelial *adherens* junctions (Figure 4D). Notably, E-cadherin distribution was punctate and cytoplasmic in confluent or pre-confluent cultures of control PyMT cells (Figure S14A). Similar to the pattern of expression of E-cadherin, the proportion of control cells positive for the epithelial marker Epcam increased in post-confluent cells (Figure 4H) relative to confluent cells (Figure 4G). Conversely, Annexin-A1-suppressed PyMTneoLUC cells failed to form E-cadherin-positive epithelial acini post confluence (Figure 4E,F) and showed reduced cytoplasmic E-cadherin in confluent and pre-confluent cultures (Figure S14B,C). In fact, PyMTneoLUC_shAnxa1 cultures appeared to be solely comprised of large mesenchymal- or myoepithelial-appearing cells (Figure 4A(ii,iii)). Annexin-A1-suppressed cells were indeed larger in size than control PyMTneoLUC cells as determined by flow cytometry (Figure S14D). The larger cytoplasm in the Annexin-A1-suppressed PyMTneoLUC lines was also associated with more extensive microtubule networks and prominent perinuclear microtubule organizing centers (Figure S15A–C).

The apparent heterogeneous appearance of PyMT-derived cells in confluent cultures is reminiscent of the LM38 BALB/c mammary tumor model, which was derived from a tumor that arose spontaneously in a pregnant mouse [60]. Low passage number LM38 cells (LM38-LP) comprise a mixture of differentiated luminal epithelial cells and spindle-shaped myoepithelial cells that can be isolated and grown independently of each other in vitro and in vivo. This observation demonstrates that the cellular heterogeneity of mouse mammary tumor cells is not restricted to those whose transgenes are driven by the MMTV promoter, nor those that are induced by the PyMT viral oncogene. In three-dimensional cultures, control PyMTneoLUC cells formed compact spheroids with smooth edges. However, PyMTneoLUC_shAnxa1_1 cells grew as loose clusters of stellate cells featuring multiple protrusions into the basement membrane matrix (Figure 4B), a key characteristic of carcinoma cells that have undergone EMT [61,62].

Taken together, these data indicated that the Annexin-A1-depleted cells may have become locked into an altered mesenchymal or partial myoepithelial state [63] and lacked the requisite plasticity to undergo in vitro morphogenesis into filled epithelial acini post confluence. A requirement for Annexin A1 in the proper morphogenesis of prostatic epithelial acini in vitro was suggested previously [64]. Notably, EO771.LMB cells will not grow to confluence and thus are unable form differentiated epithelial structures in vitro. Annexin A1 suppression did not appear to grossly affect cellular phenotype of EO771.LMB cells (Figure S12A).

### 2.3. Suppression of Annexin A1 in PyMTneoLUC Cells Results in Epithelial-to-Mesenchymal Transition and Loss of a Putative Tumor-Initiating Cell Population

**Figure 5:**
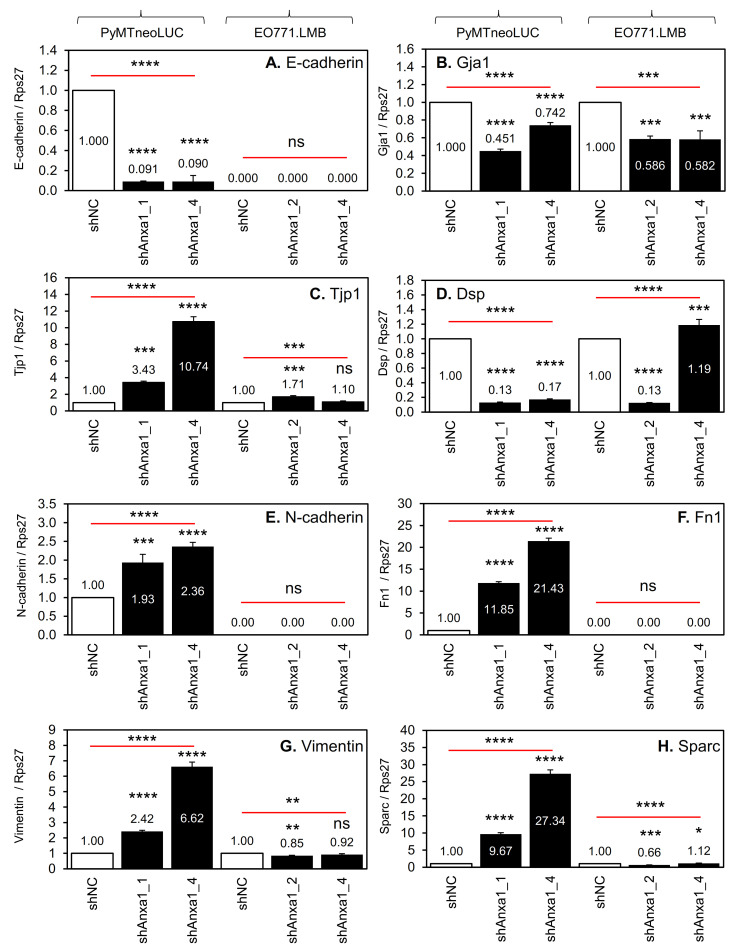
Expression of epithelial and mesenchymal markers in mouse mammary tumor cell lines harboring Annexin A1 knockdown qRT-PCR analysis was conducted on the indicated cell lines as described in the Materials and Methods. Gene expression was normalized to Rps27 levels and mRNA levels of genes of interest was set to 1 in control PyMTneoLUC_shNC and EO771.LMB_shNC cell lines. (**A**–**D**) Epithelial markers. (**E**–**H**) Mesenchymal markers. (**A**) E-cadherin. (**B**) Gja1. (**C**) Tjp1. (**D**) Dsp. (**E**) N-cadherin. (**F**) Fibronectin (Fn). (**G**) Vimentin. (**H**) Sparc/osteonectin. Mean ± SD (*n* = 3). Data were analyzed using one-way ANOVA followed by Dunnett’s post hoc test for multiple comparisons to compare to control shNC cells *, *p* < 0.05. **, *p* < 0.01. ***, *p* < 0.001. ****, *p* < 0.0001. ns (not significant).

To further explore changes of cellular state in the PyMT-derived cells, the expression levels of additional epithelial and mesenchymal markers alongside markers of basal/myoepithelial and luminal mammary epithelial cells were examined in control and Annexin A1 knockdown, cell lines PyMTneoLUC, EO771.LMB, and SUM149. E-cadherin mRNA and protein expression was almost completely lost in Annexin-A1-depleted PyMTneoLUC cells (Figure 5A, Figures S16Ai and S14B,C). E-cadherin protein was slightly downregulated in the three SUM149_shANXA1 lines compared to controls (Figure S16Aiii) but was not expressed by EO771.LMB cells (Figure 5A, Figure S16Aii). Protein and mRNA levels of vimentin, an intermediate filament component in mesenchymal cells, were increased in both PyMTneoLUC_shAnxa1 lines compared with control cells (Figure 5G, Figure S16Bi), whereas no upregulation was found in EO771.LMB or SUM149 cells harboring Annexin A1 knockdown (Figure 5G, Figure S16B(ii,iii)). Both PyMTneoLUC_shAnxa1 and EO771.LMB_shANXA1 cell lines showed small reductions in mRNA levels of the gap junction component connexin 43 (Gja1, Figure 5B), whereas only the PyMTneoLUC_shAnxa1 lines showed increased expression of the tight junction component, zonula occludens-1 (Tjp1, Figure 5C), and decreased expression of the desmosomal component, desmoplakin (Dsp, Figure 5D). With respect to other mesenchymal markers, both Annexin-A1-depleted PyMTneoLUC lines showed markedly increased expression of both fibronectin (Figure 5F) and Sparc/osteonectin (Figure 5H), and a smaller increase in N-cadherin levels (Figure 5E). EO771.LMB_shAnxa1 cell lines displayed no change in expression of these three mesenchymal markers (Figure 5). In an inverse pattern to that observed in mouse PyMTneoLUC cells, Annexin-A1-depleted human SUM149 cells displayed a moderate decrease in expression of the mesenchymal markers N-cadherin (CDH2, Figure S17B), fibronectin (Figure S17C), tenascin C (Figure S17D), and caveolin 1 (Figure S17E). Levels of E-cadherin (CDH1, Figure S17A) and cadherin 11 (CDH11, Figure S17F) were unchanged. The magnitude of the reduction in expression of mesenchymal markers correlated with endogenous Annexin A1 protein levels in the three SUM149_shANXA1 lines (Figure 2B). Together, the gene expression data and morphological findings indicated that reduced Annexin A1 expression lead to epithelial to mesenchymal transition in PyMTneoLUC cells but not in EO771.LMB or SUM149 cells.

**Figure 6:**
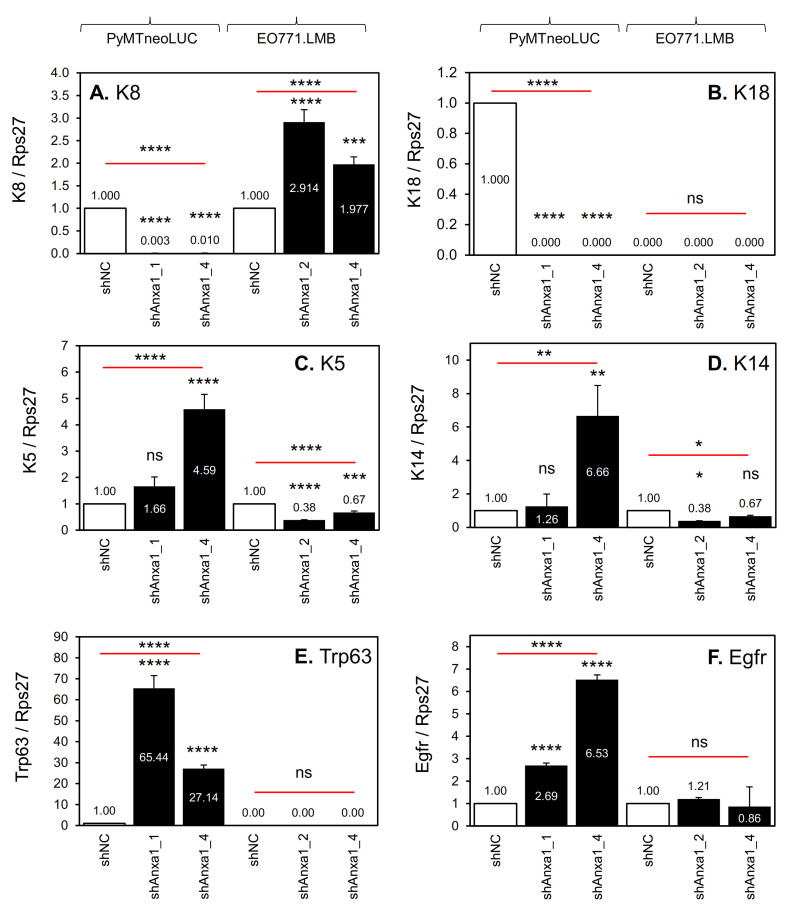
Expression of basal and luminal mammary epithelial cell markers in mouse mammary tumor cell lines harboring Annexin A1 knockdown qRT-PCR analysis was conducted on the indicated cell lines as described in the Materials and Methods. Gene expression was normalized to Rps27 levels and set to 1 in control PyMTneoLUC_shNC and EO771.LMB_shNC cell lines. (**A**) Luminal cytokeratin 8 (K8). (**B**) Luminal cytokeratin 18 (K18). (**C**) Basal cytokeratin 5 (K5). (**D**) Basal cytokeratin 14 (K14). (**E**) Basal Trp63. (**F**) Basal Egfr. Mean ± SD (*n* = 3). Data were analyzed using one-way ANOVA followed by Dunnett’s post hoc test for multiple comparisons to compare to control shNC cells. *, *p* < 0.05. **, *p* < 0.01. ***, *p* < 0.001. ****, *p* < 0.0001. ns (not significant).

With regard to markers of mammary epithelial cell lineage, both of the PyMTneoLUC_shAnxa1 lines had almost completely lost expression of luminal cytokeratin 8 (K8) and its binding partner cytokeratin 18 (K18) (Figure 6A,B) [65,66], and showed increased expression of both basal/myoepithelial cytokeratin 5 (K5, Figure 6C) and its binding partner keratin 14 (K14, Figure 6D) [65,66]. These cell lines also featured concomitant upregulation of the basal/myoepithelial epithelial markers, Trp63 and Egfr (Figure 6E,F) [65,66]. Conversely, there was no loss of luminal epithelial markers (Figure 6A,B), nor gain of basal epithelial markers (Figure 6C–F) by EO771.LMB_shAnxa1 cell lines. The three SUM149_shANXA1 cell lines showed a small reduction in EGFR mRNA expression (Figure S17G). While N-cadherin, fibronectin, vimentin, and Sparc/osteonectin are classic markers of mesenchymal cells, each of them are also more highly expressed in mouse mammary myoepithelial cells than in either normal luminal ERα-positive or luminal ERα-negative lineages [65].

**Figure 7:**
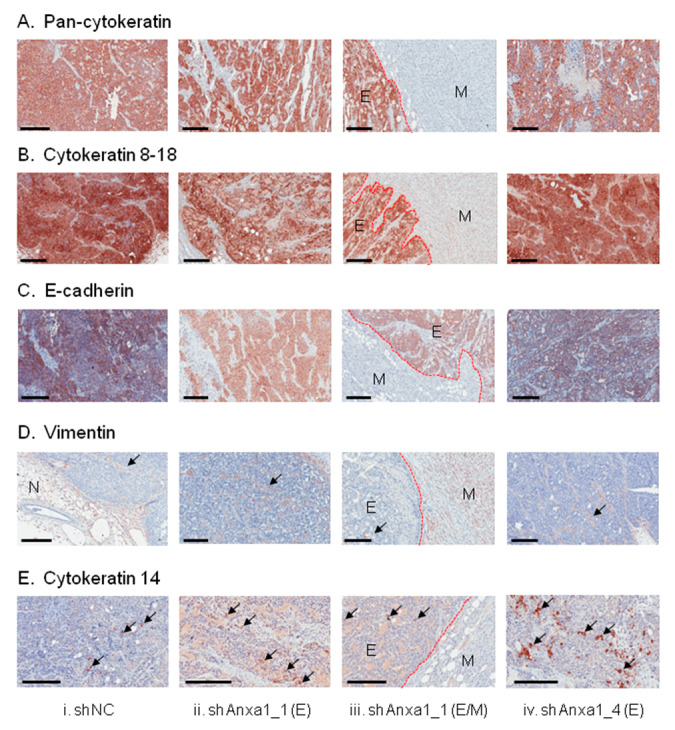
Immunohistochemical analysis of control PyMTneoLUC_shNC, PyMTneoLUC_shAnxa1_1, and PyMTneoLUC_shAnxa1_4 mammary tumor allografts. Tumors formed in C57BL/6NTac;B10(Cg)-Rag^2tm1Fwa^Il2rg^tm1Wjl^ immuno-deficient mice by the indicated PyMTneoLUC-derived stable transfectants were stained for (**A**) pan-cytokeratin, (**B**) cytokeratin 8–18 (**C**) E-cadherin, (**D**) vimentin, (**E**) cytokeratin 14. Representative epithelial (**E**) zones from PyMTneoLUC_shAnxa1_1 (**ii**) and PyMTneoLUC_shAnxa1_4 (**iv**) tumors including the border region are shown, demarcating the epithelial (**E**) and mesenchymal (M) zones of PyMTneoLUC_shAnxa1_1 tumors (**iii**). (**D**). Arrows indicate vimentin-positive stromal cells. (**E**) Arrows indicate examples of small clusters of cytokeratin-14-positive cells. N, normal mammary gland. Scale bars represent 200 μm.

We next examined expression of epithelial, mesenchymal, basal, and luminal markers by immunohistochemistry in primary control PyMTneoLUC_shNC allografts, and also in those PyMTshAnxa1_1 and PyMTshAnxa1_4 mammary tumors that arose after a long latency. While all neoplasms appeared similar macroscopically (Figure S10B,C), gross differences in tumor architecture were apparent microscopically. Control PyMTneoLUC_shNC tumors displayed homogeneous staining for pan-cytokeratin (Figure 7Ai) and luminal cytokeratins 8–18 (Figure 7Bi) and widespread expression of E-cadherin (Figure 7Ci). However, all delayed PyMTneoLUC_shAnxa1_1 (Figure 7Aiii–Eiii, Figure S18) and PyMTneoLUC_shAnxa1_4 tumors contained large regions of spindle-shaped tumor cells, in addition to zones of epithelial neoplastic cells, regardless of whether the cells were implanted into immuno-competent or immuno-deficient mice (Figure 7Aiii–Eiii, Figure S18). These mesenchymal zones were contiguous, usually separated from the epithelial region by a well-defined border, and often comprised over 50% of the total tumor area (Figure S18). The spindle-shaped tumor cells in the mesenchymal zones were negative for pan-cytokeratin (Figure 7Aiii, Figure S18A), cytokeratins 8–18 (Figure 7Biii, Figure S18B), and E-cadherin (Figure 7Ciii, Figure S18C), whereas the epithelial zones of Annexin-A1-depleted tumors stained positive for all three markers (Figure 7A(ii–iv), Figure 7B(ii–iv), Figure 7 C(ii–iv)). Despite the expression of vimentin by all three transduced PyMTneoLUC lines in culture (Figure S16Bi), only the stromal cells of primary PyMTneoLUC_shNC allografts were vimentin positive (Figure 7Di), as were stromal cells in the epithelial zones of PyMTneoLUC_shAnxa1_1 and PyMTneoLUC_shAnxa1_4 tumors (Figure 7D(ii–iv)). As anticipated, the fibroblastoid cells in the mesenchymal zones of Annexin-A1-suppressed tumors were positive for vimentin (Figure 7Diii). Finally, the basal marker cytokeratin 14 was expressed by small clusters of tumor cells in the epithelial regions of all tumors (Figure 7E(i–iv)), though increased numbers of clusters were observed in the epithelial zones of both PyMTneoLUC_shAnxa1_1 (Figure 7Eii) and PyMTneoLUC_shAnxa1_4 tumors (Figure 7Eiv). The absence of K14 positivity in the mesenchymal zone of the Annexin A1 knockdown tumors suggests that the cells in these regions are indeed more likely to be mesenchymal rather than conventional myoepithelial cells. Appropriate expression of cytokeratin 14, pan-cytokeratin, and cytokeratin 8–18 in normal mouse mammary epithelium demonstrated the specificity of the antibodies used (Figure S19).

Tumorigenic human and murine cancer cell lines can often be divided into a minor population of cells with tumor-initiating capacity and a major population that lacks tumor forming ability [67,68,69]. Therefore, we sought to ascertain whether the loss of Annexin A1 expression caused a depletion of putative tumor-initiating cells (TICs) which might explain the long latency of PyMT_shAnxa1_1 and PyMT_shAnxa1_4 tumors compared to control tumors. TICs present in many human breast cancers reside within a small population of CD44^+^/CD24^−^ cells that can be isolated by flow cytometry [67]. MMTV-Neu induced mammary tumors [70], and tumors and a cancer cell line isolated from MMTV-PyMT transgenic mice were also reported to contain a minor TIC population with a CD44^+^/CD24^−^ phenotype [71]. However, it is now generally accepted that TICs from most mouse mammary tumor models are actually positive for CD24 [72,73,74,75], as are normal mouse mammary epithelial stem cells [72,76]. The cancer stem cell compartment of both MMTV-PyMT mouse mammary tumors and the cancer cell lines derived from them harbor a CD24^+^/CD49f (α6 integrin)^+^/CD29 (α1 integrin)^+^/Sca1 (stem cell antigen-1)^low^ surface phenotype in accordance with several other genetically engineered mouse breast tumor models [73,74,77,78]. Therefore, control and Annexin-A1-depleted PyMTneoLUC cell lines were analyzed for cell surface expression of markers of TICs.

**Figure 8:**
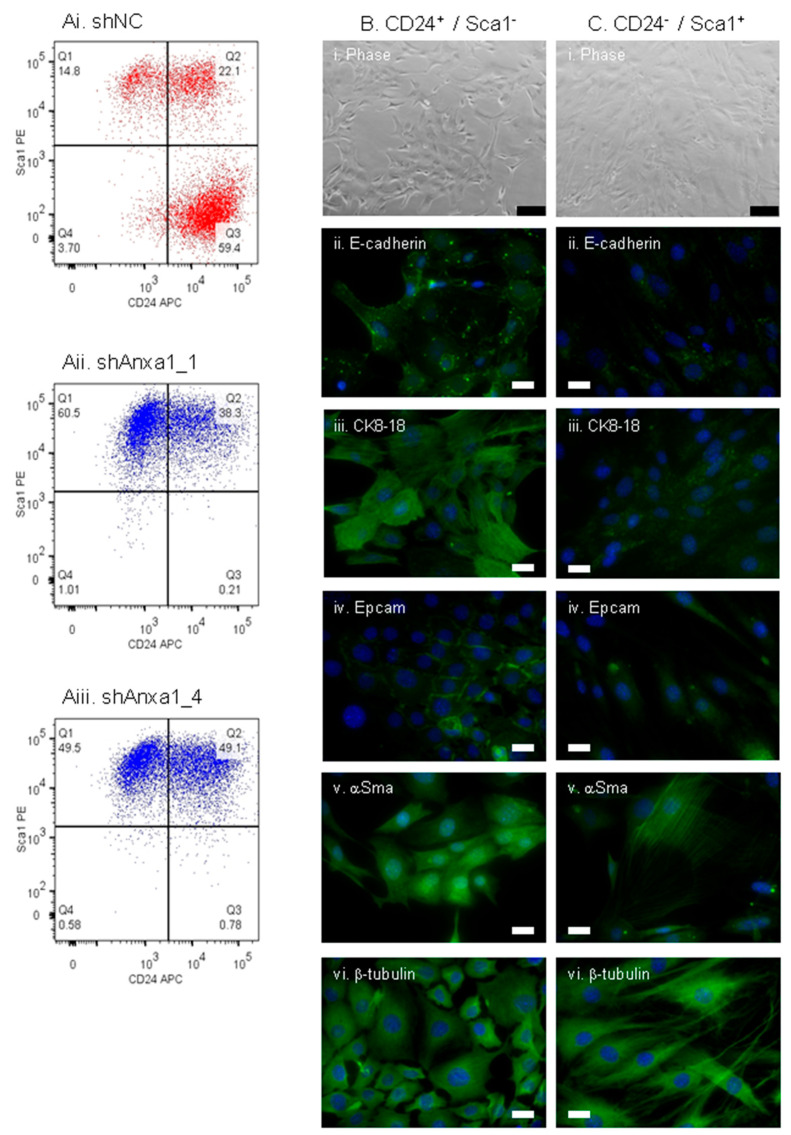
Expression of markers of tumor-initiating cells in control and Annexin-A1-depleted PyMTneoLUC cells. (**A**) (**i**) Control PyMTneoLUC_shNC, (**ii**) PyMTneoLUC_shAnxa1_1, and (**iii**) PyMTneoLUC_shAnxa1_4 cultured cell lines were analyzed by flow cytometry for Sca1 and CD24 cell surface expression. (**B**,**C**) Morphology and marker expression of sorted PyMT cells. Parental PyMT cells were sorted into CD24^+^/Sca1^−^ (**B**) and CD24^−^/Sca1^+^ (**C**) populations and cultured on collagen-coated slides for 6 days prior to assessment of morphology and marker expression by immunofluorescence. (**i**) Phase contrast microscopy. Scale bar 500 μm. (**ii**) E-cadherin. (**iii**) Cytokeratin 8–18. (**iv**) Epcam. (**v**) Alpha smooth muscle actin (αSma). (**vi**) β-tubulin. White scale bars represent 20 μm.

All PyMTneoLUC transfected cells expressed high levels of CD29 regardless of their Annexin A1 status (data not shown). However, control PyMTneoLUC_shNC cells contained three clearly demarcated populations defined by Sca1 and CD24 expression (Figure 8Ai), with the majority of cells (59%) displaying a CD24^+^/Sca1^−^ phenotype, a subpopulation of which should correspond to TICs [73,75], and minor CD24^+^/Sca1^+^ (22%) and CD24^−^/Sca1^+^ (15%) populations. Both Annexin A1 knockdown lines displayed reduced CD24 expression and concomitant up regulation of Sca1 expression such that 50% or more of PyMTneoLUC_shAnxa1 cells now exhibited a Sca1^+^/CD24^−^ phenotype (Figure 8A(ii,iii)). Notably, the two Annexin A1 knockdown lines had almost completely lost the population of CD24^+^/Sca1^−^ putative TICs (Figure 8A(ii,iii)). As a human orthologue of murine Sca1 (also known as Ly6a) has not yet been identified, expression of mesenchymal stem cell marker CD44 was also measured [79]. A similar trend was seen for CD44, whereby the majority of control PyMTneoLUC_shNC cells were CD44 negative (Figure S20A), whilst almost all the cells in the two PyMTneoLUC_shAnxa1 lines acquired CD44 expression (Figure S20B,C). The morphology and marker expression of discrete subpopulations of the PyMT cell line defined by CD24 and Sca1 expression were then evaluated. Parental PyMT cells were sorted into their prominent CD24^+^/Sca1^−^ and less abundant CD24^−^/Sca1^+^subsets (data not shown), briefly cultured separately in vitro, and then assessed prior to confluence. An epithelial morphology was seen for CD24^+^/Sca1^−^ cells (Figure 8B(i,vi)), while CD24^−^/Sca1^+^ cells displayed a spindle-shaped mesenchymal morphology (Figure 8C(i,vi)). Accordingly, CD24^+^/Sca1^−^ cells showed a filamentous staining pattern for luminal cytokeratins 8–18 and the presence of cell surface Epcam (Figure 8B(iii,iv)), while CD24^−^/Sca1^+^ cells were negative (Figure 8C(iii,iv)). The pre-confluent CD24^+^/Sca1^−^ cultures also had an increased abundance of cytoplasmic E-cadherin granules compared to CD24^−^/Sca1^+^ cells (Figure 8Bii,Cii). Unexpectedly, both the epithelial and mesenchymal populations expressed αSma (Figure 8Bv,Cv). The αSma-staining pattern occurred in parallel filaments in a small proportion of CD24^−^/Sca1^+^ cells.

Taken together, these data suggest that reduced Annexin A1 expression in PyMTneoLUC cells led to an almost complete loss of luminal epithelial CD24^+^/Sca1^−^ tumor cells that contained a TIC population, potentially explaining the reduced tumor-forming ability of the PyMTneoLUC_shAnxa1 cell lines [73]. Concomitantly, Annexin A1 knockdown caused expansion of a mesenchymal CD24^−^/Sca1^+^ population. The data indicate that the CD24^−^/Sca1^+^ population is mesenchymal rather than myoepithelial in nature, as mouse mammary myoepithelial cells are Sca1 negative [65]. Moreover, mouse myoepithelial cells are not enriched for CD44 expression [65]. Interestingly, Sell and colleagues reported that cell lines isolated from MMTV-PyMT-driven tumors are heterogeneous and contain a PyMT transgene-positive mesenchymal cell population. These cells lacked an intrinsic tumor-forming ability and were able to differentiate into adipocyte- and osteocyte-like cells in vitro, indicating that they resembled mesenchymal stem cells [75]. However, these mesenchymal cells potentiated tumor formation in vivo when mixed together with putative TICs isolated from MMTV-PyMT tumor-derived cell lines. Furthermore, a substantial number of the resulting tumors were defined as sarcomas or featured a mixed sarcoma/adenocarcinoma histology [75]. Extrapolating this to the current study, these observations suggest that the increased abundance of CD24^−^/Sca1^+^ mesenchymal stem cell-like cells in the two PyMTneoLUC_shAnxa1 cell lines was responsible for the unexpected formation of carcinosarcomas, rather than adenocarcinomas. The CD24^+^/Sca1^+^ double positive population was also increased in Annexin A1 knockdown PyMTneoLUC cells compared to controls. These might represent an intermediate cell state that PyMT cells transition through as they move from an epithelial to mesenchymal phenotype and vice versa.

## 3. Materials and Methods

### 3.1. Cell Culture

SUM149PT cells were kindly provided by Steve Ethier (University of Michigan, Arbor, MI, USA). The SUM149scid variant was obtained following ex vivo expansion of a subcutaneous xenograft of parental SUM149PT cells grown in a BALB/c^scid^ mouse. The SUM149scidLuc2Ch line was generated by transducing SUM149scid cells with the pMSCV_Luc2_mCherry amphotropic retrovirus encoding the Firefly luciferase 2 and mCherry reporter genes in a Luc2-ires-mCherry cassette [47,80]. Stably transduced cells were selected by two rounds of sorting for mCherry expression by flow cytometry (FACSDiva III, Becton Dickinson, Scoresby, Vic., Australia). SUM149PT and SUM149scid cell lines were authenticated using the GenePrint^®^ 10 System (Promega Corporation, Alexandria, NSW, Australia), at the QIMR Berghofer Medical Research Institute, Herston, Qld, Australia. Cell lines were considered authentic if >80% of measured alleles matched those of the repository sample. All cell lines were authenticated within the past three years.

The isogenic C57BL/6 spontaneous mouse mammary tumor lines (EO771 and EO771.LMB) were previously described [47,81]. AT3 and PyMT mouse mammary tumor lines were derived from mammary tumors that arose in C57BL/6 mice expressing the polyoma virus middle T antigen (PyMT) under the control of the murine mammary tumor virus (MMTV) long terminal repeat [54]. AT3 has been previously described [82]. The PyMT line was established from a MMTV-PyMT mammary tumor in our laboratory. PyMT was cultured ex vivo and treated with cis-hydroxyproline (100 μg/mL) for 7 days to remove contaminating cancer-associated fibroblasts [83]. PyMT cells were frozen at early passage, and only early passage (<P5) cells were used in experiments. NMuMG immortal mouse mammary epithelial cells were obtained from ATCC [84]. PyMTneoLUC and AT3neoLUC variants were generated by transducing cells with the pFBneoLUC ecotropic retrovirus, which encodes the firefly luciferase reporter gene upstream of an ires-neo cassette [80,85]. Transduced cells were selected for stable transgene insertion using G418 (1000 μg/mL).

Cell lines were maintained in Dulbecco’s modified Eagle medium (DMEM) (Thermo Fisher Scientific, Scoresby, Vic., Australia) supplemented with 10% (*v*/*v*) heat-inactivated fetal bovine serum (FBS, Thermo Fisher Scientific), 2 mM L-glutamine, 1% (*v*/*v*) non-essential amino acids, 5% (*v*/*v*) sodium pyruvate, penicillin (100 IU/mL) and streptomycin (100 μg/mL), except for SUM149 derivatives, whose base medium was 1:1 DMEM/Ham’s F12 (Thermo Fisher Scientific). Cells were maintained at 37 °C in 5% CO_2_ (*v*/*v*) in air and sub-cultured every 4–5 days. For 3D culture of PyMT-derived lines, cells were seeded onto a solid basement membrane gel (50% Cultrex in full medium) (Trevigen, Gaithersburg, MD, USA). All cell lines were confirmed to be free of mycoplasma contamination before use.

### 3.2. Generation of Stable Annexin A1 Knockdown Cell Lines

pGIPZ lentiviral plasmids (Dharmacon, GE Life Sciences, Lafayette, CO, USA) encoding turboGFP (tGFP), the puromycin resistance gene and a short hairpin RNA (shRNA) sequence specific for human Annexin A1 (shANXA1_1, clone ID: V2LHS_112102, targeting the open reading frame (ORF); shANXA1_2, clone ID: V3LHS_392259, targeting the ORF; shANXA1_3, clone ID: V3LHS_413324, targeting the 3′UTR; shANXA1_4, clone ID: V3LHS_413325, targeting the 3′UTR) or a scrambled non-silencing control sequence (designated shNC) was obtained from the Victorian Centre for Functional Genomics (VCFG) at the Peter MacCallum Cancer Centre [86]. pGLV1/U6/eGFP lentiviral plasmids were obtained encoding enhanced GFP (eGFP) driven by the CMV promoter and a short hairpin RNA (shRNA) sequence specific for mouse Annexin A1 driven by the U6 promoter (GenePharma, Shanghai, P.R. China). All mouse Annexin A1 shRNAs targeted the coding region. The vector names and sequences targeted are as follows, (shAnxa1_1, clone ID: LV1-Anxa1-mus-219, 5′ GCT GCC TTG CAC AAA GCT ATC 3′; shAnxa1_2, clone ID: LV1-Anxa1-mus-458, 5′ GGG ACT TGG AAC AGA TGA AGA 3′; shAnxa1_3, clone ID: LV1-Anxa1-mus-644, 5′ GGA CTT GAG TTG GAA TCA AGA 3′; shAnxa1_4, clone ID: LV1-Anxa1-mus-942, 5′ GGA ACT CGC CAT AAG GCA TTG 3′). A scrambled non-silencing negative control shRNA was also used (shNC, clone ID: LV1-Negative Control, 5′ TTC TCC GAA CGT GTC ACG T 3′).

Pseudotyped lentiviruses were prepared using a 2nd generation packaging system. Briefly, 293T cells were transiently transfected with the pCMV-deltaR8.2 packaging plasmid (a gift from Didier Trono (Addgene plasmid # 12263). Addgene, Watertown, MA, USA) and pCMV-VSV-G encoding the envelope protein from vesicular stomatitis virus (a gift from Robert Weinberg (Addgene plasmid # 8454). Addgene, Watertown, MA, USA. Target cell lines were transduced using the spin infection method [47,87]. After infection with pGIPZ lentiviruses, SUM149scidLuc2Ch (SUM149) cells were selected for stable transgene integration using puromycin (10 μg/mL), followed by one round of sorting for tGFP expression by flow cytometry (FACSARIA III). pGIPZ-transduced SUM149 cells were maintained in puromycin (5 μg/mL). The mouse mammary tumor lines, PyMTneoLUC and EO771.LMB were transduced by spin infection as above, and stable transfectants selected by four rounds of sorting for eGFP expression by flow cytometry (FACSARIA III).

### 3.3. Western Blotting

Western blotting was carried out as previously described [87], with the following modifications. Horseradish peroxidase (HRP)-conjugated secondary antibodies were used at the appropriate dilution (Agilent Technologies Pathology (Dako), Mulgrave, Vic., Australia). Proteins were visualized using enhanced chemiluminescence substrate (ECL, Pierce, Thermo Fisher Scientific, Scoresby, Vic., Australia) and a Chemi-Doc instrument (Bio-Rad Laboratories, Gladesville, NSW, Australia). β-Actin was used as an internal control antibody for loading and transfer (Sigma Aldrich, Castle Hill, NSW, Australia). Signals were quantified from high resolution TIFF files using NIH Image J software (Bethesda, MD, USA) [88].

### 3.4. Antibodies

The antibodies used for Western blotting (WB), immunofluorescence (IF), immunohistochemistry (IHC) and flow cytometry (FC) were as follows: human and mouse Annexin A1 (WB, IF), mouse mAb clone EH17a (Santa Cruz Biotechnology, Dallas, TX, USA). Human Annexin A1 (IHC), mouse mAb clone MRQ-3 (Cell Marque, Sigma Aldrich). Mouse E-cadherin (IF, IHC), mouse mAb clone E-cad/36 (BD Transduction Laboratories, BD Biosciences, Scoresby, Vic., Australia). Mouse CD24 (FACS), CD24-PE-Vio770 or CD24-APC, rat mAb clone M1/69 (Miltenyi Biotec, Macquarie Park, NSW, Australia), mouse CD44 (FACS), CD44-PE, rat mAb clone IM7.8.1 (Miltenyi Biotec), mouse Epcam/CD326 (FACS), Epcam-APC, rat mAb clone G8.8 (eBioscience, Invitrogen, Thermo Fisher Scientific, Scoresby, Vic., Australia). Mouse cytokeratin 14 (IHC), mouse mAb clone LL002 (Abcam, Melbourne, Vic, Australia). Mouse pan-cytokeratin (recognizing type II cytokeratins 1, 5, 6, and 8) (IHC), mouse mAb clone PCK-26 (Sigma-Aldrich). Mouse cytokeratins 8–18 (IHC), rabbit mAb clones EP17/EP30 (Dako, Leica Microsystems, Macquarie Park, NSW, Australia). Mouse alpha smooth muscle actin (αSma) (IF), rabbit pAb ab5694 (Abcam). Mouse Sca1 (Ly6a) (FACS), Sca1-PE, rat mAb clone D7 (Miltenyi Biotec). Mouse vimentin (IHC), rabbit pAb R28 (Cell Signaling Technology, Danvers, MA, USA).

### 3.5. 2D In Vitro Proliferation Assays

Proliferation assays were conducted in 96-well plates using either the Resazurin based fluorescence method or the MTS (3-(4,5-dimethylthiazol-2-yl)-5-(3-carboxymethoxyphenyl)-2-(4-sulfophenyl)-2H-tetrazolium, inner salt)-based absorbance method (CellTiter 96 Aqueous One, Promega Corporation). Fluorescence intensity (590 nm) was measured using an EnVision 2105 multimode instrument (Perkin Elmer, Waltham, MA, USA). Absorbance (490 nm) was measured using a Spectrostar Nano instrument (BMG Labtech, Mornington, Vic., Australia).

### 3.6. In Vitro Analysis of Cell Motility

Transwell migration assays were conducted as described previously [46,86]. Briefly, 2 × 10^5^ cells were seeded into the upper chamber of Fluoroblok (8 μM membrane pore size) inserts (Corning Life Sciences, Oneonta, NY, USA) in serum-free medium. Cells were allowed to migrate (16 h) toward 10% serum-containing medium (700 μL) in the base of the unit. Inserts were incubated with 4 μg/mL calcein^AM^ (Enzo Biochem Inc., New York, NY, USA) prior to imaging and counting of cells using NIH Image J software [88].

### 3.7. Quantitative Real-Time RT-PCR (qRT-PCR)

Total RNA was isolated from cancer cell lines cultured in 2D using either the Direct-zol RNA mini kit (Zymo Corporation, Irvine, CA, USA) or the RNeasy mini kit (Qiagen, Chadstone, Vic., Australia), both featuring on-column DNase I digestion. Resected whole xenograft tumors were homogenized in TRIzol reagent (Thermo Fisher Scientific) using a PowerLyzer 24 (Qiagen) and passed through a 21G needle several times prior to total RNA isolation. First strand cDNA was oligo dT-primed and synthesized from 5 μg total RNA with M-MLV reverse transcriptase in a 20 μL reaction volume (Promega Corporation). One-step (using 4× Fast Virus Master Mix and 50 ng total RNA template per reaction) or two-step (using 2× Fast Universal PCR Master Mix, no AmpErase™ UNG and 250 ng cDNA template per reaction) qRT-PCR was completed using TaqMan assays (Thermo Fisher Scientific). Human RPL37A (Hs01102345_m1) and mouse Rps27 (Mm01218196_g1) were used as internal controls. The following TaqMan assays were used: human Annexin A1 (Hs00167549_m1), mouse Annexin A1 (Mm00440225_m1), mouse cytokeratin 5 (Mm01305291_g1), mouse cytokeratin 14 (Mm00516876_m1), mouse Egfr (Mm01187858_m1), mouse Trp63 (Mm00495793_m1), mouse Sparc/osteonectin (Mm00486332_m1). Two-step qRT-PCR was conducted using 2x Fast SYBR™ Green Master Mix (250 ng cDNA template per reaction) and a pair of oligonucleotide primers (1 μM each, Integrated DNA Technologies, Singapore) as indicated (Table S1). Reactions (10 μL volume) were conducted for 50 cycles using either Step One Plus or ViiA-7 real time PCR instruments (Thermo Fisher Scientific).

### 3.8. Flow Cytometry

Cultured cells were dissociated (TrypLE Express, Gibco, Thermo Fisher Scientific) and filtered. Single cell suspensions were then blocked (10% horse serum in PBS/0.5 mM EDTA, Gibco, Thermo Fisher Scientific) for 30 min prior to incubation with the directly conjugated primary antibodies (listed above) in FACS buffer (3% horse serum in PBS/0.5 mM EDTA) for 1 h at 4 °C. Following washing with FACS buffer, cell pellets were gently resuspended in FACS buffer (200 μL) containing SYTOX Blue viability dye (Thermo Fisher Scientific). Cell analysis was conducted using a FACS Canto II instrument (BD Biosciences) with compensation as required. Cell sorting was conducted using a FACS ARIAIII instrument (BD Biosciences) with compensation as required. Data were analyzed using FlowJo software (v10, BD Biosciences).

### 3.9. Immunofluorescence and Phase Contrast Microscopy

Phase contrast and greyscale fluorescent (eGFP) images of cultured cells were captured using an inverted fluorescent microscope (Leica DMIRB, Leica Microsystems, Macquarie Park, NSW, Australia) and SPOT digital camera and software (Diagnostic Instruments, Inc. Sterling Heights, MI, USA). Sorted parental PyMT cells were seeded onto collagen I coated glass chamber slides (Nunc, Lab-Tek, Sigma-Aldrich), cultured for 6 days, and immunofluorescence carried out using the antibodies (above) as described previously [47]. Nuclei were visualized using 4′,6-diamidino-2-phenylindole (DAPI, Sigma Aldrich). Phase contrast and fluorescent images of sorted parental PyMT cells were captured using an Olympus inverted microscope and digital camera and cellSens imaging software (Olympus, Notting Hill, Vic., Australia) at the Australian Cancer Research Foundation Centre for Imaging the Tumour Environment (ONJCRI).

### 3.10. Confocal Microscopy

Cells were grown on plastic in 8-well chamber slides (Permanox, Nunc, Lab-Tek, Sigma-Aldrich) and immunofluorescence carried out as described previously [47]. Nuclei were visualized using 4′,6-diamidino-2-phenylindole (DAPI, Sigma Aldrich). Images were generated using an LSM780-inverted confocal microscope (Carl Zeiss, North Ryde, NSW, Australia) with a 10x objective and associated ZEN software (Carl Zeiss).

### 3.11. Monitoring of Tumor Growth In Vivo

Female C57BL/6 immuno-competent mice and C57BL/6NTac;B10(Cg)-Rag^2tm1Fwa^Il2rg^tm1Wjl^ immuno-deficient mice (obtained from Walter and Eliza Hall Institute of Medical Research, Parkville, Vic., Australia) and female NOD.Cg-Prkdc^scid^Il2rg^tm1Wjl^/SzJ (NSG) immuno-deficient mice (obtained from Garvan Institute of Medical Research, Darlinghurst, NSW, Australia) were maintained in a specific pathogen-free environment and fed ad libitum. Procedures involving mice were conducted at the Peter MacCallum Cancer Centre or at the Olivia Newton-John Cancer Research Institute and conformed to National Health and Medical Research Council animal ethics guidelines and were approved by the Animal Experimentation and Ethics Committee’s (AEEC) of the Peter MacCallum Cancer Centre and Austin Health, respectively. Mammary tumors were established orthotopically in the right-side inguinal mammary gland of 6–8-week-old mice. Briefly, viable tumor cells resuspended in PBS (PyMTneoLUC-derived lines 2.5 ×10^5^ cells; EO771.LMB-derived lines 2 × 10^5^ cells; SUM149scidLuc2Ch-derived lines 1 × 10^6^ cells) were mixed with 10–30% Cultrex (Trevigen) and inoculated in a total volume of 20 μL per mouse. Tumor size was measured using electronic calipers and volumes estimated using the modified ellipsoidal formula: volume = 0.5 (length × width^2^) [89]. Tumor growth was also monitored by in vivo bioluminescence imaging as described previously [47,86,87], using a Xenogen Lumina II in vivo imaging system (Caliper Life Sciences, Perkin Elmer, Hopkinton, MA, USA). Whole primary tumors were imaged ex vivo using a fluorescent dissecting stereomicroscope (SZX12, Olympus, Notting Hill, Vic, Australia) and a SPOT digital camera and software (Diagnostic Instruments, Inc. Sterling Heights, MI, USA).

### 3.12. Histology and Immunohistochemistry

Histology and immunohistochemistry were carried out as described previously [47,86]. Briefly, slides were subjected to heat-mediated antigen prior to incubation with primary and then HRP-conjugated secondary antibodies. Signals were visualized using a 3,3′-diaminobenzidine peroxidase substrate kit (Dako). Images were generated using a digital pathology slide scanner (Aperio, Leica Biosystems) at the Australian Cancer Research Foundation Centre for Imaging the Tumour Environment (ONJCRI) and associated ImageScope software (Aperio, Leica Biosystems).

### 3.13. Bioinformatics

Associations between Annexin A1 expression and patient survival in breast cancer subtypes were analyzed using the Kaplan–Meier Plotter (www.kmplot.com, accessed on 5 March 2021) [43]. Analysis of Annexin A1 expression in TNBC subtypes was conducted using data from Lehmann et al., 2011 [5].

### 3.14. Statistical Analyses

Raw expression data were downloaded from the TCGA breast cancer dataset [38], and analyzed using the software package, RSEM (RNA-Seq by Expectation Maximization) [90]. Comparison of means was conducted using the Student’s *t*-test or one-way analysis of variance (ANOVA) as appropriate using GraphPad Prism (v8) software (GraphPad Software, San Diego, CA, USA). In the case of ANOVA, Dunnett’s post-hoc test for multiple comparisons was used when comparing to a single control group, whereas Tukey’s post-hoc test was used when comparing all groups to each other. Cell line and tumor growth curves were analyzing using mixed effects linear regression modelling [46,47,86]. If the curves failed to converge then the area under the curve (AUC) method was applied and two sample *t*-tests used to compare the groups. *p* < 0.05 was taken as significant. *, *p* < 0.05. **, *p* < 0.01. *** *p* < 0.001. ****, *p* < 0.0001. S.D., standard deviation. SEM, standard error of the mean.

## 4. Conclusions

Our comprehensive analysis of TNBC cell lines with Annexin A1 knockdown demonstrates a novel cell-autonomous role for Annexin A1 in the promotion of tumor forming capacity in a model of human breast cancer. A corollary of this is that certain basal-like TNBC tumors may have a dependence on high levels of Annexin A1 expression in the neoplastic cells for their ongoing viability. Further research is required to uncover the relevant features of human breast cancers that might render them vulnerable to inhibition of Annexin A1 function(s).

## Figures and Tables

**Table 1:** Annexin A1 expression levels in primary breast tumors and outcome in different molecular subtypes of TNBC hazard ratios (H.R.s), 95% confidence intervals, logrank *p* values and sample sizes are shown. The clinical follow up was 200 months. * An upper tertile cut off was used for Annexin A1 mRNA expression in all analyses except BL-2 where a lower quartile cut off was used. ER, estrogen receptor. HER2, human epidermal growth factor receptor 2.

TNBC Subtype	Abbreviation	Sample Size (*n*)	Hazard Ratio & (95% CI)	Logrank *p*
Basal-like-1	BL-1	171	1.57 (0.96–2.55)	0.0687
Basal-like-2 *	BL-2	76	3.12 (1.09–8.92)	0.0257
Mesenchymal	M	177	1.25 (0.80–1.96)	0.3215
Luminal androgen receptor positive	LAR	203	0.87 (0.54–1.42)	0.5791
Basal intrinsic subtype	Basal	618	1.77 (1.37–2.28)	<0.0001
ER−	ER−	801	0.88 (0.52–1.50)	0.6380
HER2+	HER+	251	0.85 (0.56–1.28)	0.4342
Luminal A	LumA	1933	0.97 (0.81–1.17)	0.7736
Luminal B	LumB	1149	0.95 (0.77–1.17)	0.6305

## Data Availability

The data presented in this study are available on request from the corresponding authors.

## Supplementary Materials

The following are available online at https://www.mdpi.com/2072-6694/13/5/1154/s1, Figure S1: Expression of Annexin A1 mRNA from Affymetrix microarray analysis of human breast cancer cell lines. Figure S2: Annexin A1 expression is not dysregulated in models of metastatic breast cancer. Figure S3: Annexin A1 depletion does not alter primary tumor growth or spontaneous metastasis of MDA-MB-231_HM orthotopic xenografts. Figure S4: Immunofluorescence analysis of Annexin A1 expression in SUM149 cells. Figure S5: Immunohistochemical analysis of protein expression in SUM149_shNC and SUM149_shANXA1 primary mammary xenografts. Figure S6: Expression of Annexin A1 in mouse mammary tumor lines. Figure S7: Expression of estrogen receptor alpha (ERα), progesterone receptor (PR), and erb-b2 in spontaneous and transgenic allograft mouse mammary tumor models. Figure S8: Knockdown of Annexin A1 in PyMTneoLUC and EO771.LMB mouse mammary tumor lines. Figure S9: Growth of control and Annexin A1 depleted PyMTneoLUC mammary tumors in wild-type C57BL/6 mice. Figure S10: Optical imaging of control and Annexin-A1-depleted PyMTneoLUC mammary tumors in wild-type C57BL/6 mice. Figure S11: Optical imaging of control and Annexin-A1-depleted PyMTneoLUC mammary tumors in immuno-deficient C57BL/6 mice. Figure S12: Evaluation of EO771.LMB_shNC and EO771.LMB_shAnxa1_4 cell lines in vivo. Figure S13: In vitro proliferation rates of control and Annexin-A1-depleted PyMTneoLUC cells. Figure S14: Immunofluorescence analysis of E-cadherin protein and filamentous actin (F-actin) in confluent cultures of PyMTneoLUC stable transfectants. Figure S15: The microtubule network as assessed by immunofluorescence staining of β-tubulin in post-confluent cultures of PyMTneoLUC stable transfectants. Figure S16: Western blot analysis of E-cadherin (A) and vimentin (B) expression in PyMTneoLUC (i), EO771.LMB (ii), and SUM149 (iii) stable transfectants. Figure S17: qRT-PCR analysis of gene expression in SUM149_shNC and SUM149_shANXA1 cell lines. Figure S18: Immunohistochemical analysis of epithelial marker expression in whole PyMTneoLUC_shAnxa1_1 mammary tumors formed in immuno-deficient C57BL/6 mice. Figure S19: Immunohistochemical analyses of normal mouse tissue. Figure S20: Cell surface expression of markers of tumor initiating cells in PyMTneoLUC transfectants; Figure S21: Original Western blot images for Figure 2B. Figure S22: Original Western blot images for Figure S16. Table S1: List of primer sequences for SYBR green qRT-PCR.
