# Supplementary material for: Annexin A1 Is Required for Efficient Tumor Initiation and Cancer Stem Cell Maintenance in a Model of Human Breast Cancer
Source: Cancers (Basel). 2021 Mar 8;13(5):1154. doi: 10.3390/cancers13051154 (PMC7962654; doi:10.3390/cancers13051154)
Supplement: Supplementary file 1 [file cancers-13-01154-s001.pdf]

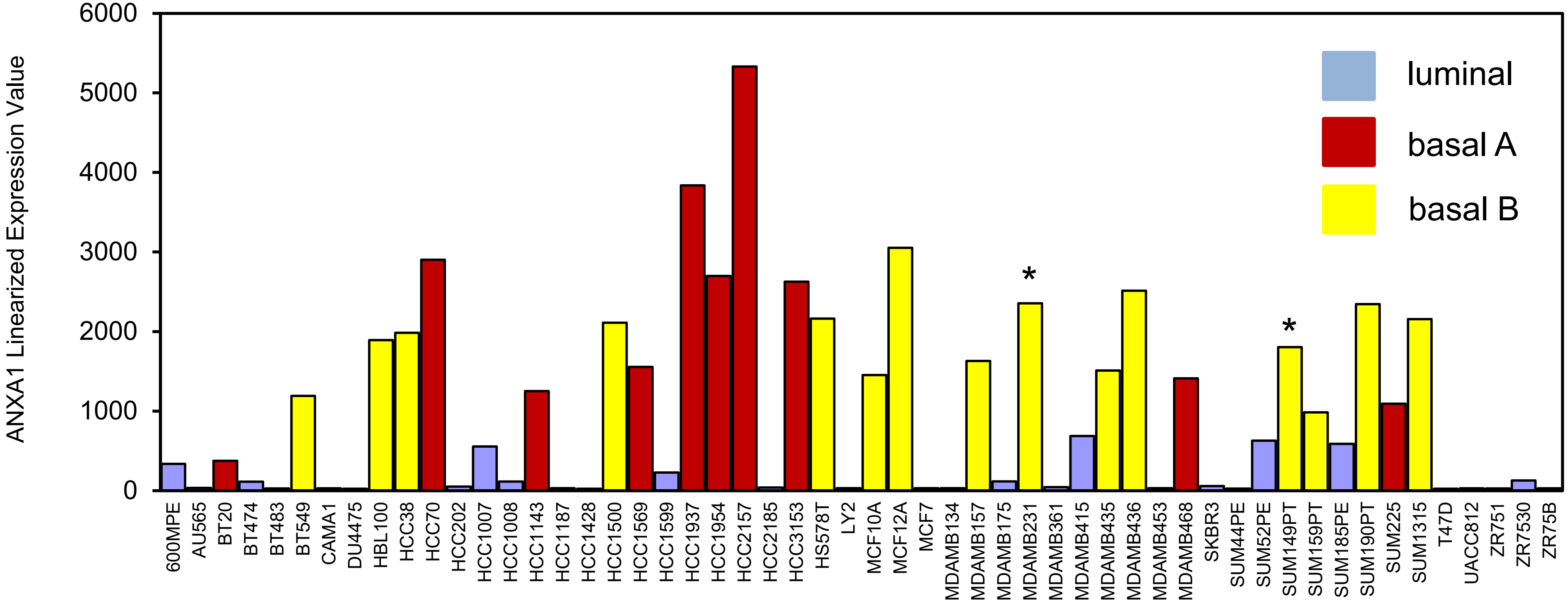
ANXA1 Linearized Expression Value
6000
5000
4000
3000
2000
1000
0
luminal
basal A
basal B
*
*
600MPE
AU565
BT20
BT474
BT483
BT549
CAMA1
DU4475
HBL100
HCC38
HCC70
HCC202
HCC1007
HCC1008
HCC1143
HCC1187
HCC1428
HCC1500
HCC1569
HCC1599
HCC1937
HCC1954
HCC2157
HCC2185
HCC3153
HS578T
LY2
MCF10A
MCF12A
MCF7
MDAMB134
MDAMB157
MDAMB175
MDAMB231
MDAMB361
MDAMB415
MDAMB435
MDAMB436
MDAMB453
MDAMB468
SKBR3
SUM44PE
SUM52PE
SUM149PT
SUM159PT
SUM185PE
SUM190PT
SUM225
SUM1315
T47D
UACC812
ZR751
ZR7530
ZR75B

Figure S1

Figure S2

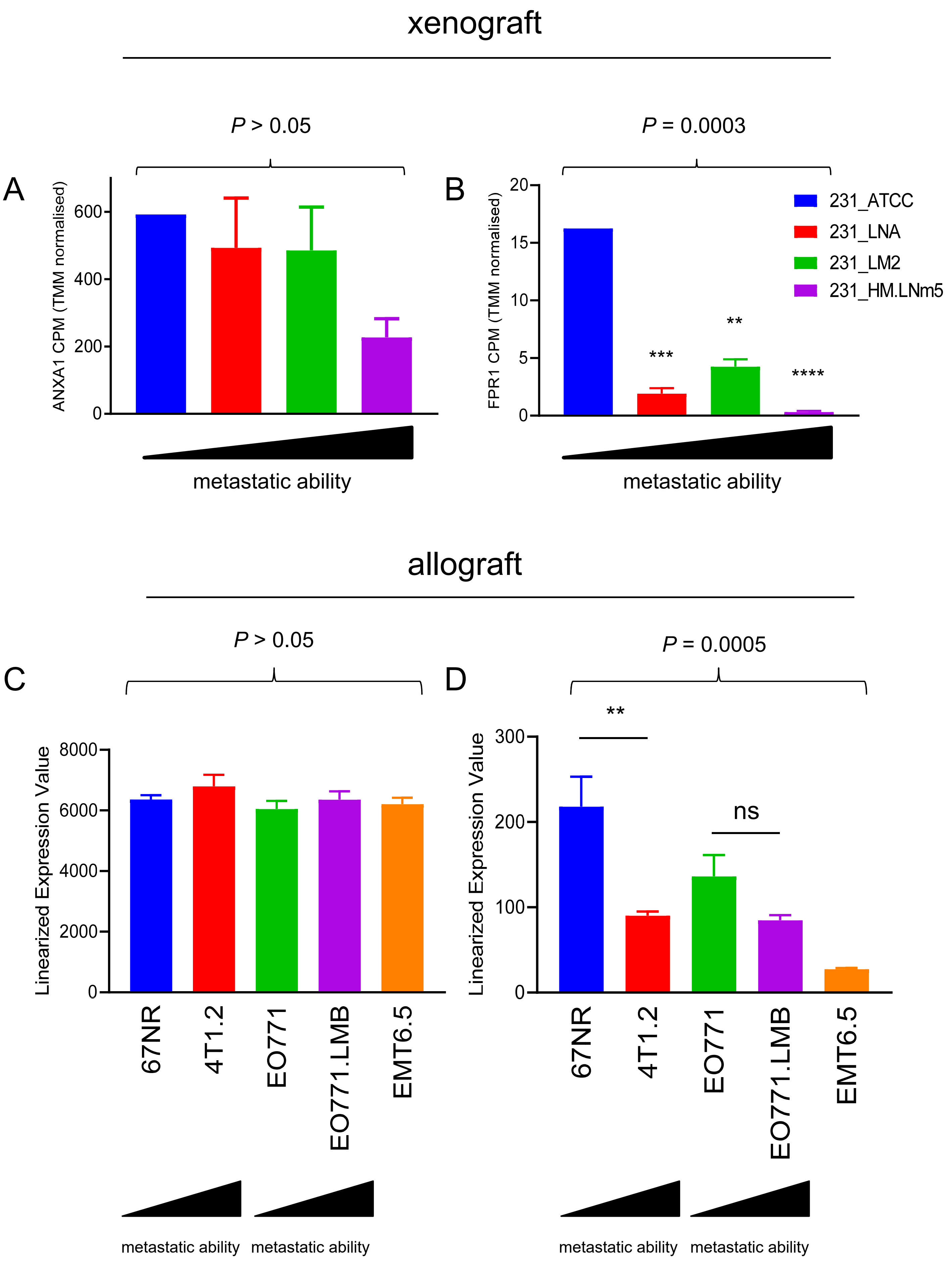
xenograft
P > 0.05
P = 0.0003
A
B
ANXA1 CPM (TMM normalised)
FPR1 CPM (TMM normalised)
231_ATCC
231_LNA
231_LM2
231_HM.LNm5
***
**
****
metastatic ability
metastatic ability
allograft
P > 0.05
P = 0.0005
C
D
Linearized Expression Value
Linearized Expression Value
**
ns
67NR
4T1.2
EO771
EO771.LMB
EMT6.5
metastatic ability
metastatic ability
metastatic ability
metastatic ability

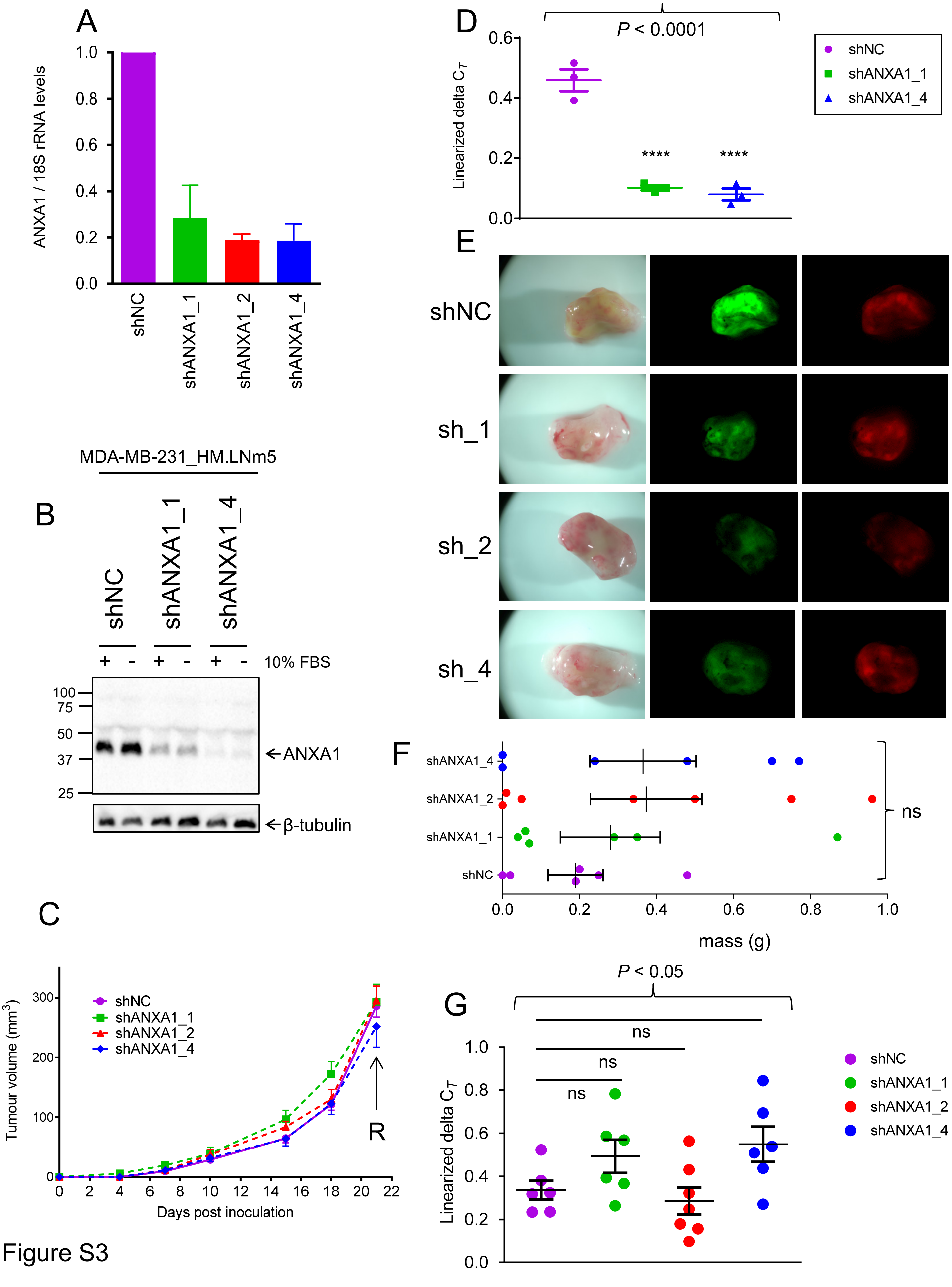
A
ANXA1 / 18S rRNA levels
shNC
shANXA1_1
shANXA1_2
shANXA1_4
MDA-MB-231_HM.LNm5
B
shNC
shANXA1_1
shANXA1_4
10% FBS
ANXA1
β-tubulin
C
Tumour volume (mm3)
shNC
shANXA1_1
shANXA1_2
shANXA1_4
R
Days post inoculation
D
P < 0.0001
Linearized delta CT
****
E
shNC
sh_1
sh_2
sh_4
F
ns
mass (g)
G
P < 0.05
ns
Linearized delta CT

Figure S3

Figure S4

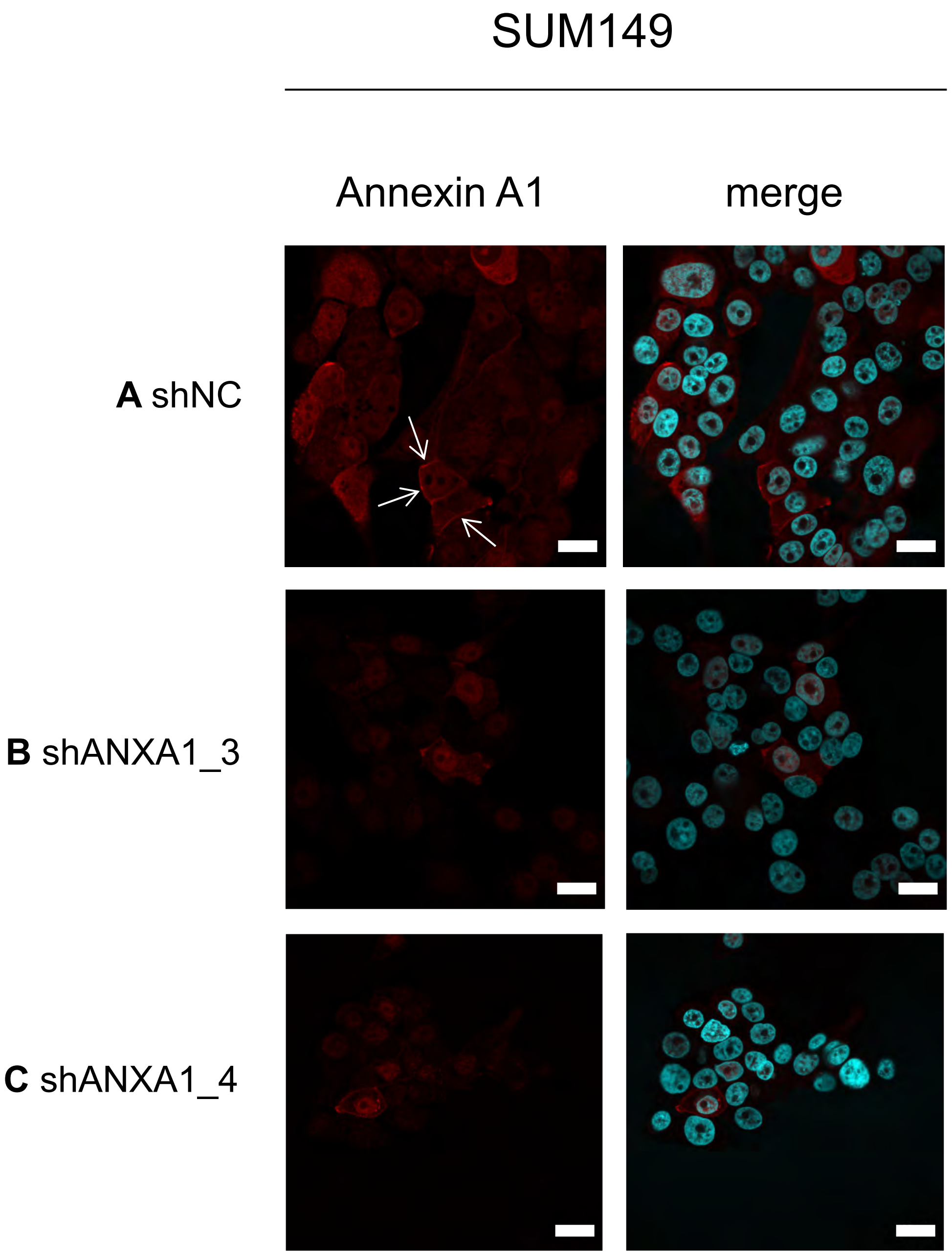

Figure S5

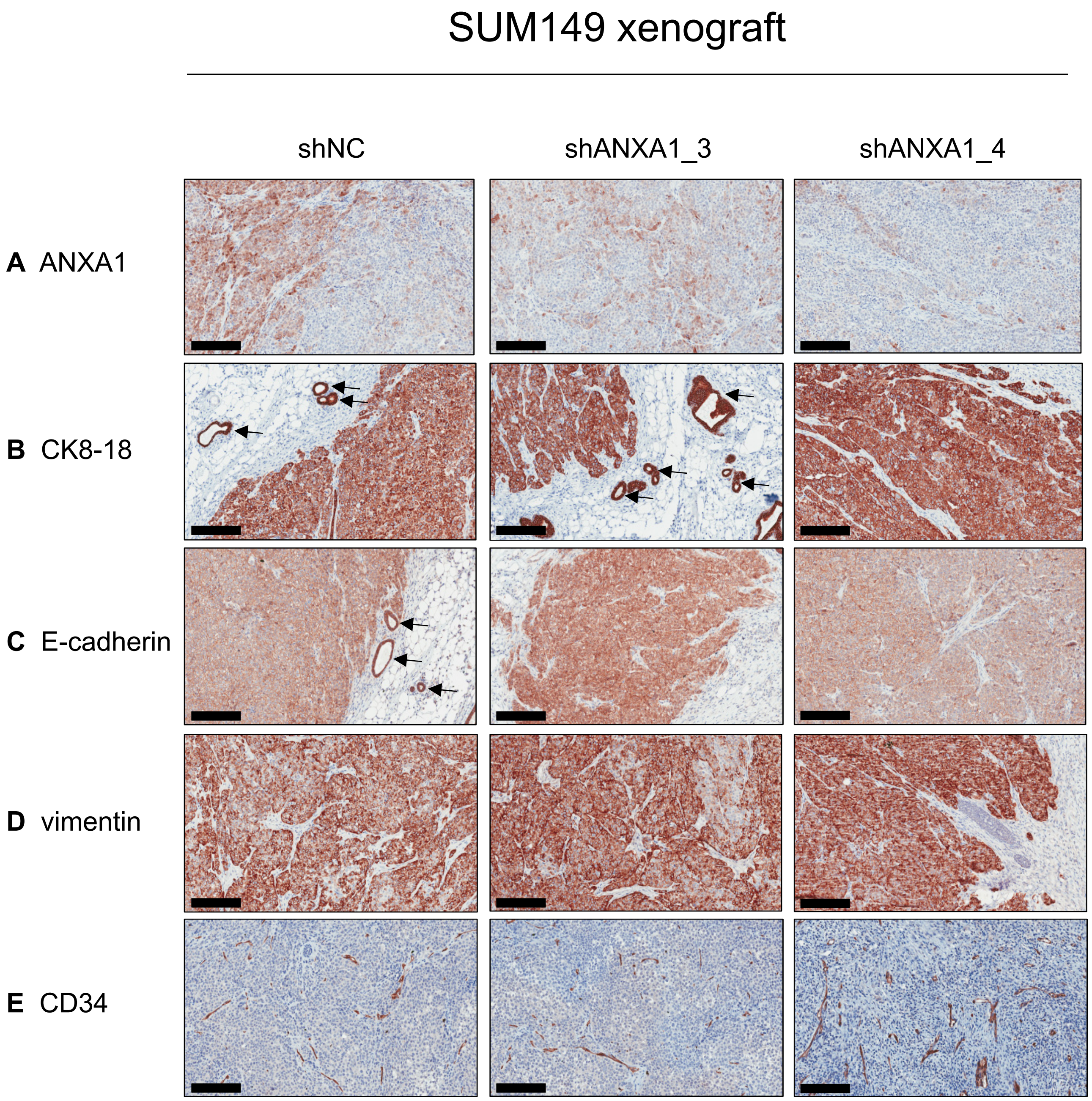
SUM149 xenograft
shNC
shANXA1_3
shANXA1_4
A ANXA1
B CK8-18
C E-cadherin
D vimentin
E CD34

A

Line	Strain	GEMM or spontaneous	Metastatic potential
PyMT (neoLUC)	C57BL/6	GEMM (PyMT)	No
AT-3 (neoLUC)	C57BL/6	GEMM (PyMT)	No
EO771.LMB	C57BL/6	Spontaneous	Yes
HB158+	Mixed	GEMM (*Pik3ca*)	No*
MH248	Mixed	GEMM (*Pik3ca*)	No*
EMT6.5	BALB/c	spontaneous	Yes
4T1.2	BALB/c	spontaneous	Yes
4T1.13	BALB/c	spontaneous	Yes
H2N100	BALB/c	GEMM (*Neu*)	Unknown

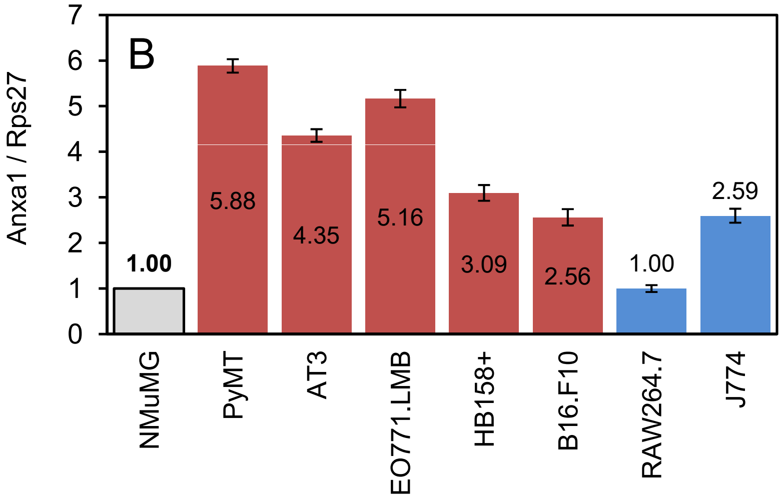

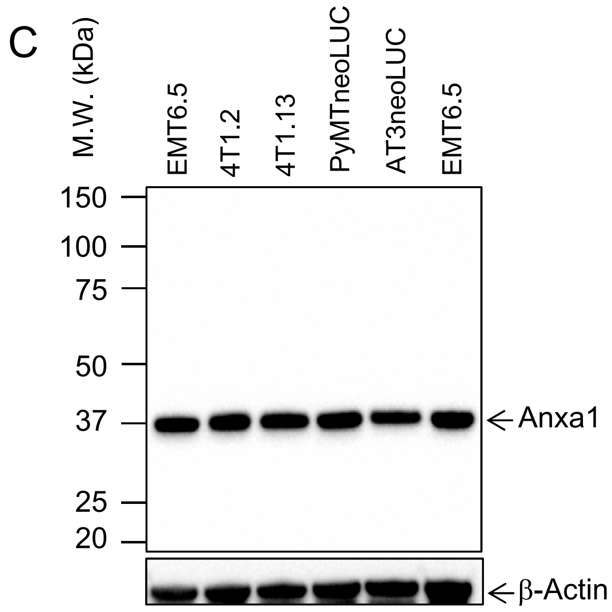

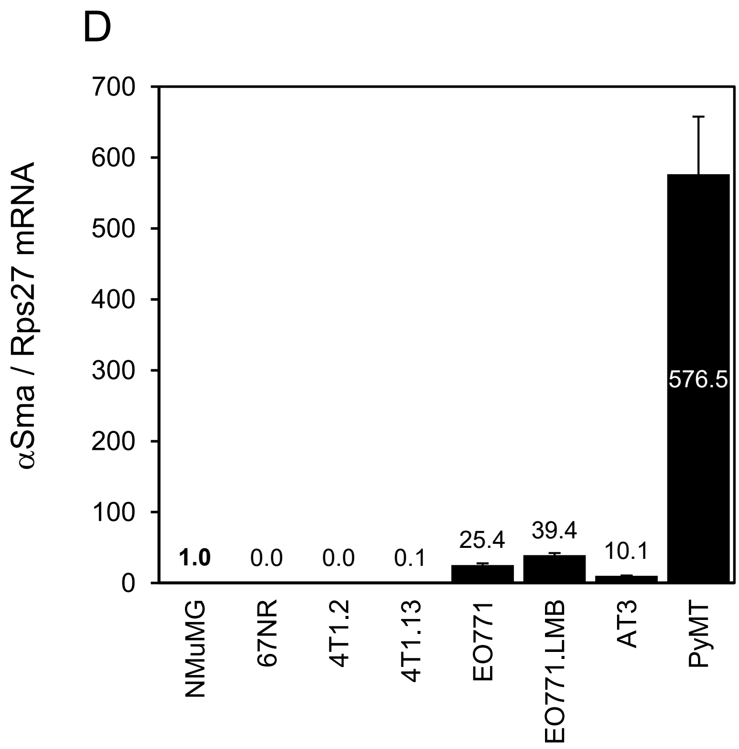

Figure S6

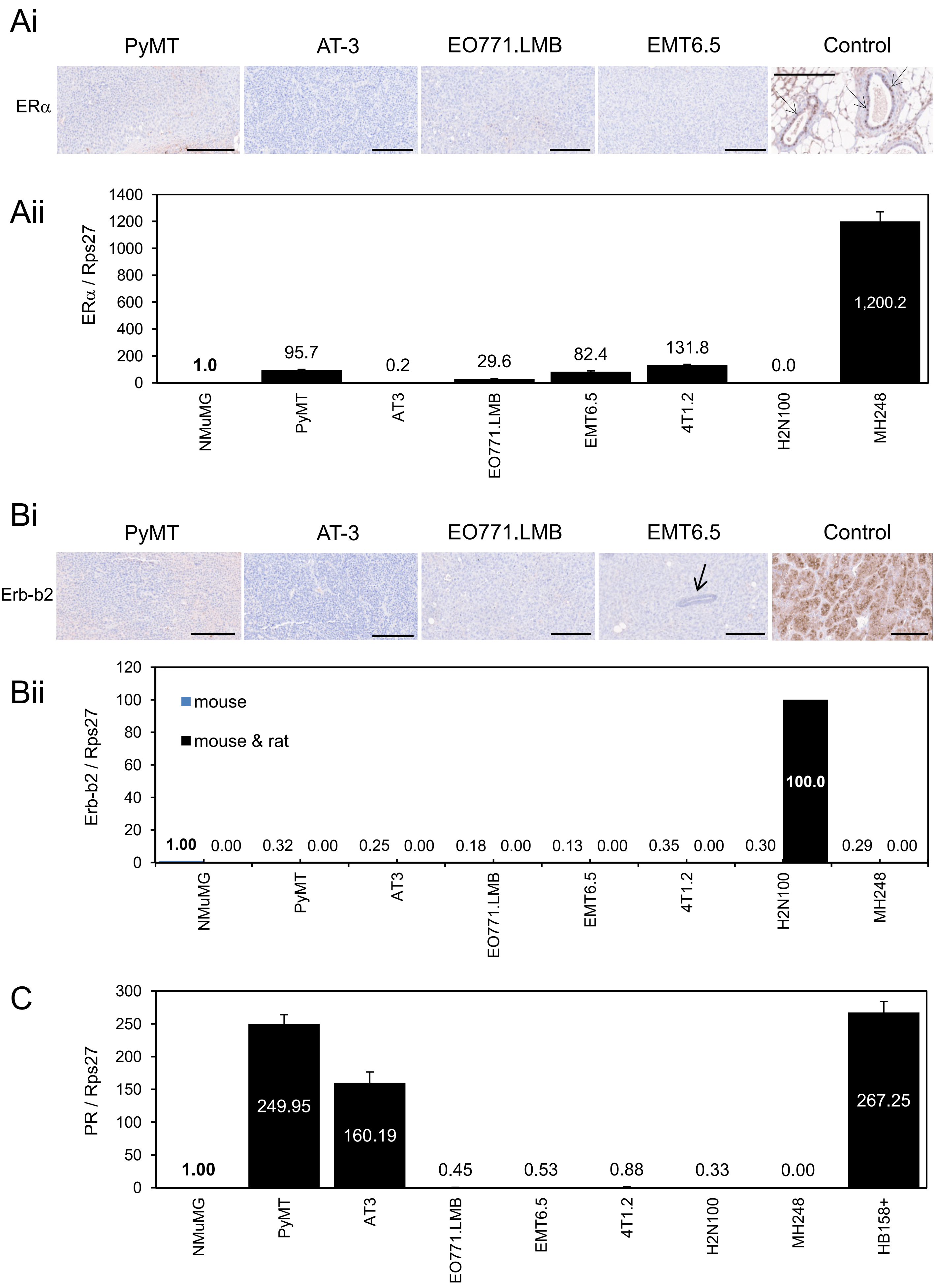
Ai
PyMT
AT-3
EO771.LMB
EMT6.5
Control
ERα
Aii
ERα / Rps27
1400
1200
1000
800
600
400
200
0
1.0
95.7
0.2
29.6
82.4
131.8
0.0
1,200.2
NMuMG
PyMT
AT3
EO771.LMB
EMT6.5
4T1.2
H2N100
MH248
Bi
PyMT
AT-3
EO771.LMB
EMT6.5
Control
Erb-b2
Bii
Erb-b2 / Rps27
120
100
80
60
40
20
0
mouse
mouse & rat
1.00
0.00
0.32
0.00
0.25
0.00
0.18
0.00
0.13
0.00
0.35
0.00
0.30
100.0
0.29
0.00
NMuMG
PyMT
AT3
EO771.LMB
EMT6.5
4T1.2
H2N100
MH248
C
PR / Rps27
300
250
200
150
100
50
0
1.00
249.95
160.19
0.45
0.53
0.88
0.33
0.00
267.25
NMuMG
PyMT
AT3
EO771.LMB
EMT6.5
4T1.2
H2N100
MH248
HB158+

Figure S7

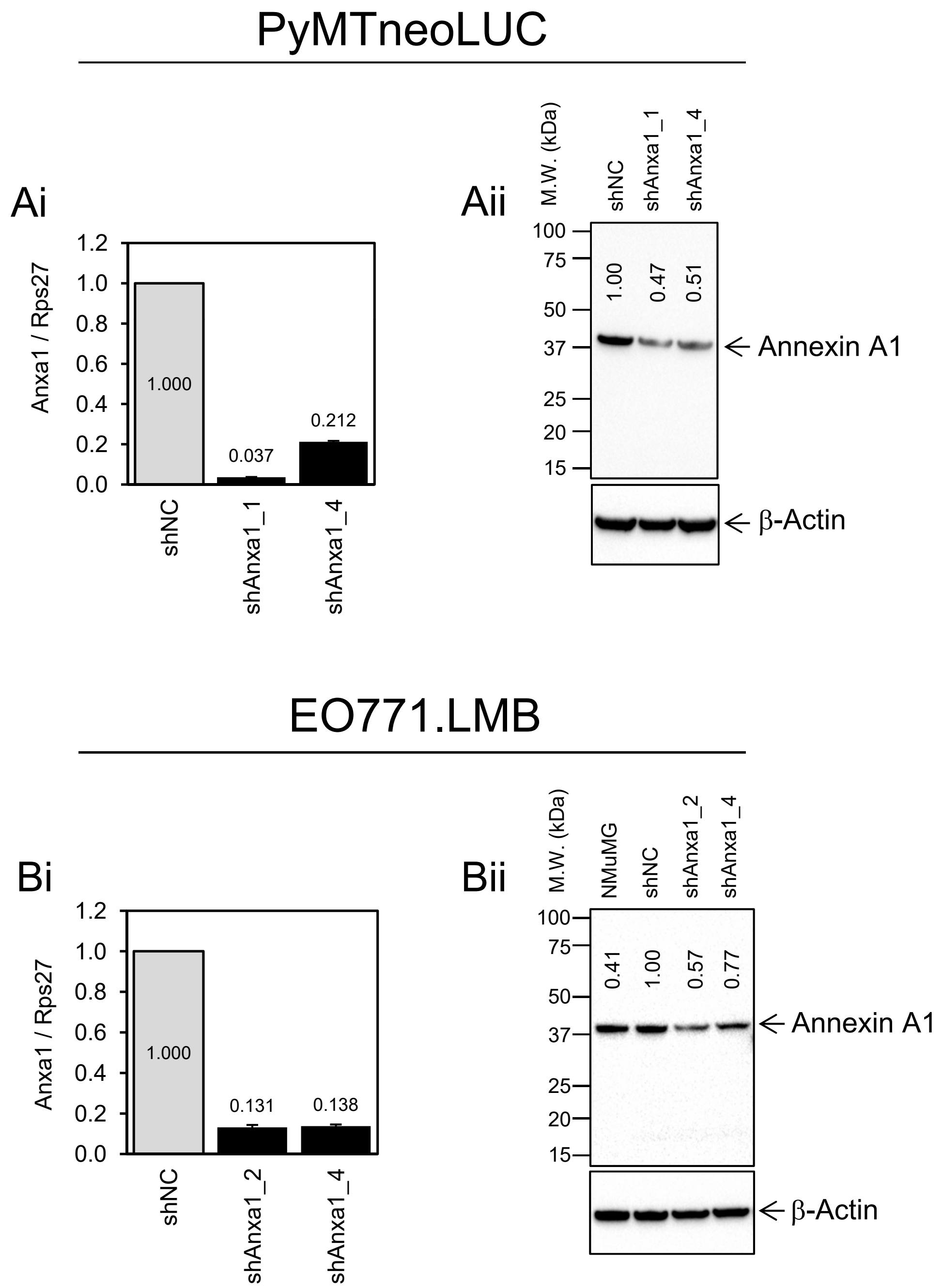
PyMTneoLUC
Ai
Anxa1 / Rps27
1.2
1.0
0.8
0.6
0.4
0.2
0.0
1.000
0.037
0.212
shNC
shAnxa1_1
shAnxa1_4
Aii
M.W. (kDa)
shNC
shAnxa1_1
shAnxa1_4
100
75
50
37
25
20
15
1.00
0.47
0.51
Annexin A1
β-Actin
EO771.LMB
Bi
Anxa1 / Rps27
1.2
1.0
0.8
0.6
0.4
0.2
0.0
1.000
0.131
0.138
shNC
shAnxa1_2
shAnxa1_4
Bii
M.W. (kDa)
NMuMG
shNC
shAnxa1_2
shAnxa1_4
100
75
50
37
25
20
15
0.41
1.00
0.57
0.77
Annexin A1
β-Actin

Figure S8

Figure S9

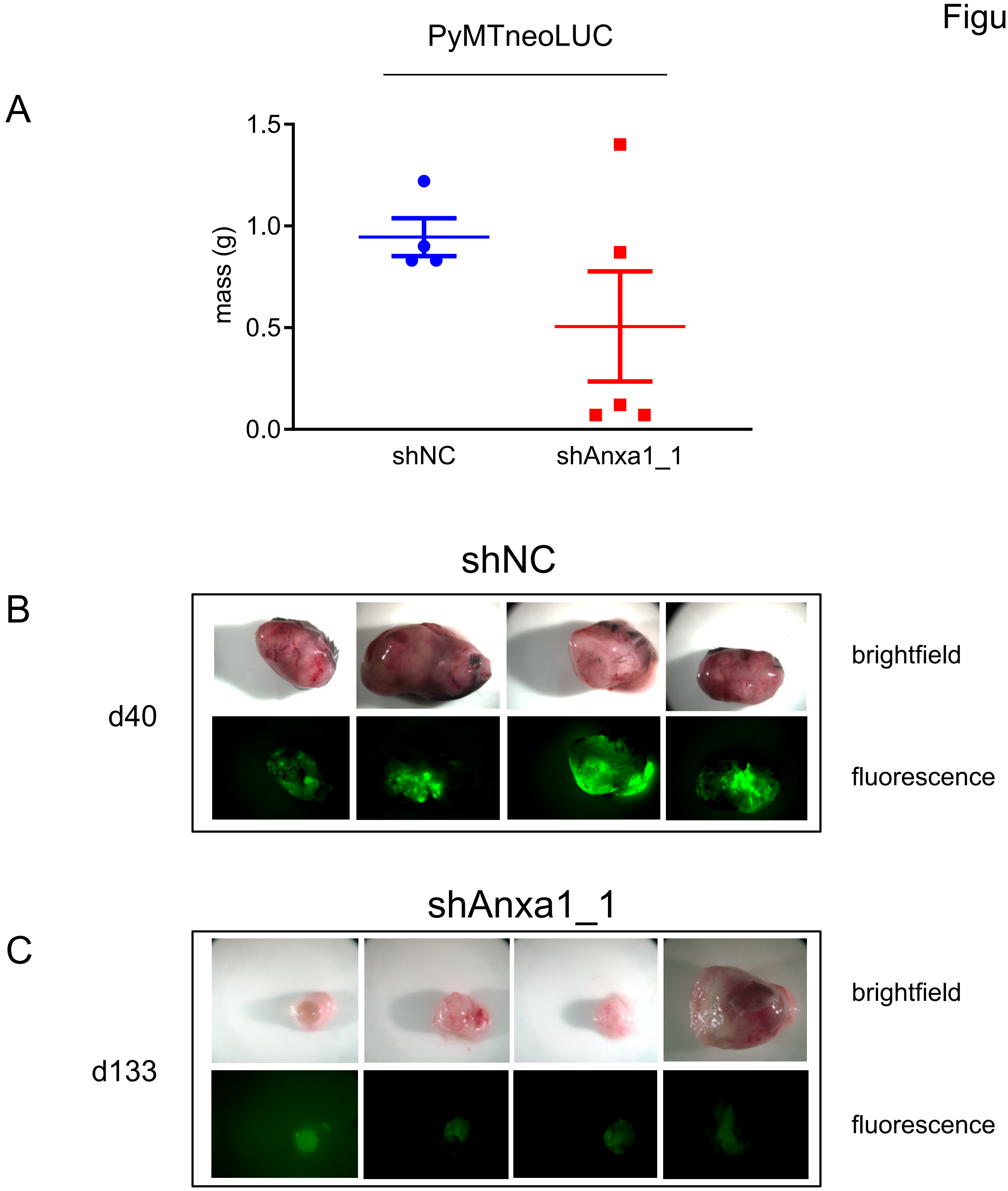
PyMTneoLUC
A
1.5
1.0
0.5
0.0
mass (g)
shNC
shAnxa1_1
shNC
B
d40
brightfield
fluorescence
shAnxa1_1
C
d133
brightfield
fluorescence

Figure S10

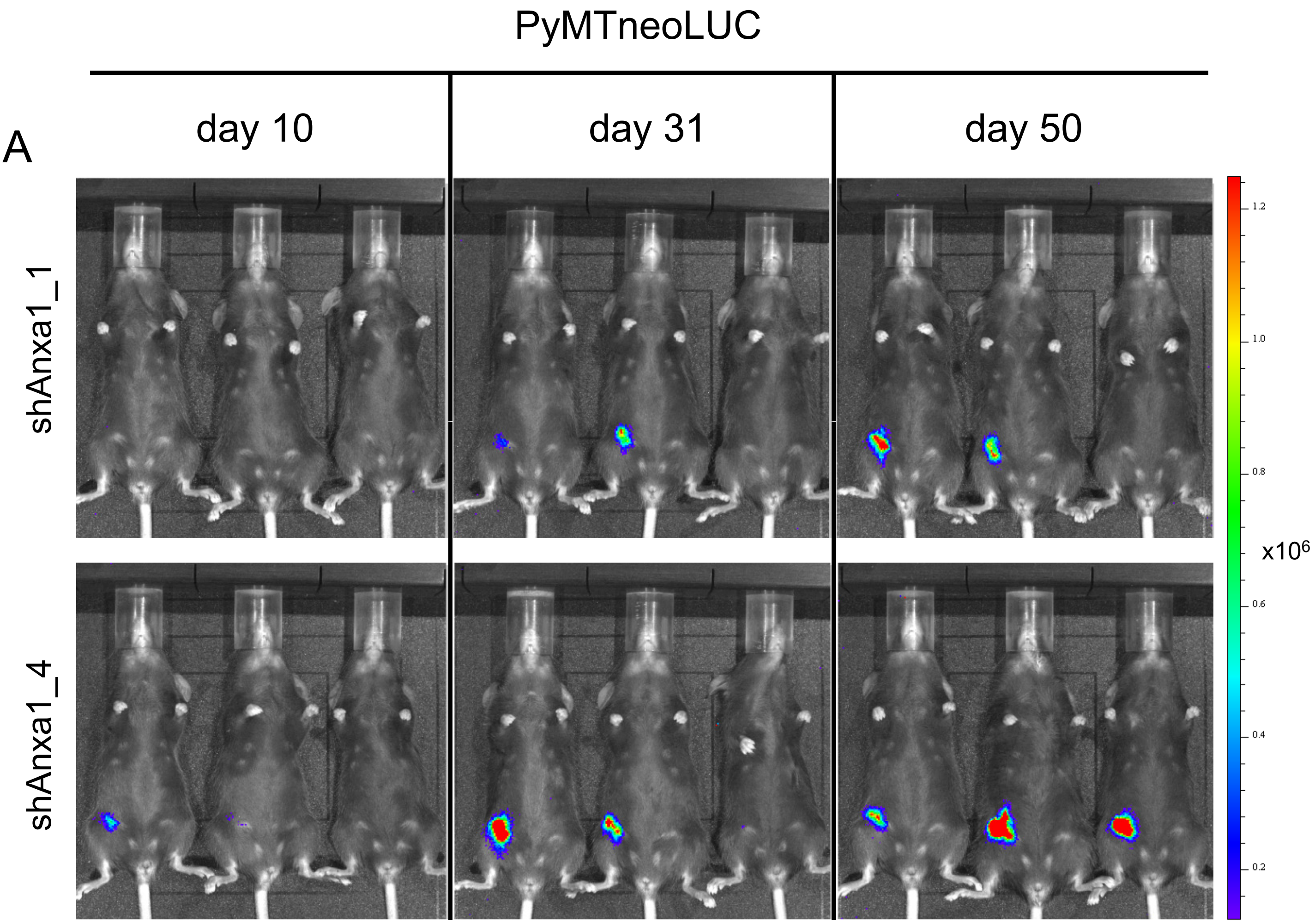

PyMTneoLUC
A
day 10
day 31
day 50
shAnxa1_1
shAnxa1_4
x10^6
1.2
1.0
0.8
0.6
0.4
0.2

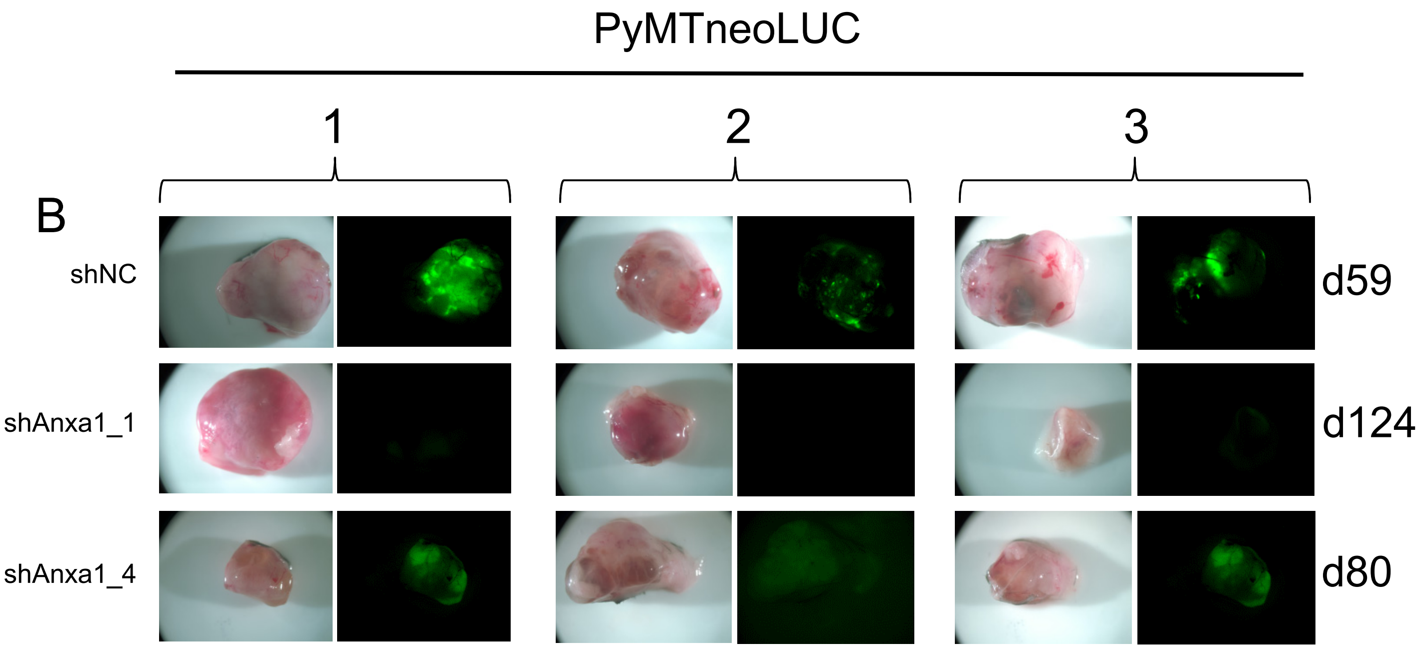

PyMTneoLUC
B
1
2
3
shNC
shAnxa1_1
shAnxa1_4
d59
d124
d80

Figure S11

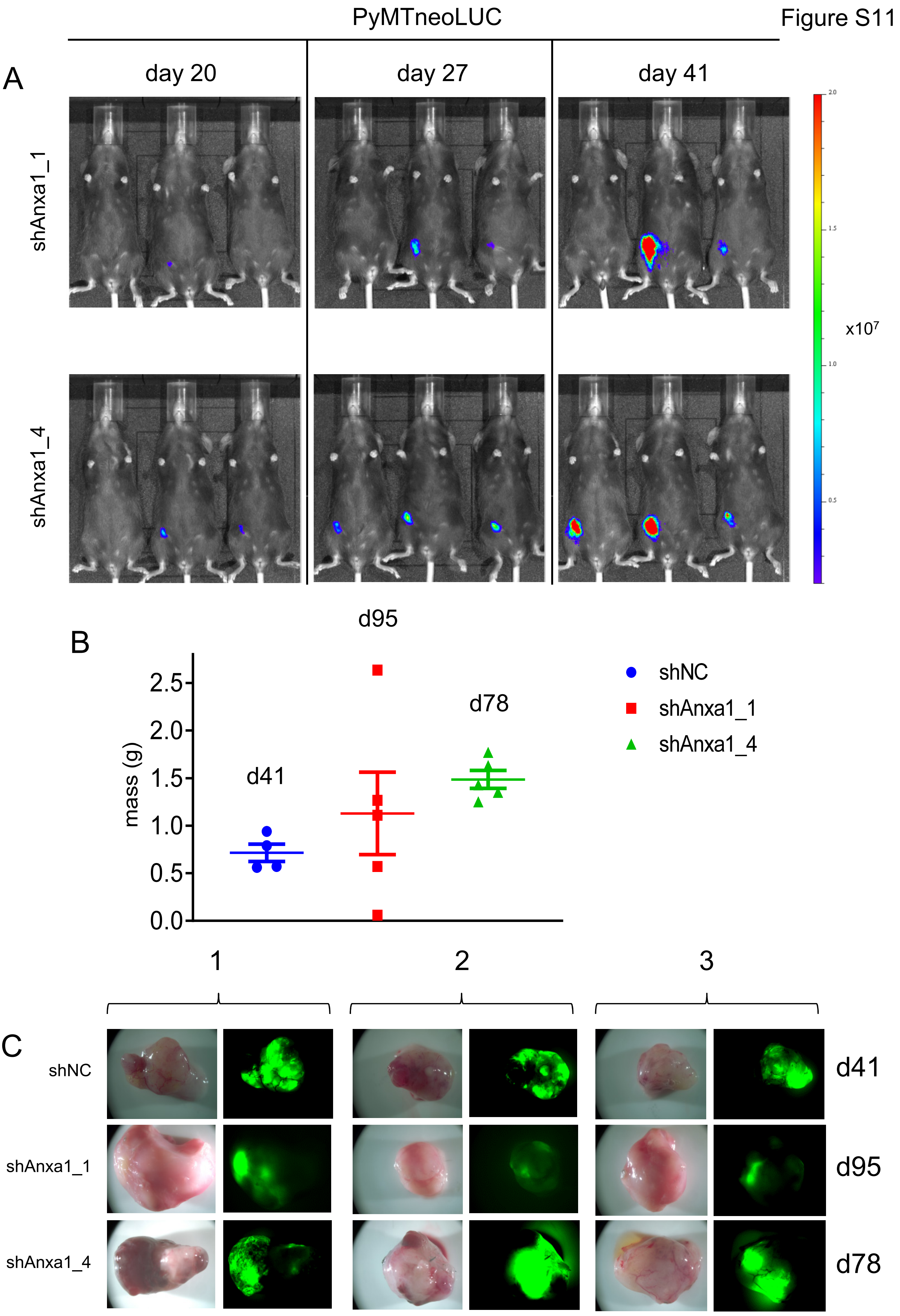
PyMTneoLUC
A
day 20
day 27
day 41
shAnxa1_1
shAnxa1_4
2.0
1.5
1.0
0.5
x10^7
B
d95
d78
d41
mass (g)
2.5
2.0
1.5
1.0
0.5
0.0
shNC
shAnxa1_1
shAnxa1_4
1
2
3
C
shNC
shAnxa1_1
shAnxa1_4
d41
d95
d78

Figure S12

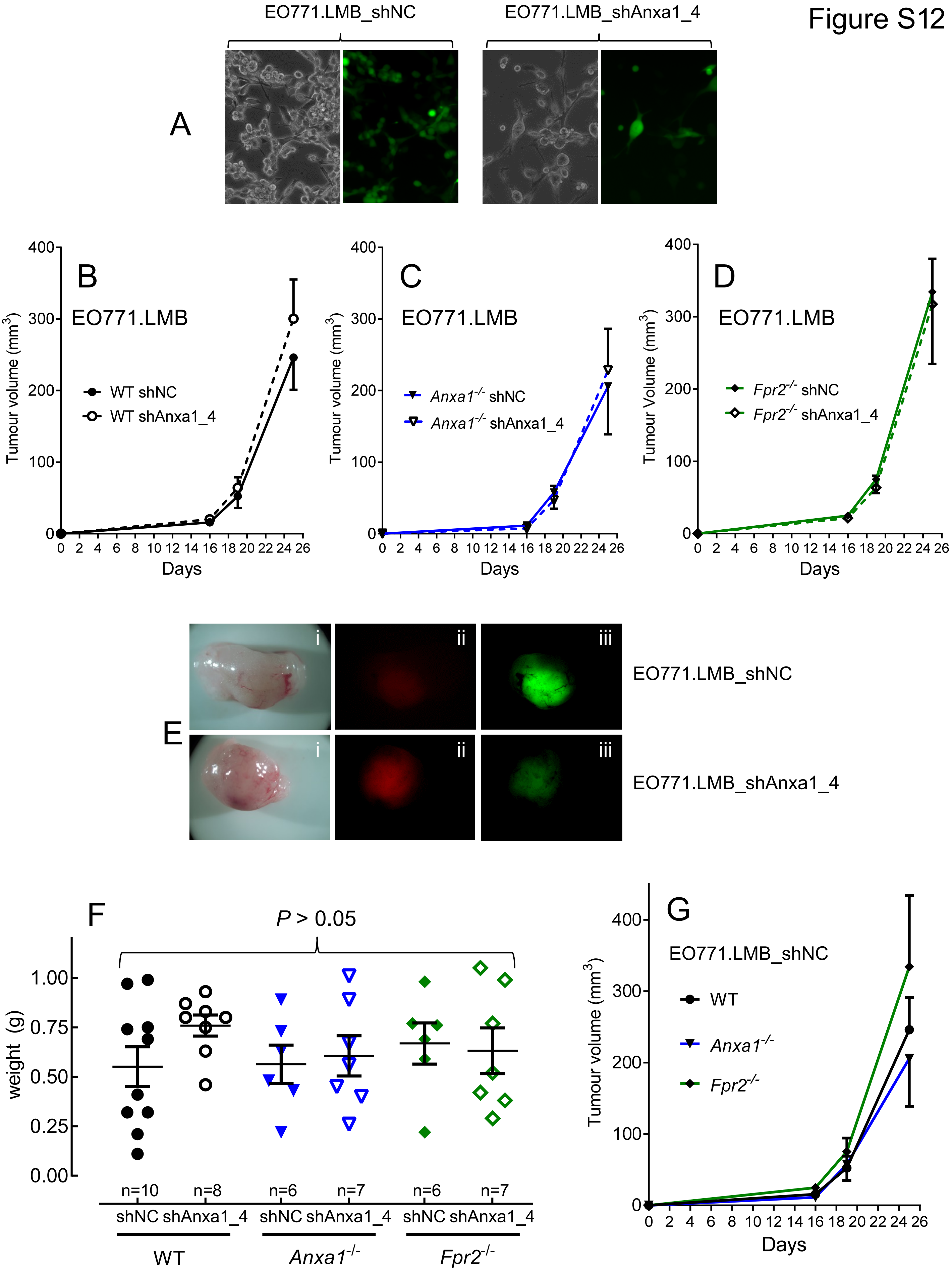

A
EO771.LMB_shNC
EO771.LMB_shAnxa1_4
B
EO771.LMB
WT shNC
WT shAnxa1_4
Tumour volume (mm3)
Days
C
EO771.LMB
Anxa1-/- shNC
Anxa1-/- shAnxa1_4
Tumour volume (mm3)
Days
D
EO771.LMB
Fpr2-/- shNC
Fpr2-/- shAnxa1_4
Tumour Volume (mm3)
Days
E
i
ii
iii
EO771.LMB_shNC
EO771.LMB_shAnxa1_4
F
P > 0.05
weight (g)
n=10
n=8
n=6
n=7
n=6
n=7
shNC shAnxa1_4
shNC shAnxa1_4
shNC shAnxa1_4
WT
Anxa1-/-
Fpr2-/-
G
EO771.LMB_shNC
WT
Anxa1-/-
Fpr2-/-
Tumour volume (mm3)
Days

Figure S13

PyMTneoLUC

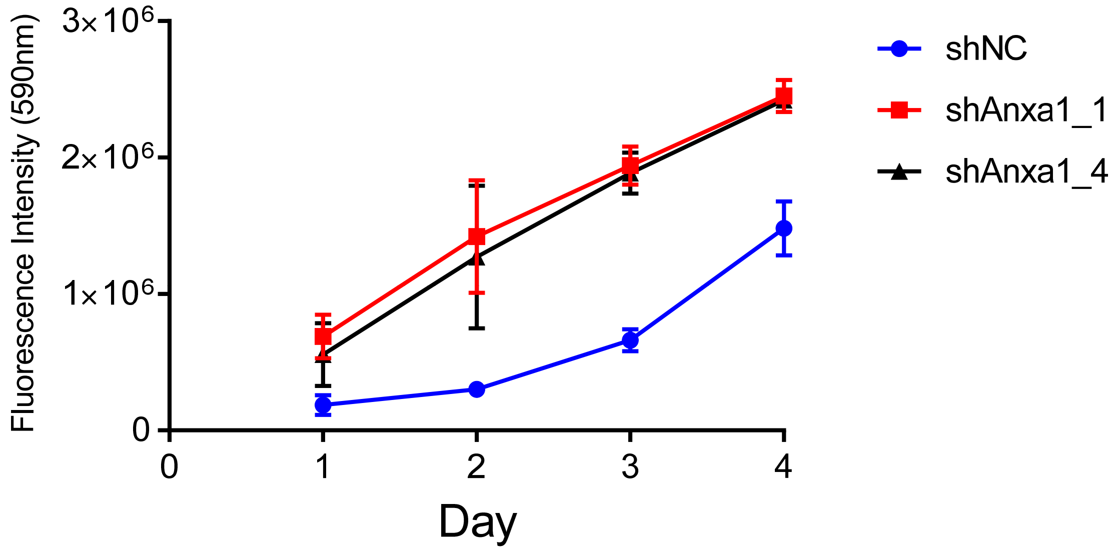

Figure S14

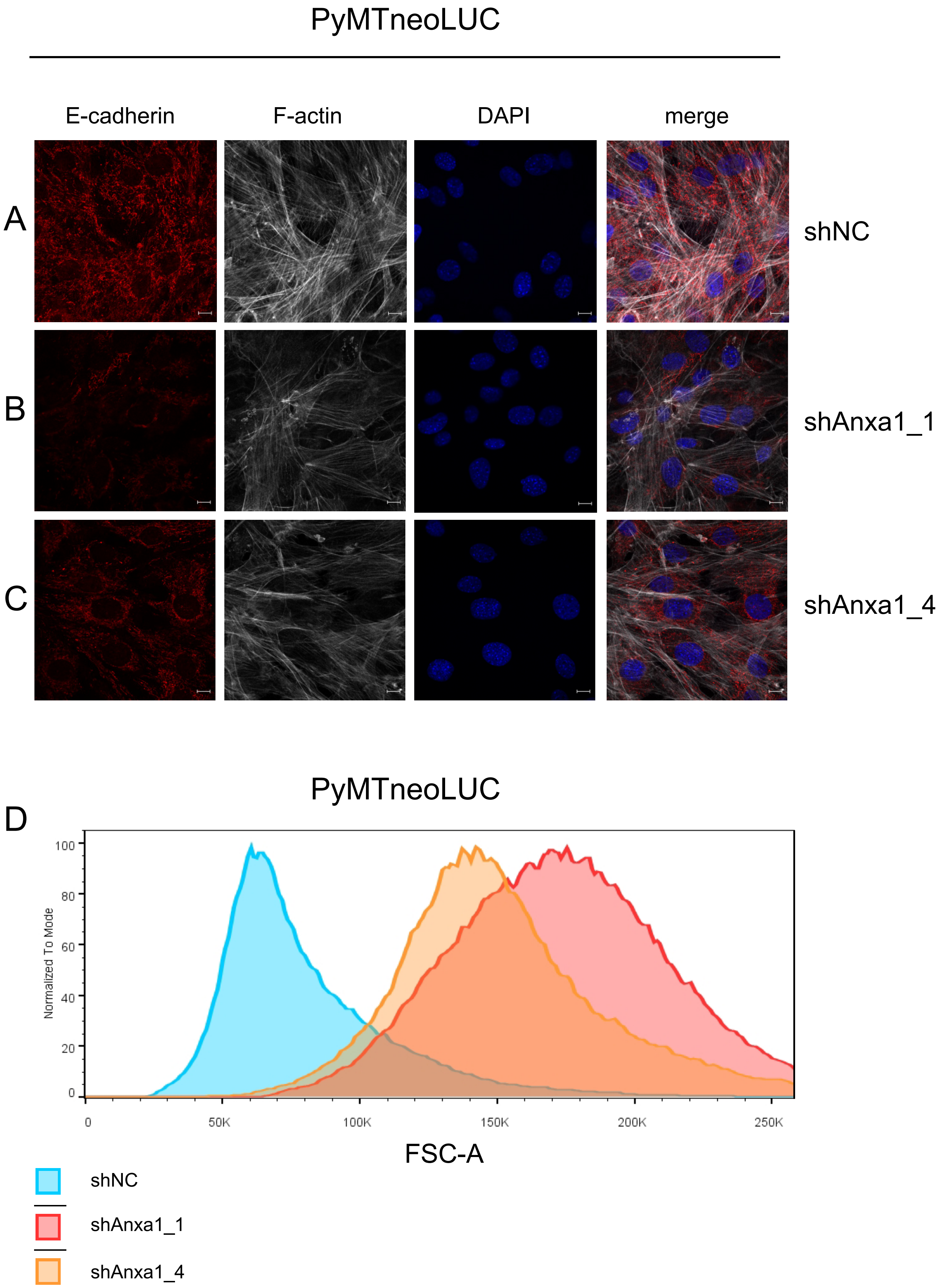
PyMTneoLUC
E-cadherin
F-actin
DAPI
merge
A
shNC
B
shAnxa1_1
C
shAnxa1_4
PyMTneoLUC
D
Normalized To Mode
100
80
60
40
20
0
0
50K
100K
150K
200K
250K
FSC-A
shNC
shAnxa1_1
shAnxa1_4

Figure S15

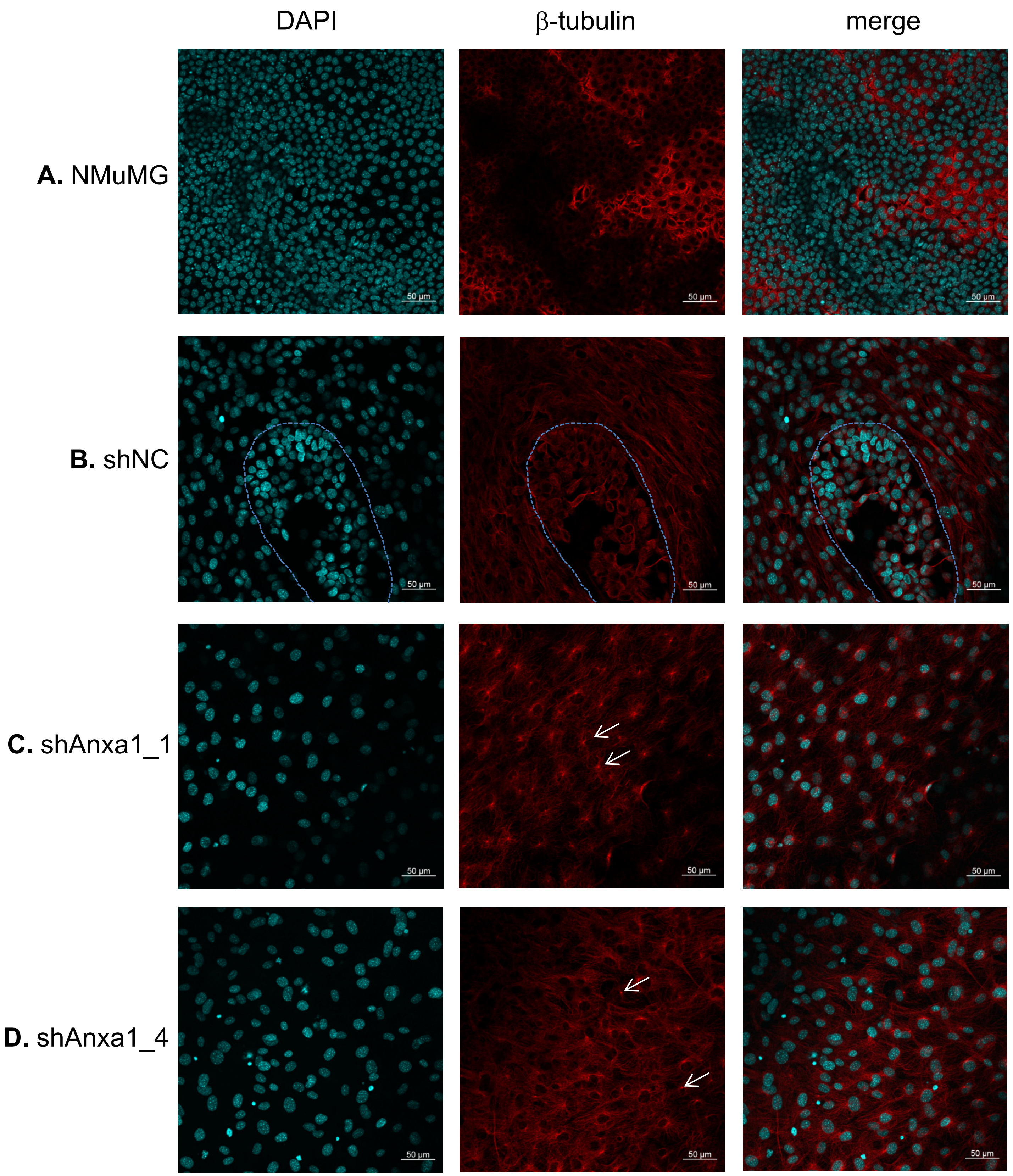

DAPI
β-tubulin
merge
A. NMuMG
B. shNC
C. shAnxa1_1
D. shAnxa1_4
50 μm

Figure S16

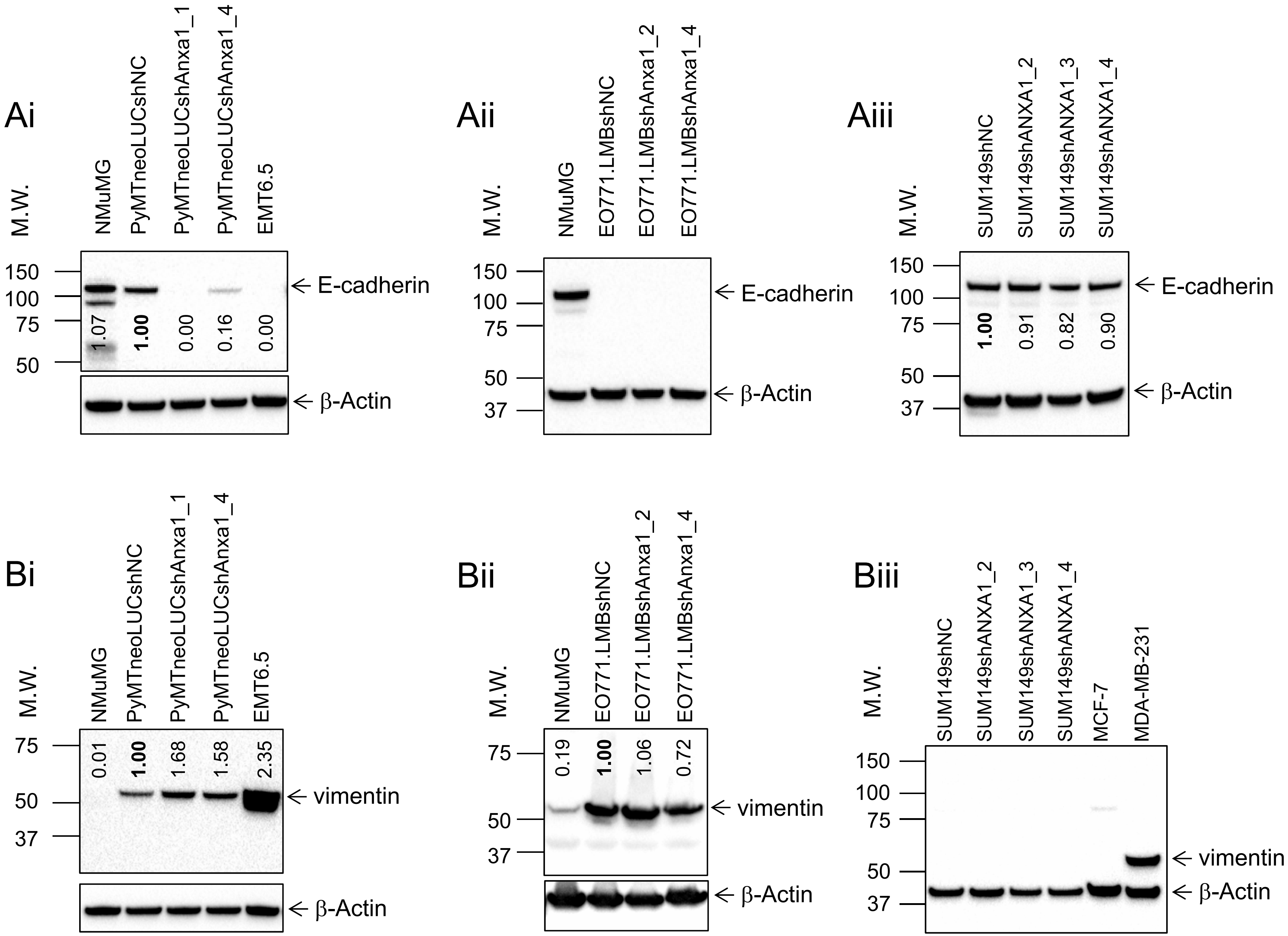

Ai
M.W.
NMuMG
PyMTneoLUCshNC
PyMTneoLUCshAnxa1_1
PyMTneoLUCshAnxa1_4
EMT6.5
150
100
75
50
1.07
1.00
0.00
0.16
0.00
E-cadherin
β-Actin
Aii
M.W.
NMuMG
EO771.LMBshNC
EO771.LMBshAnxa1_2
EO771.LMBshAnxa1_4
150
100
75
50
37
E-cadherin
β-Actin
Aiii
M.W.
SUM149shNC
SUM149shANXA1_2
SUM149shANXA1_3
SUM149shANXA1_4
150
100
75
50
37
1.00
0.91
0.82
0.90
E-cadherin
β-Actin
Bi
M.W.
NMuMG
PyMTneoLUCshNC
PyMTneoLUCshAnxa1_1
PyMTneoLUCshAnxa1_4
EMT6.5
75
50
37
0.01
1.00
1.68
1.58
2.35
vimentin
β-Actin
Bii
M.W.
NMuMG
EO771.LMBshNC
EO771.LMBshAnxa1_2
EO771.LMBshAnxa1_4
75
50
37
0.19
1.00
1.06
0.72
vimentin
β-Actin
Biii
M.W.
SUM149shNC
SUM149shANXA1_2
SUM149shANXA1_3
SUM149shANXA1_4
MCF-7
MDA-MB-231
150
100
75
50
37
vimentin
β-Actin

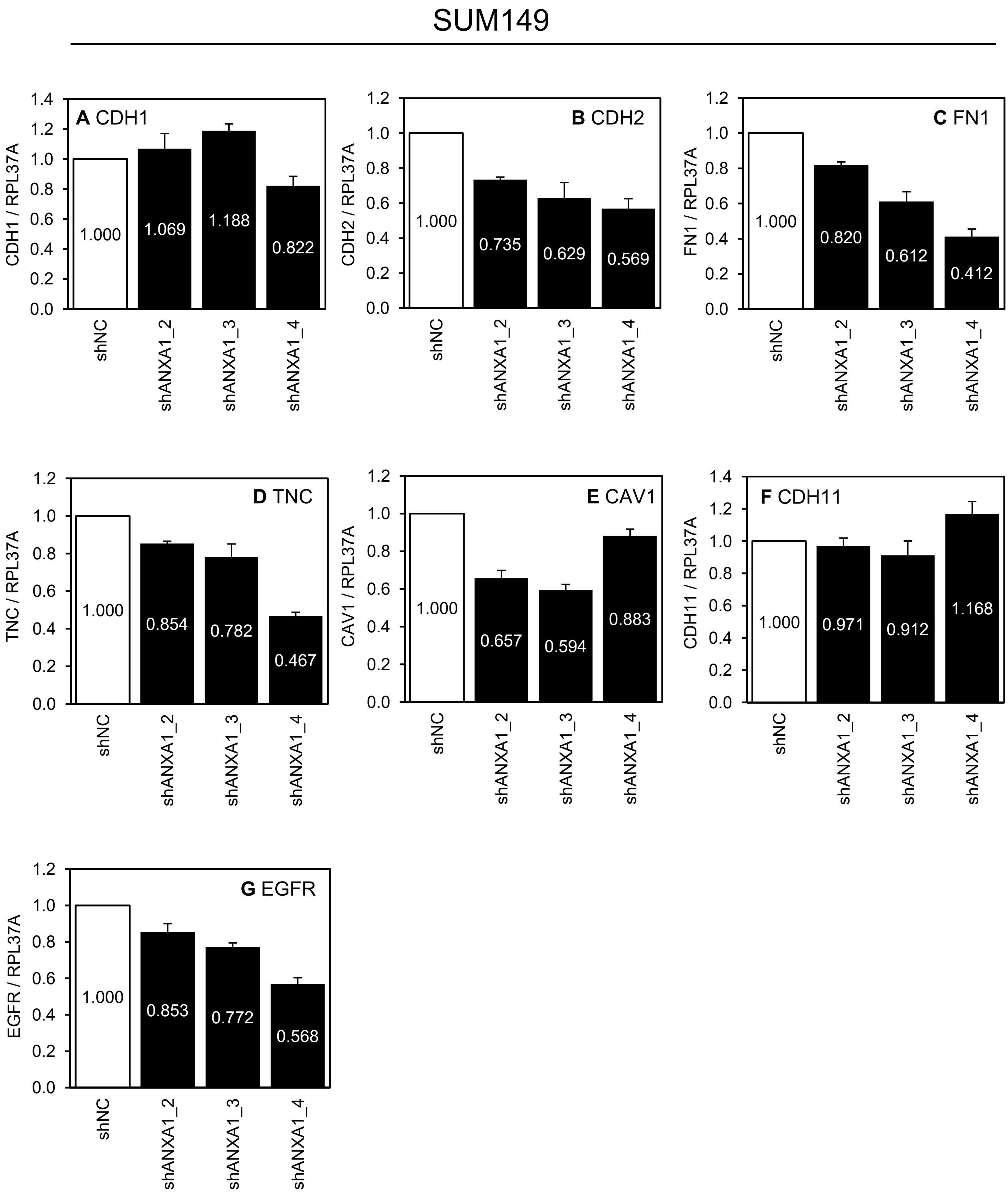
SUM149
A CDH1
CDH1 / RPL37A
1.000
1.069
1.188
0.822
B CDH2
CDH2 / RPL37A
1.000
0.735
0.629
0.569
C FN1
FN1 / RPL37A
1.000
0.820
0.612
0.412
D TNC
TNC / RPL37A
1.000
0.854
0.782
0.467
E CAV1
CAV1 / RPL37A
1.000
0.657
0.594
0.883
F CDH11
CDH11 / RPL37A
1.000
0.971
0.912
1.168
G EGFR
EGFR / RPL37A
1.000
0.853
0.772
0.568
shNC
shANXA1_2
shANXA1_3
shANXA1_4

Figure S17

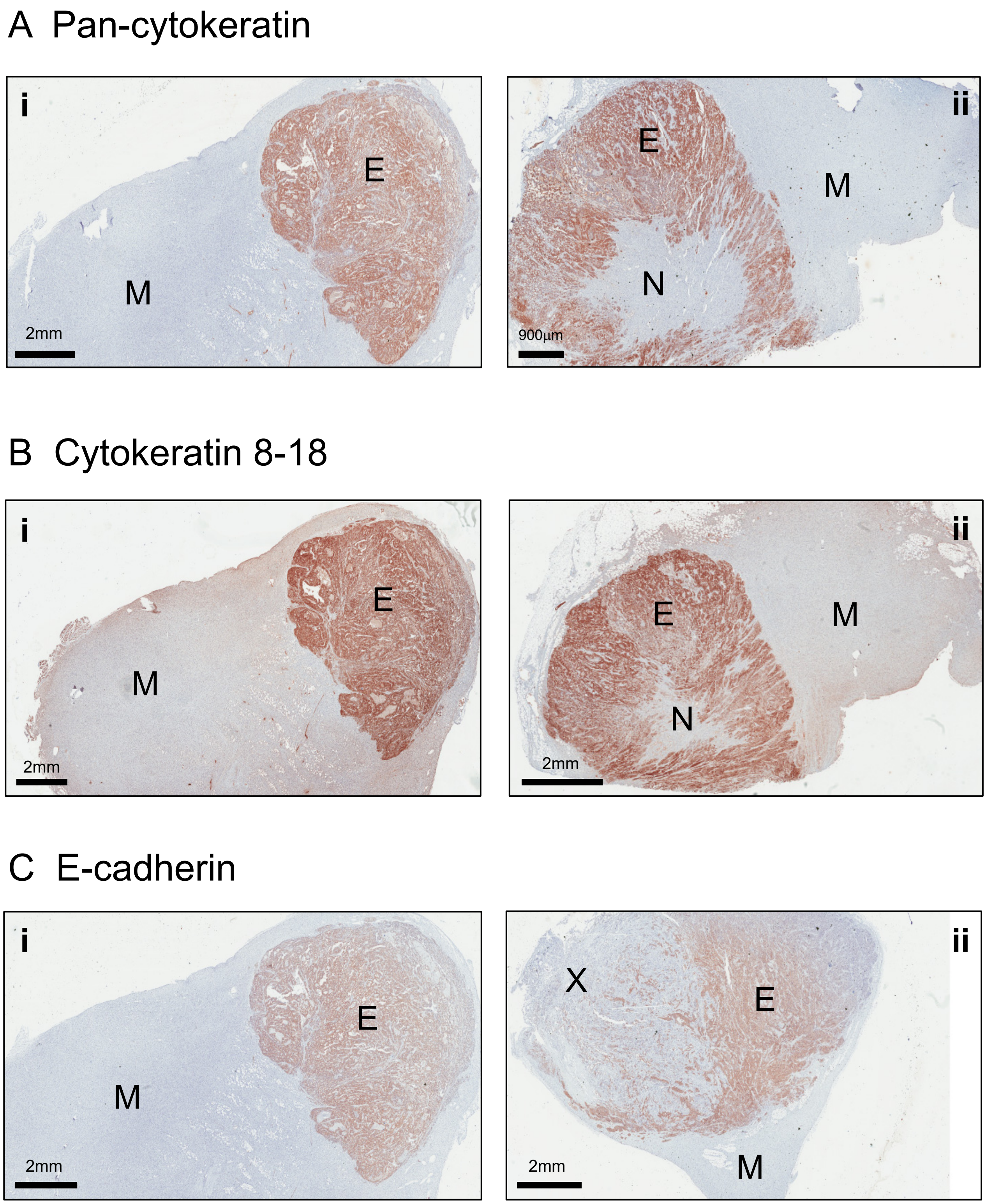
A Pan-cytokeratin
i
E
M
2mm
ii
E
M
N
900µm
B Cytokeratin 8-18
i
E
M
2mm
ii
E
M
N
2mm
C E-cadherin
i
E
M
2mm
ii
X
E
M
2mm

Figure S18

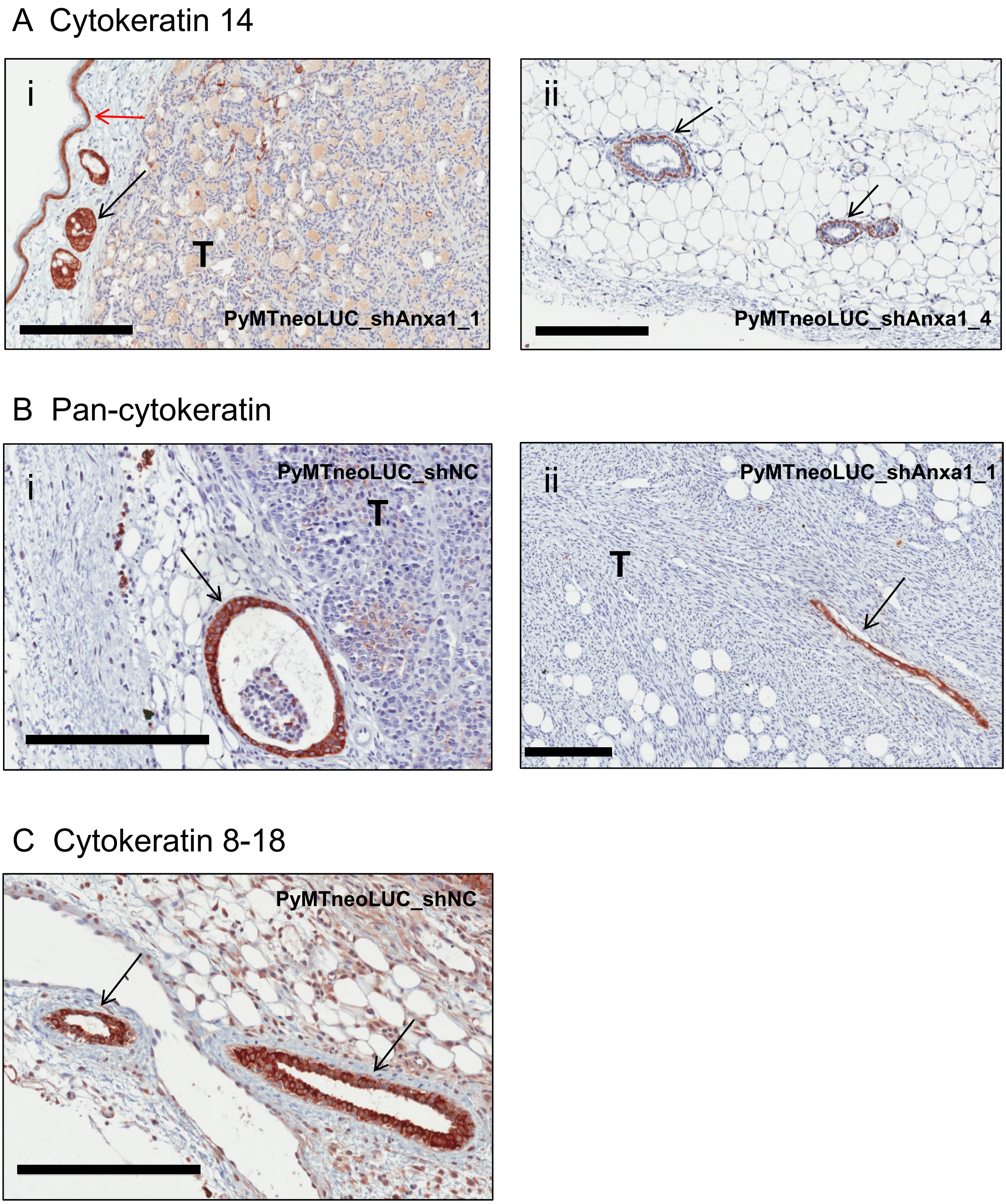
A Cytokeratin 14
i
T
PyMTneoLUC_shAnxa1_1
ii
PyMTneoLUC_shAnxa1_4
B Pan-cytokeratin
i
PyMTneoLUC_shNC
T
ii
PyMTneoLUC_shAnxa1_1
T
C Cytokeratin 8-18
PyMTneoLUC_shNC

Figure S19

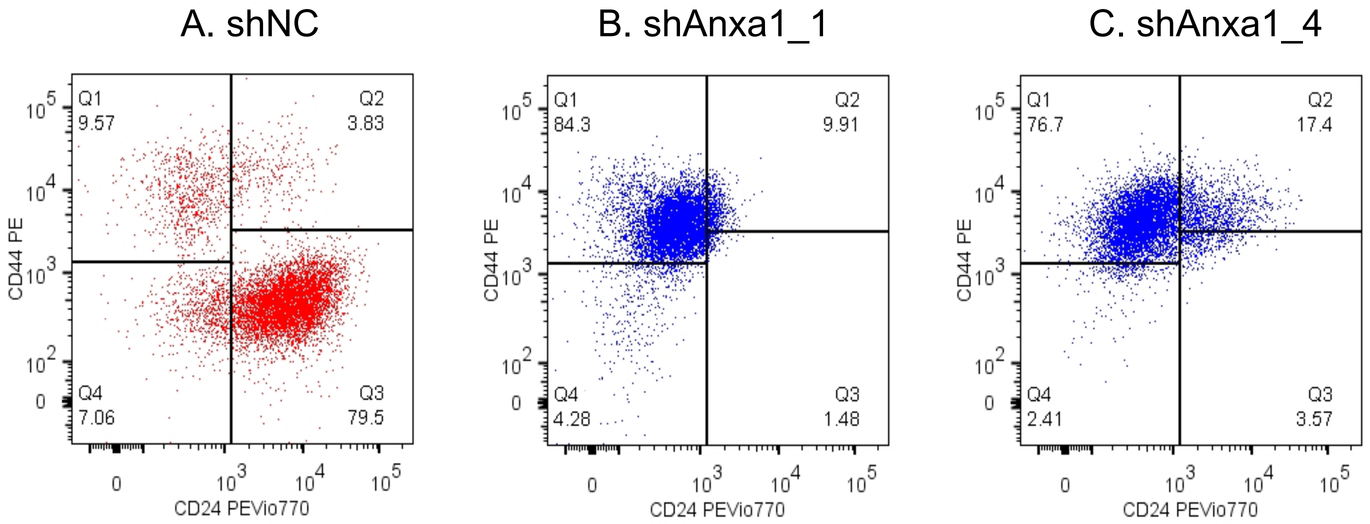

A. shNC
B. shAnxa1_1
C. shAnxa1_4
Q1
9.57
Q2
3.83
Q4
7.06
Q3
79.5
Q1
84.3
Q2
9.91
Q4
4.28
Q3
1.48
Q1
76.7
Q2
17.4
Q4
2.41
Q3
3.57
CD44 PE
CD24 PEVio770

Figure S20

Figure S21

Figure 2B

B

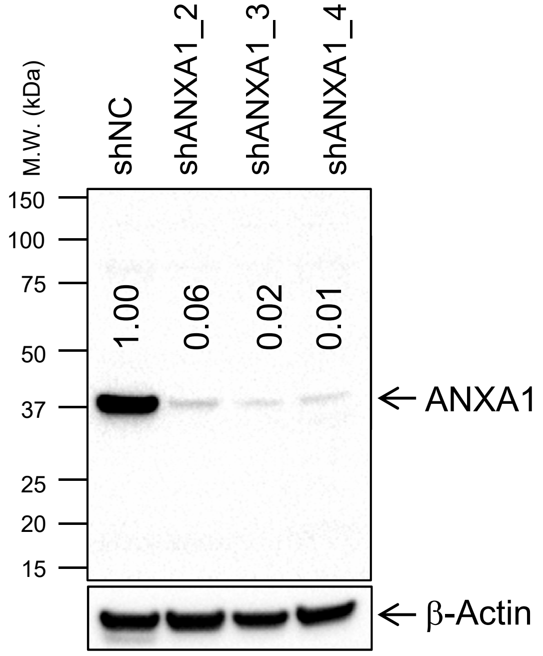

Original Western blot images for Figure 2B

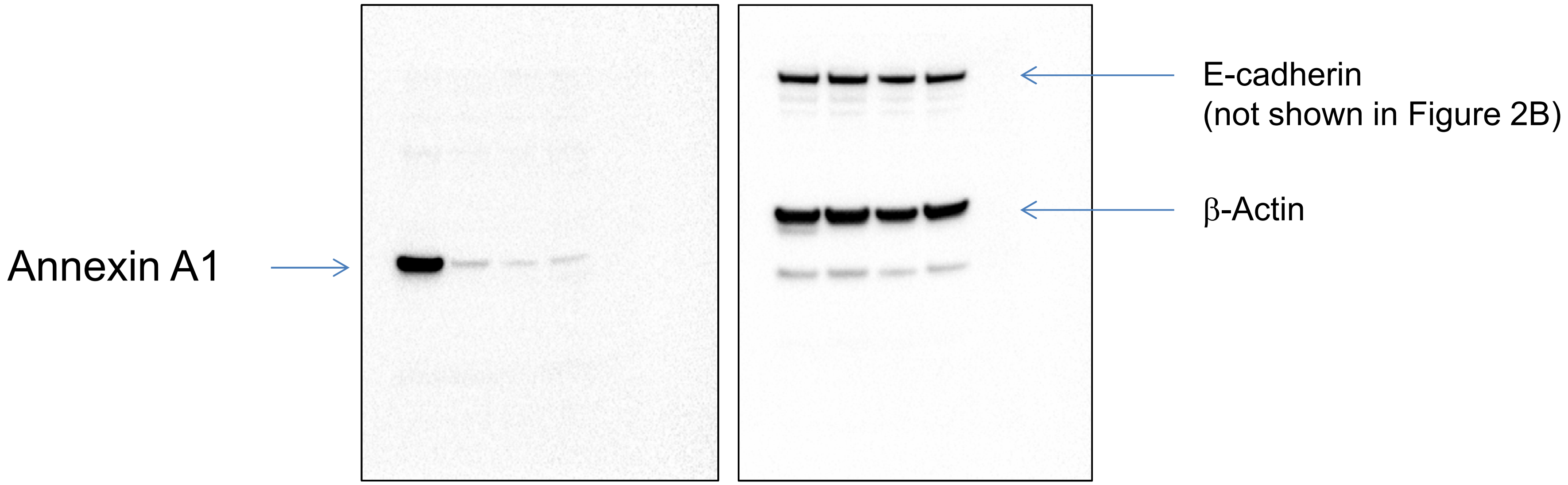

Figure S22

Figure S16

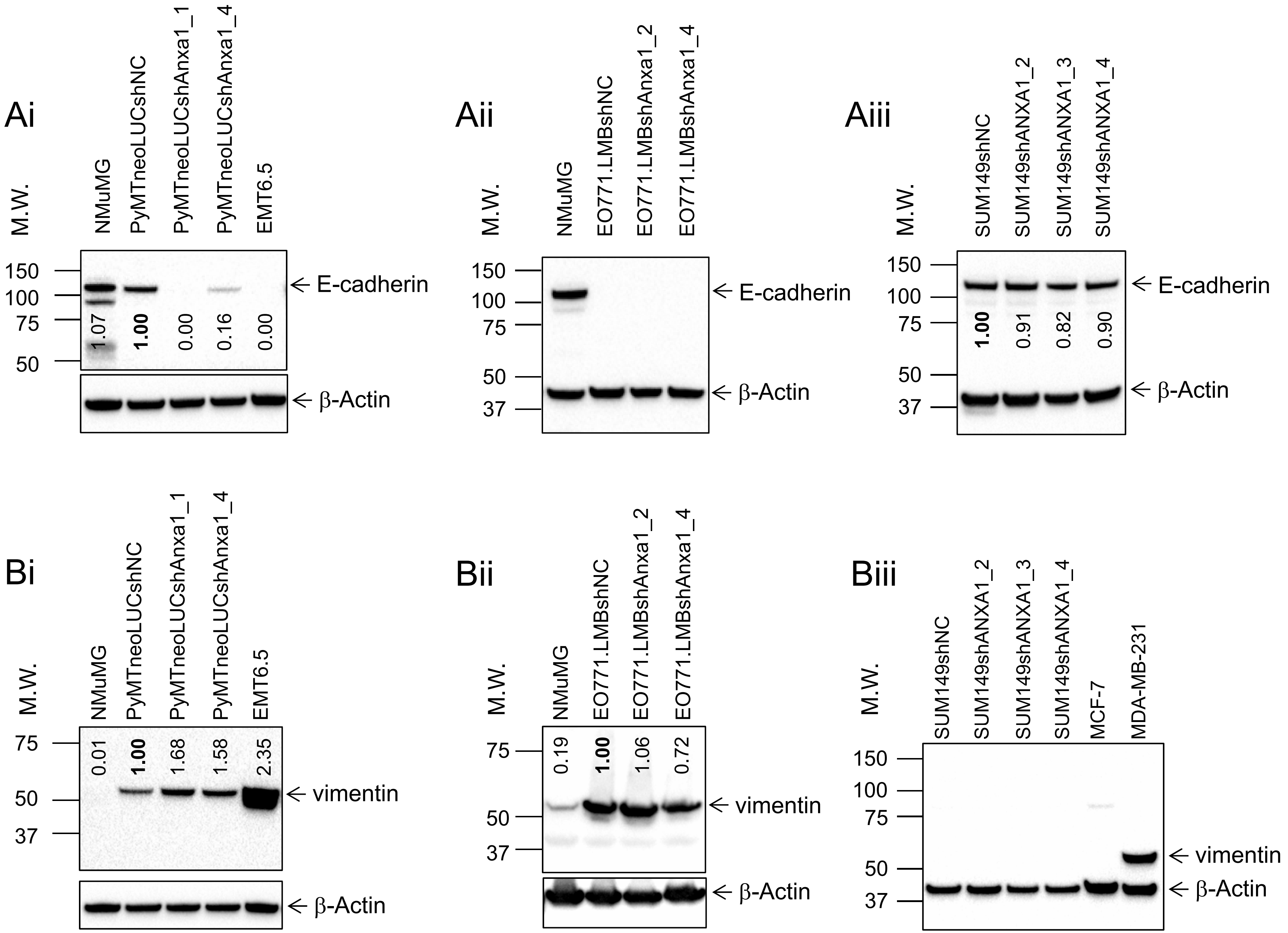

Figure S16Ai and S16Aii were from the same gel (below, .tiff files)

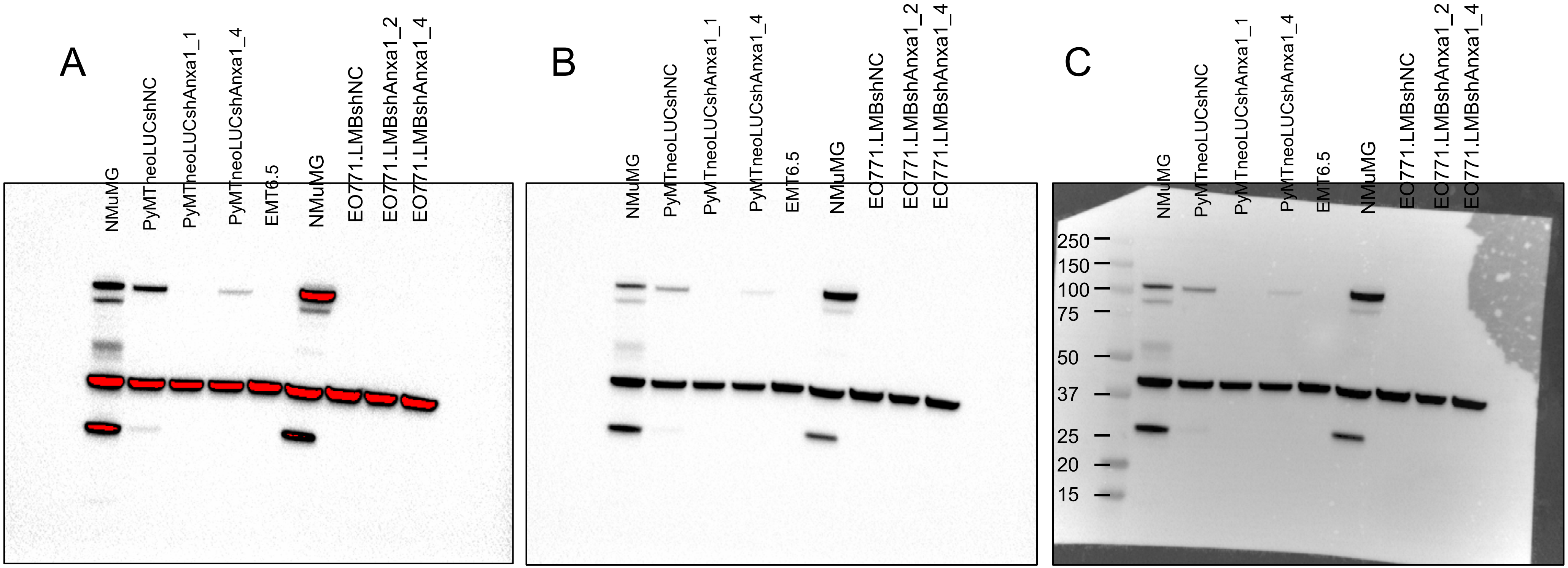

Long exposure showing saturated β-actin bands

This is the setting used for E-cadherin image and densitometry of E-cadherin in Ai

Short exposure

This is the setting used for E-cadherin image in Aii and image of β-Actin in Ai and Aii and densitometry of β-actin in Ai

Merged image

Merge of B and visible image of the blot membrane showing full MW markers (Precision Plus Bio-Rad)

Figure S16Aiii. Raw data .tiff files below

A

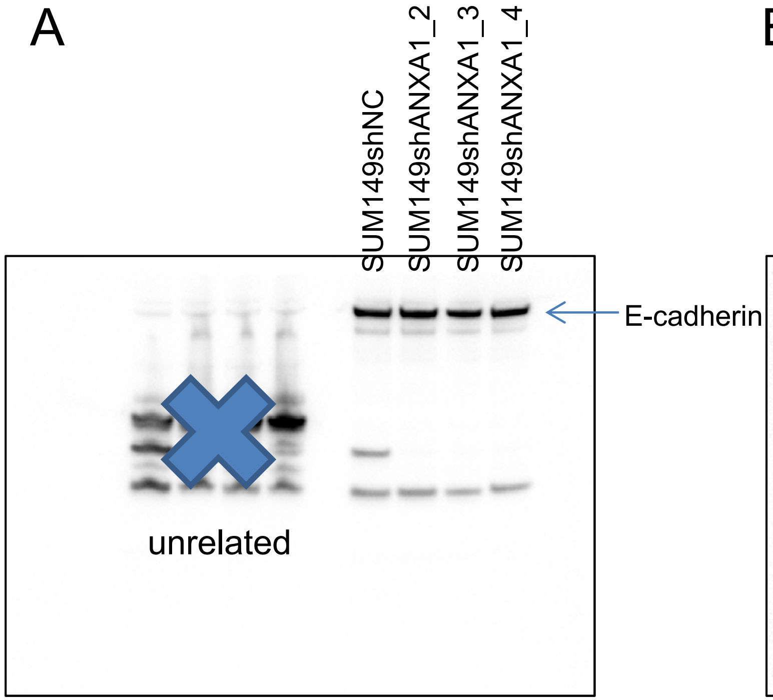

This is the setting used for densitometry of E-cadherin in Aiii

B

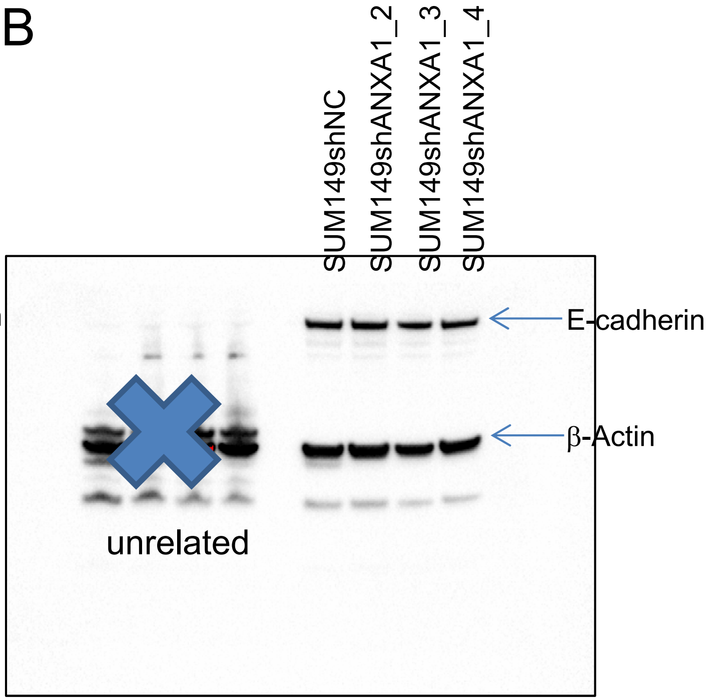

The blot was reprobed for β-actin and shows residual E-cadherin signal. This is the setting used for densitometry of β-Actin

C

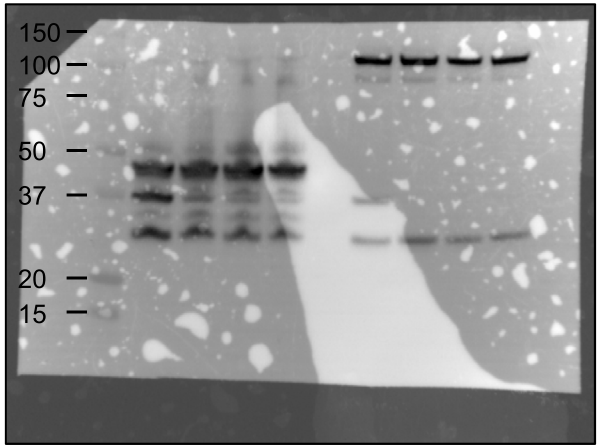

Merged image

Merge of **A** and visible image of the blot membrane showing full MW markers (Precision Plus Bio-Rad)

Figure S16Bi. Raw data .tiff files below. Blotted for Vimentin then reprobed for β-actin

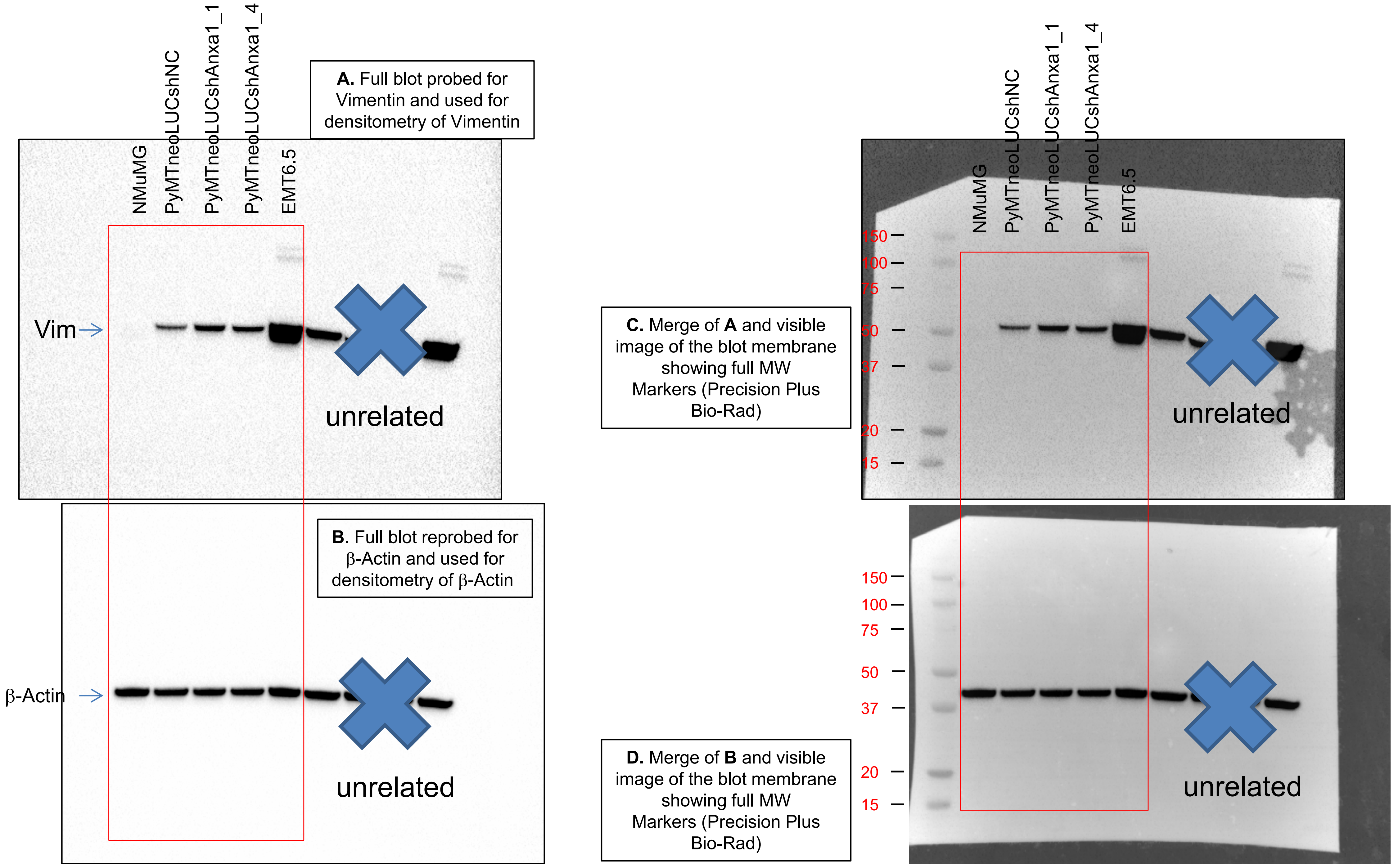

Figure S16Bii. Raw data .tiff files below

A. Full blot probed for Vimentin and used for densitometry of Vimentin

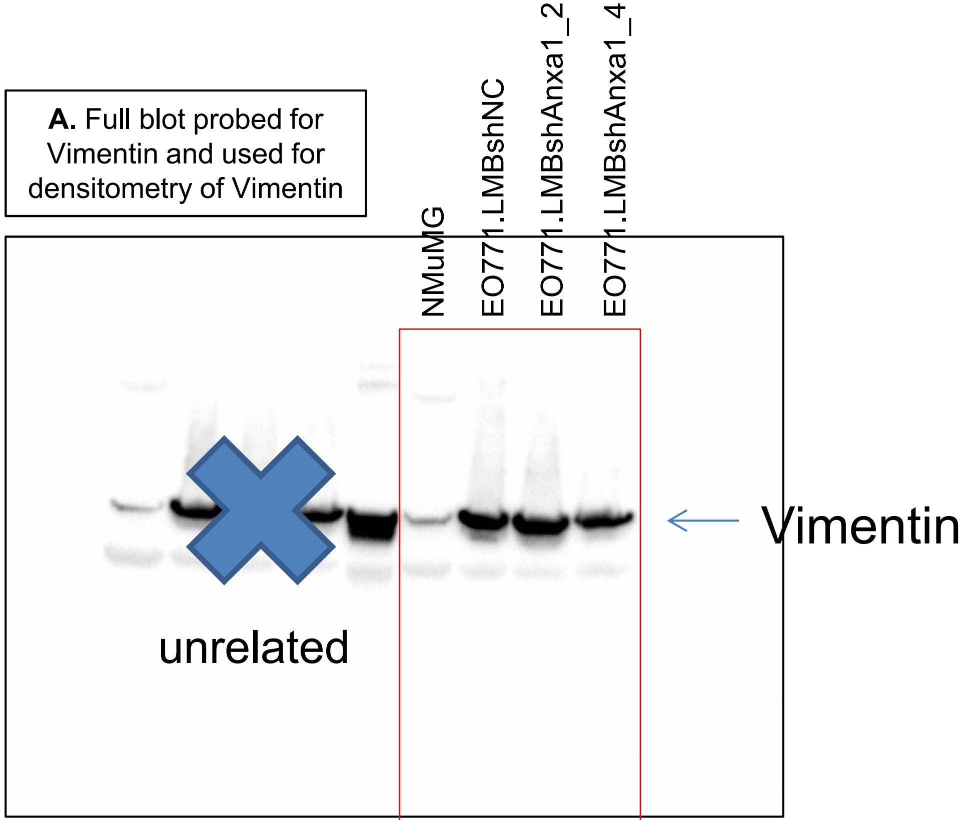

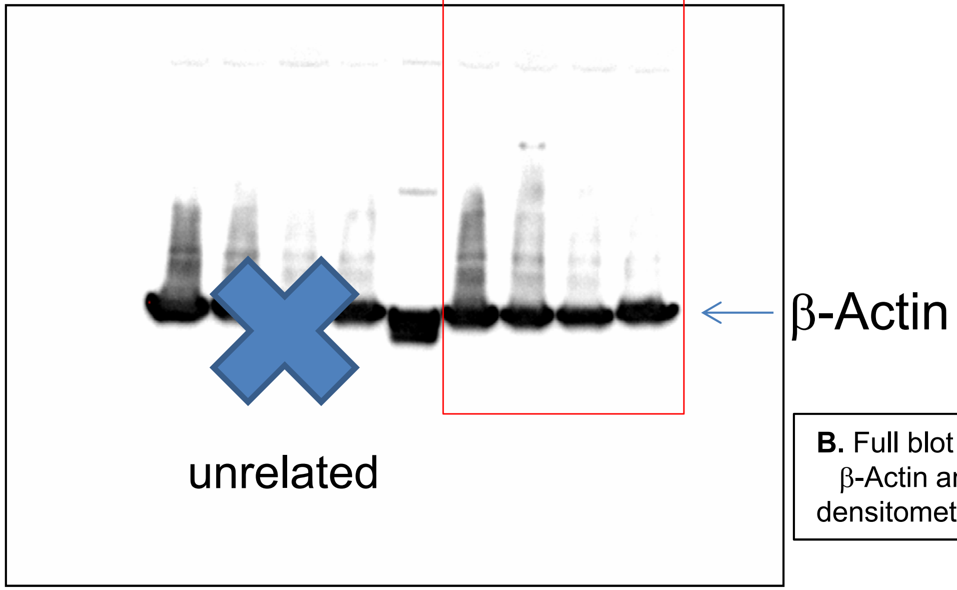

B. Full blot reprobed for β-Actin and used for densitometry of β-Actin

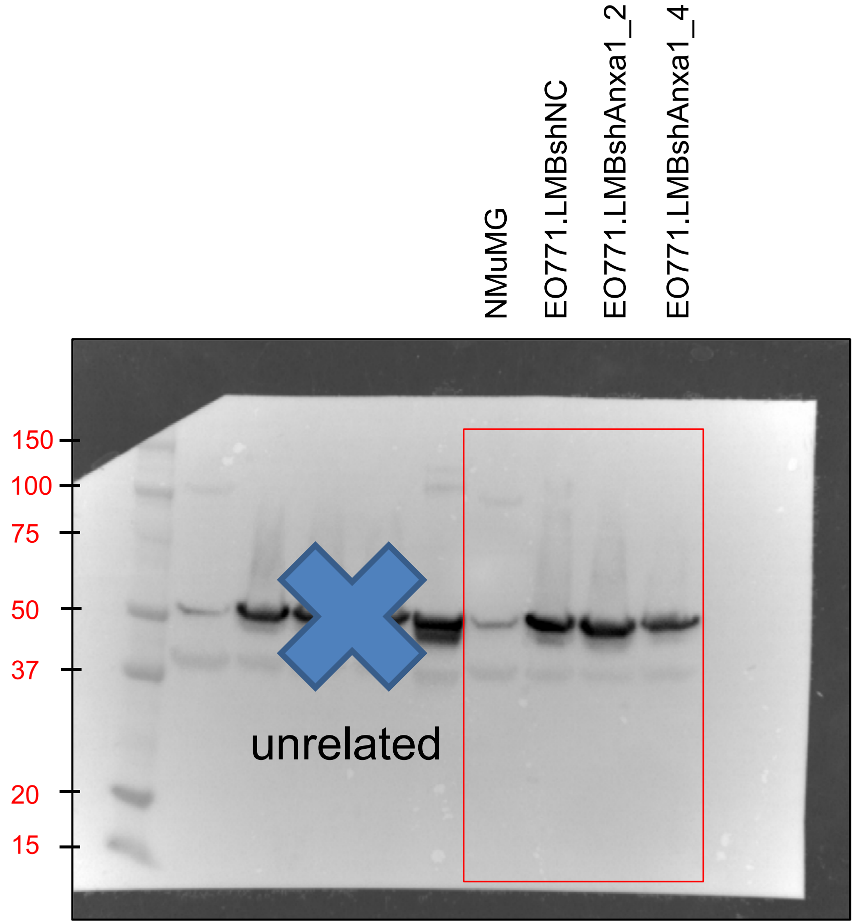

C. Merge of **A** and visible image of the blot membrane showing full MW Markers (Precision Plus Bio-Rad)

Figure S16Biii. Raw data .tiff files below

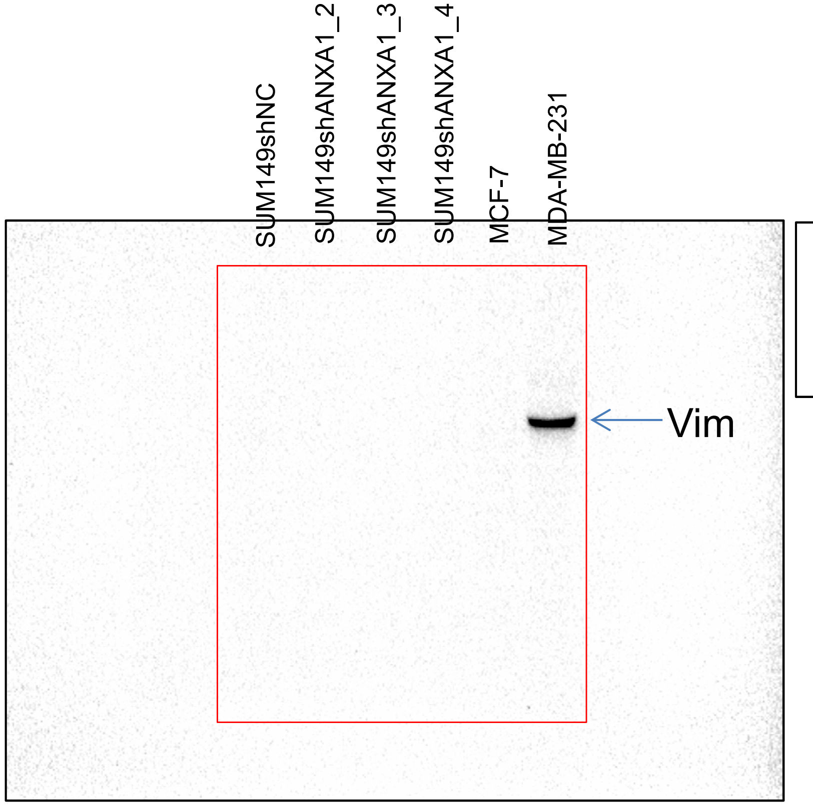

A. Full length blot probed for Vimentin showing Vimentin signal used for densitometry

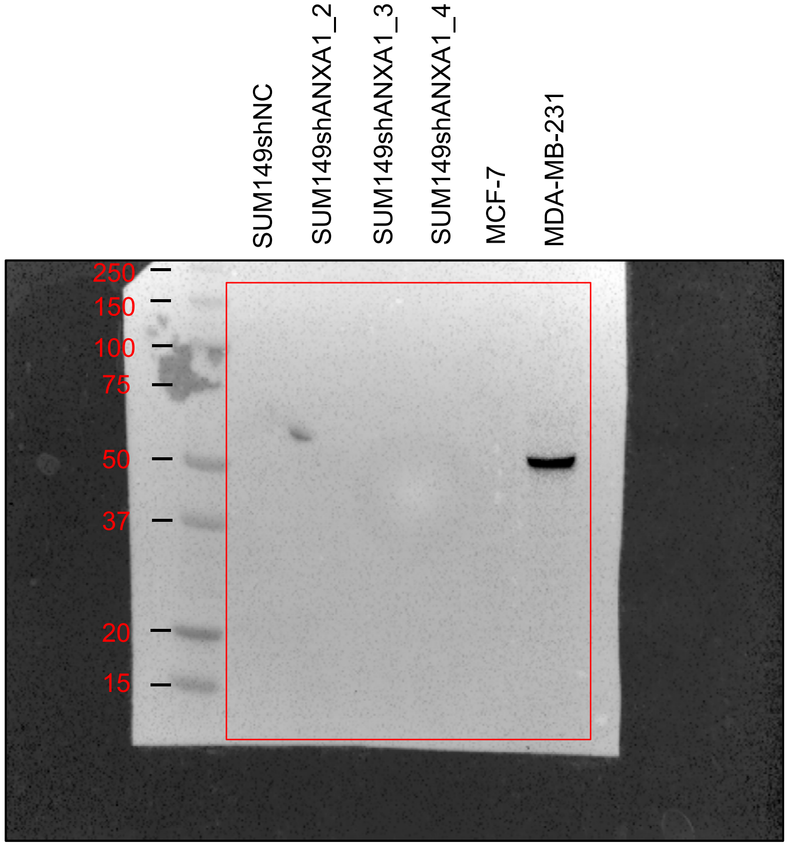

B. Merge of **A** and visible image of the blot membrane showing full MW Markers (Precision Plus Bio-Rad)

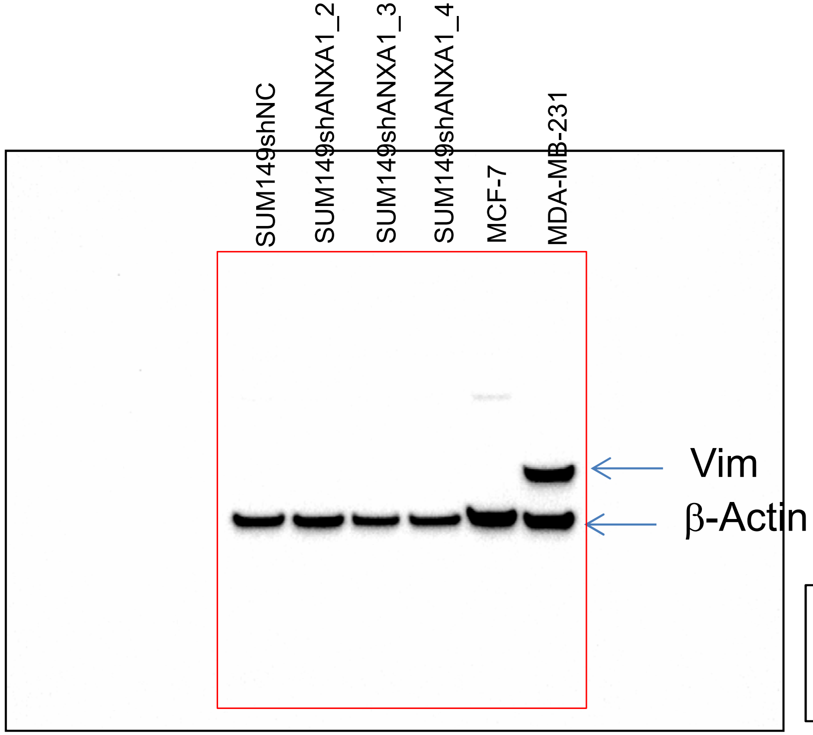

C. Full length blot probed for Vimentin and then reprobed for β-Actin showing β-Actin signal and also the residual Vimentin signal

Table S1: List of primer sequences for SYBR green qRT-PCR

Human	Forward	Reverse
Caveolin 1	CAG GGA CAT CTC TAC ACC	TCA AAG TCA ATC TTG ACC AC
**Mouse**	**Forward**	**Reverse**
E-cadherin	AGG CTG GCT GAA AGT GAC ACA	ACA CGG CAT GAG AAT AGA GGA TGT
Gap junction protein, alpha 1 (Gja1)	GGC GTG CCG GCT TCA CTT TCA T	AAG GCG CTC CAG TCA CCC ATG
Tight junction protein 1 (Tjp1)	GGA GCT ACG CTT GCC ACA CT	GTC AAT CAG GAC AGA AAC ACA GTT G
Desmoplakin	CCG GCT GGG ACG AGT TC	CTG TGG CTA TTG ATG TGC TGT TC
N-cadherin	GGT CTG TTC CAG AGG GAT CAA A	GGA TCA TCC GCA TCA ATG G
Fibronectin	GGC ATC GGG GAG TGG CAC TG	ATT GGG CTG GCT GGG GGT CT
Vimentin	AGG AAG CCG AAA GCA CCC TGC	CCG TTC AAG GTC AAG ACG TGC CA
Cytokeratin 8	TGA GGA TGA GAT CAA CAA GCG T	TGA TCT CGT CGG TCA GTC CT
Cytokeratin 18	CAA GTA TGA GAC AGA ACT AGC	GGA CTT CCT CTT CAT GAT TC

Supplementary Figure Legends

Figure S1: Expression of Annexin A1 mRNA from Affymetrix microarray analysis of human breast cancer cell lines. Luminal (n=29), basal A (n=10), basal B (n=15). Expression in MDA-MB-231 and SUM149 TNBC lines are indicated with an asterisk [1].

Figure S2: Annexin A1 expression is not dysregulated in models of metastatic breast cancer
Human Annexin A1 (**A**) and FPR1 (**B**) expression in RNA-Seq data from isolated MDA-MB-231-derived primary tumour cells grown as orthotopic xenografts in immuno-deficient mice [2]. Mean +/- SEM. 231_ATCC (non-metastatic, n=2), 231_LNA (metastatic, n=6), 231_LM2 (metastatic, n=6), 231_HM.LNm5 (metastatic, n=6). FPR1 was differentially expressed across the four tumour models (one-way ANOVA P = 0.0003), so expression in each metastatic model was compared to non-metastatic 231_ATCC tumours (Dunnett's multiple comparisons test). FPR1 mRNA levels were lower in 231_LNA (***, P < 0.001), 231_LM2 (**, P < 0.01), and 231_HM.LNm5 (****, P < 0.0001) tumours. Mouse Annexin A1 (**C**) and Fpr1 (**D**) expression in whole primary tumours from the indicated allograft models (n=3 per line). Mean +/- SEM. Fpr1 was differentially expressed across the five tumour models (one-way ANOVA P = 0.0005), so expression in each metastatic model (4T1.2 and EO771.LMB) was compared to expression in the corresponding non-metastatic isogenic line (67NR and EO771, respectively). Fpr1 mRNA levels were lower in 4T1.2 than in 67NR (**, P < 0.01. Tukey's multiple comparisons test).

Figure S3: Annexin A1 depletion does not alter primary tumour growth or spontaneous metastasis of MDA-MB-231_HM orthotopic xenografts
A. TaqMan qRT-PCR analysis of Annexin A1 mRNA expression in control (MDA-MB-231_HM.LNm5_**shNC**) and Annexin A1 knockdown MDA-MB-231_HM.LNm5 (MDA-MB-231_HM_**shANXA1_1**, MDA-MB-231_HM_**shANXA1_2**, and MDA-MB-231_HM_**shANXA1_4**) cell lines. Annexin A1 expression was normalised to that of 18S rRNA and expression in the control MDA-MB-231_HM_shNC line was set to 1. Mean +/- SD (n=3) **B.** Western blot analysis of Annexin A1 protein expression in the indicated MDA-MB-231_HM.LNm5 stable transfectants. Cells were grown continuously in normal medium (10% FBS, +) or serum starved for 24h (-). **C.** Stable MDA-MB-

231_HM.LNm5 cell lines were orthotopically implanted into immuno-deficient mice. Primary tumour growth rates were assessed by calculating tumour volumes (mm^3). using electronic calliper measurement. All primary tumours were surgically resected (R) at day 21 when around $300mm^3$ (0.3g) in size. Growth rates were compared to the controls using mixed effects linear regression modelling [2-4]. MDA-MB-231_HM_shNC (n=6) v MDA-MB-231_HM_shANXA1_1 (n=6), P = 0.019. MDA-MB-231_HM_shNC v MDA-MB-231_HM_shANXA1_2 (n=7), P = 0.254. MDA-MB-231_HM_shNC v MDA-MB-231_HM_shANXA1_4 (n=6), P = 0.791. **D.** TaqMan qRT-PCR analysis of Annexin A1 mRNA expression in resected primary tumours from the indicated tumour lines. Expression was normalised to RPL37A expression. The linearised ΔC_T value (Annexin A1 - RPL37A) was plotted. Mean +/- SEM (n=3 tumours per group). One-way ANOVA $P < 0.0001$. MDA-MB-231_HM_shNC v MDA-MB-231_HM_shANXA1_1, **** $P < 0.0001$. MDA-MB-231_HM_shNC v MDA-MB-231_HM_shANXA1_4, **** $P < 0.0001$ (Dunnett's multiple comparisons test). **E.** Representative images of resected primary control (shNC) and Annexin A1 knockdown MDA-MB-231_HM.LNm5 tumours. Brightfield images (left), and turboGFP (middle) and tdTomato (right) fluorescent images are shown. Original magnification (x7). **F.** Mass of axillary lymph node metastases. Mean (g) +/- SEM. Six mice were analysed per group, except MDA-MB-231_HM_shANXA1_2 (n=7). One-way ANOVA $P > 0.05$. **G.** Analysis of lung metastatic burden by genomic TaqMan qPCR. Mean +/- SEM is shown. A trend towards higher lung metastasis in Annexin A1 depleted tumour lines was observed (one-way ANOVA P = 0.0336). However, all individual comparisons with the control MDA-MB-231_HM_sNC line were not significant (ns, $P > 0.05$, Dunnett's multiple comparisons test). Six mice were analysed per group, except MDA-MB-231_HM_shANXA1_2 (n=7).

Figure S4: Immunofluorescence analysis of Annexin A1 expression in SUM149 cells

Cultured SUM149_shNC (**A**), SUM149_shANXA1_3 (**B**), and SUM149_shANXA1_4 (**C**) cell lines were stained with anti-Annexin A1 antibodies (mouse mAb clone EH17A, Santa Cruz Biotechnology) and DAPI to visualize nuclei. Images were generated by confocal microscopy (ZEISS LSM780, Carl Zeiss, North Ryde, Australia). Annexin A1 (left panels) and merged DAPI/Annexin A1 images (right panels) are shown. White arrows indicate plasma membane-associated Annexin A1 protein in SUM149_shNC cells (**A**). Scale bars represent 20μm.

Figure S5: Immunohistochemical analysis of protein expression in SUM149_shNC and SUM149_shANXA1 primary mammary xenografts

A. Annexin A1 (mouse mAb MRQ-3). **B.** Epithelial marker: luminal cytokeratin 8-18 (CK8-18). Arrows point to CK8-18 positive epithelial cells in normal mouse mammary ducts. **C.** Epithelial marker: E-cadherin. Arrows point to E-cadherin positive epithelial cells in normal mouse mammary ducts. **D.** Mesenchymal marker: vimentin intermediate filament protein. **E.** Mouse endothelial cell marker: CD34. CD34 staining was used to determine tumour micro vessel density. No differences were observed among the tumour lines (data not shown). Scale bars represent 200μm.

Figure S6: Expression of Annexin A1 in mouse mammary tumour lines

A. Details of mouse mammary tumour cell lines used in this publication. The Table shows the mouse strain and whether the tumour arose spontaneously or from an MMTV-driven genetically engineered mouse model (GEMM). The oncogenic driver is indicated in brackets. The Table also shows whether the cell line has spontaneous metastatic potential when implanted into syngeneic hosts. * HB158+ and MH248 cell lines are not metastatic when implanted into immunodeficient hosts [5]. **B.** TaqMan qRT-PCR analysis of Annexin A1 mRNA expression in NMuMG immortal mouse mammary epithelial cells [6], and in a panel of mouse mammary tumour cell lines, J774 mouse monocytes [7], RAW264.7 mouse macrophages [8], and B16.F10 mouse melanoma cells [9]. Gene expression was normalised to Rps27 internal control and expression in NMuMG was set to 1. Mean +/- SD (n=3). **C.** Western blot analysis of Annexin A1 protein expression in a panel of mouse mammary tumour cell lines. **D.** TaqMan qRT-PCR analysis of alpha smooth muscle actin (αSma) mRNA expression in a panel of mouse mammary cancer cell lines. αSma expression was normalised to Rps27 internal control and expression in NMuMG was set to 1. Mean +/- SD (n=3).

Figure S7: Expression of estrogen receptor alpha (ERα), progesterone receptor (PR), and erb-b2 in spontaneous and transgenic allograft mouse mammary tumour models

Ai: PyMT, AT-3, EO771.LMB and EMT6.5 mouse tumour lines (see Figure S6A) were established in the 4th inguinal mammary gland of female mice, dissected, fixed in formalin and then stained for ERα protein expression (mouse mAb clone 6F11, 1:50 dilution, Abcam). Normal mammary gland adjacent to an EMT6.5 tumour (right panel, control) served as a positive control for ERα expression. Arrows

point to ERα positive epithelial cell nuclei in mammary ducts. Scale bars represent 200μM. Each of the four models were negative for nuclear ERα protein expression. **Aii.** TaqMan qRT-PCR analysis of ERα mRNA expression (Assay ID: Mm00433149_m1) in mouse mammary tumour lines cultured *in vitro*. Expression in NMuMG immortal mouse mammary epithelial cells was set to 1. Mean +/- SD (n=3). The *Pik3ca* mutant MH248 line was used as a positive control for ERα mRNA expression [5]. **Bi:** Mammary tumour sections were stained for erb-b2 protein expression (mouse mAb clone 3B5, 1:40 dilution, Merck Millipore). A tumour established from the Neu-positive mouse mammary tumour line H2N100 (right panel, control) was used as a positive control for erb-b2 expression [10]. An erb-b2 negative normal mammary duct within an EMT6.5 tumour is indicated with an arrow. Scale bars represent 200μM. Each of the four models were negative for erb-b2 protein expression. **Bii.** TaqMan qRT-PCR analysis of erb-b2 mRNA in mouse mammary tumour lines cultured *in vitro*. qRT-PCR assays that detect either mouse erb-b2 only (Assay ID: Hs01001580_m1, left bars, blue) or both mouse erb-b2 and rat Neu (Assay ID: Hs01001599_m1, right bars, black) were conducted. For mouse erb-b2 alone, expression in NMuMG cells was set to 1. For mouse erb-b2 plus rat Neu, PCR amplification was only detected in rat Neu-expressing H2N100 mouse mammary tumour cells, which were used as a positive control and set to 100. Mean +/- SD (n=3). **C.** TaqMan qRT-PCR analysis of PR mRNA expression (Assay ID: Mm00435628_m1) in mouse mammary tumour lines cultured *in vitro*. Expression in NMuMG cells was set to 1. Mean +/- SD (n=3). The *Pik3ca* mutant HB158+ line was used as a positive control for PR mRNA expression [5].

Figure S8: Knockdown of Annexin A1 in PyMTneoLUC and EO771.LMB mouse mammary tumour lines

Annexin A1 mRNA levels were measured in control (shNC) and two different Annexin A1 depleted (shAnxa1) lines in each of PyMTneoLUC (**Ai**) and EO771.LMB (**Bi**) cell lines. Annexin A1 expression was normalised to Rps27 internal control and expression in shNC cells set to 1. Mean +/- SD (n=3). Annexin A1 protein expression was assessed by Western blot analysis in the same PyMTneoLUC (**Aii**) and EO771.LMB (**Bii**) stable transfectants. NMuMG immortal mammary epithelial cells were used as a positive control for Annexin A1 expression in **Bii**. Expression was quantified using NIH Image J software [11], and normalised to β-actin protein levels. Expression was set to 1 in control shNC cells.

Figure S9: Growth of control and Annexin A1 depleted PyMTneoLUC mammary tumours in wild-type C57BL/6 mice

A. Mass of dissected primary tumours from **Figure 2A.** Mean +/- SEM. PyMTneoLUC_shNC, (n=4 of 7 total) at day 40. PyMTneoLUC_shAnxa1_1, n=5 (day 133). **B-C.** Representative brightfield (upper panel) and matched fluorescent (eGFP, lower panel) images of dissected primary mammary allografts. **B.** PyMTneoLUC_shNC (n=4 of 7 total), day 40. **C.** PyMTneoLUC_shAnxa1_1 (n=4 of 5 total), day 133. Magnification (x7).

Figure S10: Optical imaging of control and Annexin A1 depleted PyMTneoLUC mammary tumours in wild-type C57BL/6 mice

A. Bioluminescence images of mice bearing PyMTneoLUC_shAnxa1_1 or PyMTneoLUC_shAnxa1_4 mammary allografts (n=3 per group from a total 4 mice per group) on days 10, 31, and 50 after inoculation. The colour bar on the right indicates radiance ($p/s/cm^2/sr \times 10^6$). Minimum 1.25×10^5, maximum 1.25×10^6**.** **B.** Representative brightfield (left panels) and matched fluorescent (eGFP, right panels) *ex vivo* images of mammary tumours formed by the indicated PyMTneoLUC transfectants (from **Figure 2B-2C**). (n=3 per group is shown from a total of 4 per group). The day of harvest for each group is indicated on the right. Magnification (x7).

Figure S11: Optical imaging of control and Annexin A1 depleted PyMTneoLUC mammary tumours in immuno-deficient C57BL/6 mice

A. Representative bioluminescence images of C57BL/6NTac;B10(Cg)-$Rag^{2tm1Fwa}Il2rg^{tm1Wjl}$ immuno-deficient mice bearing PyMTneoLUC_shAnxa1_1 or PyMTneoLUC_shAnxa1_4 orthotopic tumours (n=3 from n=5 per group total) at days 20, 27 and 41. The colour bar on the right indicates radiance ($p/s/cm^2/sr \times 10^7$). Minimum 2×10^6, maximum 2×10^7. **B.** Mass of primary tumours formed in C57BL/6NTac;B10(Cg)-$Rag^{2tm1Fwa}Il2rg^{tm1Wjl}$ immuno-deficient mice (**from Figure 2D-2E**). Mean +/- SEM. Control PyMTneoLUC_shNC (n=4 of 5 total), PyMTneoLUC_shAnxa1_1 (n=5), PyMTneoLUC_shAnxa1_4 (n=5). The different groups were culled at different times as indicated. **C.** Representative brightfield (left panels) and matched fluorescent (eGFP, right panels) *ex vivo* images of mammary tumours formed in C57BL/6NTac;B10(Cg)-$Rag^{2tm1Fwa}Il2rg^{tm1Wjl}$ immuno-deficient mice by

the indicated PyMTneoLUC transfectants (from **Figure 2D-2E**). (n=3 per group is shown from a total of 5 per group). Magnification (x7).

Figure S12: Evaluation of EO771.LMB_shNC and EO771.LMB_shAnxa1_4 cell lines *in vivo*

A. Matched phase contrast (left side) and fluorescent (eGFP, right side) images of cultured EO771.LMB_shNC and EO771.LMB_shAnxa1_4 cell lines. Magnification (x100). **B-E.** Cells were orthotopically inoculated into syngeneic C57BL/6 mice and tumour growth measured for 25 days using electronic callipers, followed by surgical resection of the primary tumours. Mean tumour volume +/- SEM is plotted versus time. Growth rates were compared using mixed effects linear regression modelling [2, 3]. **B.** Wild-type (WT) C57BL/6 mice. EO771.LMB_shNC (n=10) v EO771.LMB_shAnxa1_4 (n=8). $P > 0.05$. **C.** *Anxa1*$^{-/-}$ C57BL/6 mice. EO771.LMB_shNC (n=6) v EO771.LMB_shAnxa1_4 (n=7). $P > 0.05$. **D.** *Fpr2*$^{-/-}$ C57BL/6 mice. EO771.LMB_shNC (n=6) v EO771.LMB_shAnxa1_4 (n=7). $P > 0.05$. **E.** *Ex vivo* images of representative EO771.LMB derived tumours grown in WT mice. EO771.LMB_shNC (top row). EO771.LMB_shAnxa1_4 (bottom row). **i.** Brightfield. **ii.** mCherry fluorescence (red). **iii.** eGFP fluorescence (green). Magnification (x7). **F.** Mass of resected primary mammary tumours at day 25. The mouse genotype and tumour cell line identity is indicated as is the sample size (n) per group. Mean +/- SEM. One-way ANOVA $P > 0.05$. **G.** Comparison of control EO771.LMB_shNC (shNC) growth rates in wild-type (WT), *Anxa1*$^{-/-}$, and *Fpr2*$^{-/-}$ C57BL/6 mice $P > 0.05$.

Figure S13: *In vitro* proliferation rates of control and Annexin A1 depleted PyMTneoLUC cells

2D proliferation assays were conducted over 3 days for the indicated PyMTneoLUC transfectants. Mean +/- SEM (n=8). No differences in 2D growth rates were observed among EO771.LMB_shNC, EO771.LMB_shAnxa1_2, and EO771.LMB_shAnxa1_4 cell lines (data not shown).

Figure S14: Immunofluorescence analysis of E-cadherin protein and filamentous actin (F-actin) in confluent cultures of PyMTneoLUC stable transfectants

A. PyMTneoLUC_shNC, **B.** PyMTneoLUC_shAnxa1_1, **C.** PyMTneoLUC_shAnxa1_4. Cells were stained for E-cadherin (red), F-actin (phalloidin, white), and nuclei (DAPI, blue). The merged fluorescent images are also shown. E-cadherin was cytoplasmic in PyMTneoLUC_shNC cells. No

differences in F-actin distribution were seen. Scale bars are 10μm. **D.** Analysis of cell size by flow cytometry. The indicated PyMTneoLUC stable transfectants were analysed using a FACS Aria III (BD Biosciences), and gated for single cells and viability (Sytox Blue). Forward SCatter-Area (FSC-A) was used as an indicator of cell volume.

Figure S15: The microtubule network as assessed by immunofluorescence staining of β-tubulin in post-confluent cultures of PyMTneoLUC stable transfectants

A. NMuMG, **B.** PyMTneoLUC_shNC, **C.** PyMTneoLUC_shAnxa1_1, and **D.** PyMTneoLUC_shAnxa1_4 cell lines were cultured for 4 days post confluence and stained for nuclei (DAPI, blue, left panels), β-tubulin (red, middle panels), and the merged images are also shown (right panels). **B.** An epithelial acinus formed by PyMTneoLUC_shNC cells is circumscribed with a dashed line. **C,D.** Examples of prominent β-tubulin-positive perinuclear microtubule organizing centres (MTOCs) are indicated with arrows. Scale bars 50μm.

Figure S16: Western blot analysis of E-cadherin (A) and vimentin (B) expression in PyMTneoLUC (i), EO771.LMB (ii), and SUM149 (iii) stable transfectants

NMuMG was used as a positive control for E-cadherin (**Ai, Aii**), and a negative control for vimentin (**Bi, Bii**) expression. EMT6.5 was used as a negative control for E-cadherin (**Ai**), and positive control for vimentin (**Bi**) expression. MCF-7 and MDA-MB-231 lysates were used as negative and positive controls for human vimentin expression, respectively (**Biii**). Signals were quantified using NIH Image J software and expression normalized to that for β-actin. **Ai, Bi.** Expression was set to 1 in PyMTneoLUC_shNC. **Bii.** Vimentin expression was set to 1 in EO771.LMB_shNC. **Aiii.** E-cadherin expression was set to 1 in SUM149_shNC. MW. molecular eight markers.

Figure S17: qRT-PCR analysis of gene expression in SUM149_shNC and SUM149_shANXA1 cell lines

Gene expression was normalized to RPL37A and levels in SUM149_shNC were set to 1. Mean +/- SD (n=3). **A.** CDH1 (E-cadherin). **B.** CDH2 (N-cadherin). **C.** FN (fibronectin). **D.** TNC (tenascin C), **E.** CAV1 (caveolin 1), **F.** CDH11 (cadherin 11). **G.** EGFR (epidermal growth factor receptor). All assays were 1 Step TaqMan qRT-PCR reactions except **E.,** which was 2 Step SYBR qRT-PCR.

Figure S18: Immunohistochemical analysis of epithelial marker expression in whole PyMTneoLUC_shAnxa1_1 mammary tumours formed in immuno-deficient C57BL/6 mice

A. pan cytokeratin. **B.** cytokeratin 8-18. **C.** E-cadherin. Two representative tumours (**i, ii**) are shown per stain. The positive epithelial zone (E) and negative mesenchymal (M) zones are indicated. X represents a mixed epithelial/mesenchymal region. N, necrotic region. Bars (bottom left) indicate the scale.

Figure S19: Immunohistochemical analyses of normal mouse tissue

A. Cytokeratin 14 (K14). i. PyMTneoLUC_shAnxa1_1 tumour showing K14 positive basal epidermal cells (red arrow) and K14 positive sebaceous glands (black arrow) in mouse skin adjacent to tumour (T). **ii.** PyMTneoLUC_shAnxa1_4 tumour showing K14 positive basal mammary epithelial cells (arrows). **B. Pan cytokeratin. i.** Control PyMTneoLUC_shNC tumour showing pan cytokeratin-positive basal and luminal mammary epithelial cells (arrow) in a duct adjacent to tumour (T). **ii.** PyMTneoLUC_shAnxa1_1 tumour showing a pan cytokeratin positive mammary duct (arrow) within a mesenchymal region of the tumour (T). **C. Cytokeratin 8-18 (K8-18).** Control PyMTneoLUC_shNC tumour showing K8-18 positive luminal mammary epithelial cells (arrows). Scale bars represent 200μm.

Figure S20: Cell surface expression of markers of tumour initiating cells in PyMTneoLUC transfectants

i. Control PyMTneoLUC_shNC, **ii.** PyMTneoLUC_shAnxa1_1, and **iii.** PyMTneoLUC_shAnxa1_4 cultured cell lines were analysed by flow cytometry for cell surface expression of CD44 and CD24.

Figure S21: Original Western blot images for Figure 2B

Figure S22: Original Western blot images for Figure S16

Table S1: List of primer sequences for SYBR green qRT-PCR

Supplementary References

1. Neve RM, Chin K, Fridlyand J, Yeh J, Baehner FL, Fevr T, Clark L, Bayani N, Coppe JP, Tong F *et al*: **A collection of breast cancer cell lines for the study of functionally distinct cancer subtypes**. *Cancer Cell* 2006, **10**(6):515-527.
2. Johnstone CN, Pattison AD, Gorringe KL, Harrison PF, Powell DR, Lock P, Baloyan D, Ernst M, Stewart AG, Beilharz TH *et al*: **Functional and genomic characterisation of a xenograft model system for the study of metastasis in triple-negative breast cancer**. *Dis Model Mech* 2018, **11**(5).
3. Johnstone CN, Smith YE, Cao Y, Burrows AD, Cross RS, Ling X, Redvers RP, Doherty JP, Eckhardt BL, Natoli AL *et al*: **Functional and molecular characterisation of EO771.LMB tumours, a new C57BL/6-mouse-derived model of spontaneously metastatic mammary cancer**. *Dis Model Mech* 2015, **8**(3):237-251.
4. Johnstone CN, Pattison AD, Harrison PF, Powell DR, Lock P, Ernst M, Anderson RL, Beilharz TH: **FGF13 promotes metastasis of triple-negative breast cancer**. *Int J Cancer* 2020.
5. Tikoo A, Roh V, Montgomery KG, Ivetac I, Waring P, Pelzer R, Hare L, Shackleton M, Humbert P, Phillips WA: **Physiological levels of Pik3ca(H1047R) mutation in the mouse mammary gland results in ductal hyperplasia and formation of ERalpha-positive tumors**. *PLoS One* 2012, **7**(5):e36924.
6. Owens RB, Smith HS, Hackett AJ: **Epithelial cell cultures from normal glandular tissue of mice**. *J Natl Cancer Inst* 1974, **53**(1):261-269.
7. Ralph P, Nakoinz I: **Phagocytosis and cytolysis by a macrophage tumour and its cloned cell line**. *Nature* 1975, **257**(5525):393-394.
8. Raschke WC, Baird S, Ralph P, Nakoinz I: **Functional macrophage cell lines transformed by Abelson leukemia virus**. *Cell* 1978, **15**(1):261-267.
9. Fidler IJ, Nicolson GL: **Organ selectivity for implantation survival and growth of B16 melanoma variant tumor lines**. *J Natl Cancer Inst* 1976, **57**(5):1199-1202.
10. Ma Y, Yamazaki T, Yang H, Kepp O, Galluzzi L, Zitvogel L, Smyth MJ, Kroemer G: **Tumor necrosis factor is dispensable for the success of immunogenic anticancer chemotherapy**. *Oncoimmunology* 2013, **2**(6):e24786.
11. Schneider CA, Rasband WS, Eliceiri KW: **NIH Image to ImageJ: 25 years of image analysis**. *Nature methods* 2012, **9**(7):671-675.
